# Uranyl(VI) Interaction with 2-Phosphonobutane-1,2,4-Tricarboxylic Acid (PBTC): A Spectroscopic and Computational Study over a Wide pH Range

**DOI:** 10.3390/molecules30204144

**Published:** 2025-10-21

**Authors:** Jerome Kretzschmar, Anne Wollenberg, Ion Chiorescu, Sven Krüger, Ronja Kraft, Michael U. Kumke, Satoru Tsushima, Katja Schmeide, Margret Acker

**Affiliations:** 1 Institute of Resource Ecology, Helmholtz-Zentrum Dresden-Rossendorf, 01328 Dresden, Germany; s.tsushima@hzdr.de (S.T.); k.schmeide@hzdr.de (K.S.); 2 Chair of Radiochemistry and Radioecology, Technical University Dresden, 01062 Dresden, Germany; a.wollenberg@hzdr.de; 3 Department of Chemistry, School of Natural Sciences, Technical University of Munich, 87547 Garching, Germany; chiorescu@mytum.de (I.C.); krueger@ch.tum.de (S.K.); 4 Institute of Chemistry, Faculty of Science, University Potsdam, 14476 Potsdam, Germany; kraft2@uni-potsdam.de (R.K.); kumke@uni-potsdam.de (M.U.K.); 5 Laboratory for Zero-Carbon Energy, Institute of Science Tokyo, Tokyo 152-8550, Japan; 6 Radiation Protection and Central Radionuclide Laboratory, Technical University Dresden, 01062 Dresden, Germany

**Keywords:** uranium, phosphonocarboxylate, speciation, complexation, NMR, UV-Vis, TRLFS, DFT, Raman

## Abstract

Organophosphonates have manifold applications in the chemical industry, of which one of the most commonly used is 2-phosphonobutane-1,2,4-tricarboxylic acid (PBTC). It is widely used as a cement additive and may pose a potential risk of complexing radionuclides such as uranium in nuclear waste repositories. PBTC, in its fully deprotonated form, has four negatively charged groups, one phosphonate and three carboxylate groups, which makes it a superior ligand for metal ion complexation. In this study, for the first time, its complexation behavior towards hexavalent uranium, U(VI), in the pH range from 2 to 11, has been investigated using various spectroscopic methods. The structure-sensitive methods NMR, IR, and Raman spectroscopy were used to characterize the complex structure. The interpretation of the results was supported by density functional calculations. Over almost the entire pH range studied, U(VI) and PBTC form a chelate complex via the phosphonate and the geminal carboxylate group, highlighting the strong chelating ability of the ligand. UV-Vis spectroscopy combined with factor analysis was applied to determine the distribution of differently protonated chelate species and their stability constants. Time-resolved laser-induced luminescence spectroscopy (TRLFS) was additionally used as a complementary method.

## 1. Introduction

There is a global consensus that high-level radioactive waste can only be safely disposed of in deep geological formations. Regardless of the host rock, various organic materials of anthropogenic origin will be introduced into such a nuclear repository, mainly via the radioactive waste matrix or the construction materials of the technical/geotechnical barriers, e.g., cement-based materials like concrete. In particular, organic additives, which are commonly added in large quantities to the cement-based materials, play a crucial role in optimizing their physical properties [1,2]. The influence of these anthropogenic organic compounds on the speciation of uranyl (UO_2_^2+^) and other radionuclides, and consequently on their migration behavior, is an important aspect of the long-term safety analysis for a repository. A critical worst-case scenario considered in the long-term safety assessment is the ingress of moisture. In this scenario, groundwater and leachate will enter the repository and finally cause cement degradation over an extended period of time. As a result, organic cement additives can be leached out and subsequently interact with potentially released radionuclides.

2-Phosphonobutane-1,2,4-tricarboxylic acid (PBTC) is one of the most commonly used organophosphonic acids in industry, and an important representative of cement additives based on phosphonocarboxylic acids [3,4,5,6,7]. It serves not only as an effective long-term retarder in concrete and as a corrosion inhibitor in reinforced concrete and steel but also as an efficient dispersant in water treatment plants and as an antiscalant in cooling water circuits [5,8,9,10]. The wide application range of PBTC is due to its excellent complexing ability with various metal ions (e.g., Ca^2+^, Cd^2+^, Pb^2+^, Zn^2+^, Mg^2+^, Cu^2+^, Al^3+^, and Fe^3+^) [4,11,12,13,14,15] owing to its four functional groups, comprising one phosphonate and three carboxylic groups.

To our knowledge, the aqueous complexation of PBTC with elements of the f-block, in particular U(VI), has not yet been investigated in any study. In contrast to the numerous studies on PBTC complex formation with spherical metal ions [4,11,12,13,14], the uranyl dioxo cation can only be coordinated in the equatorial plane perpendicular to the O=U=O axis, resulting in a unique coordination environment.

Since no data on U(VI) complexation with PBTC are available, as a first step, it is helpful to consider the known interactions between uranyl and other phosphonocarboxylates such as phosphonoformate, phosphonoacetate, phosphonopropionate, and also the well-known glyphosate bind to U(VI) in a chelating fashion, involving 5-, 6-, or 7-membered ring structures [16,17]. Since PBTC is unique in its constitution, i.e., one phosphonate group and three carboxyl groups, a rigorous examination is required to determine whether and to what extent exclusive coordination of any functional group or combinations of them is present.

Therefore, the aim of this study is to achieve the first species distribution, covering a wide pH range from 2 to 11, and to identify structures of the formed U(VI)-PBTC complexes, as well as to determine the corresponding complex stability constants. Species distribution studies of the complexes were carried out using UV-Vis, Raman, and time-resolved laser-induced luminescence (TRLF) spectroscopies, complemented by nuclear magnetic resonance (NMR) spectroscopy. The molecular structure of the U(VI)-PBTC complexes was investigated using multinuclear (^1^H, ^13^C, ^31^P, and ^17^O) NMR spectroscopy as the primary method, supplemented by attenuated total reflection Fourier-transform infrared (ATR FT-IR) spectroscopy and density functional (DF) calculations.

## 2. Results and Discussion

**Figure 1:**
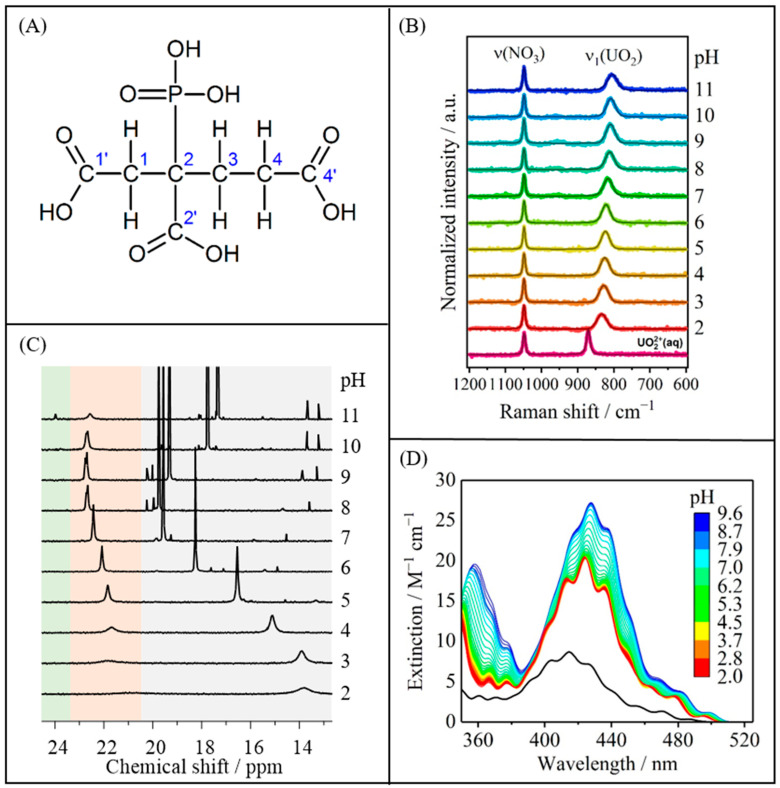
(**A**) Generic structure of the ligand PBTC and its pH-dependent U(VI) complexation analyzed by various spectroscopic methods. (**B**) Raman spectra obtained at a U(VI):PBTC ratio of 1:7 ([U(VI)] = 10 mM, *I*_m_ = 0.5 m NaCl), where the first spectrum from below is free uranyl(VI) in the absence of PBTC at pH 2. (**C**) ^31^P NMR spectra acquired at a given U(VI):PBTC ratio of 1:7 ([U(VI)] = 40 mM). (**D**) UV-Vis spectra measured at a U(VI):PBTC ratio of 1:3.5 ([U(VI)] = 10 mM, *I*_m_ = 0.5 m NaCl), where the black line represents the spectrum of free uranyl(VI) in the absence of PBTC at pH 2.

All four functional groups of PBTC constitute potential coordination sites for metal cations (Figure 1A). Generally, various U(VI)-PBTC complexes are conceivable depending on given conditions such as pH or metal-to-ligand ratio. In this work, we focus on complexation studies at ligand excess to suppress competing hydrolysis reactions at elevated pH and to prevent the formation of precipitates. Overview measurements under equimolar conditions and for U(VI) excess indicated formation of polynuclear species and/or precipitation, respectively. Under the latter conditions, the speciation appears to be very complex, and analyses also appear to be truly complicated; we thus waived extensive discussions and will only briefly address some key observations in this publication. The understanding of the aquatic interactions of PBTC with U(VI) in the pH range between 2 and 11 is achieved through the following:

I. Investigation of complex speciation: Estimation of the number and distribution of the formed complexes using UV-Vis, Raman, TRLF, and NMR spectroscopies (Section 2.1).
II. Structure elucidation: Determination of the main binding motif involved in complexation using NMR, ATR FT-IR, and DF methods (Section 2.2).
III. Determination of thermodynamic data: Complex formation constants derived from UV-Vis spectra combined with species characterization (Section 2.3).
IV. Consideration of additional minor U(VI)-PBTC complex species (Section 2.4).

### 2.1. U(VI)-PBTC Complex Speciation

This issue has been studied by combining data from UV-Vis, Raman, and NMR spectroscopies over a wide pH range from 2 to 11. However, these methods require relatively high U(VI) concentrations in the millimolar range, leading to certain limitations. For example, at equimolar ratios or in the presence of excess metal ions, hydrolysis of U(VI) induces precipitation of uranyl hydroxides at pH > 4. Therefore, experiments comprise two principal approaches: concentration-dependent series titrating PBTC to U(VI) at sufficiently low pH (hereafter referred to as *concentration series*) and pH titration series with a given excess of ligand (hereafter referred to as *pH series*).

Raman spectroscopic investigations were performed for the pH series with a seven-fold excess of PBTC across a pH range of 2 to 11 (Figure 1B) and for the concentration series at a constant pH of 2, provided as Figure S1 in the Supporting Information (SI). Reference spectra of PBTC were recorded and subtracted from the U(VI)-PBTC spectra to ensure unambiguous identification of U(VI)-related Raman features.

In agreement with the literature, the symmetric O=U=O stretching vibration mode ν_1_ of the uranyl aquo ion was observed at (871 ± 1) cm^−1^ [18,19,20]. At pH 2, the complexation between U(VI) and PBTC already occurs at U(VI):PBTC ratios below 1:1, as indicated by a pronounced bathochromic shift of approximately 20 cm^−1^ in the Raman vibration at a U(VI):PBTC ratio of 1:0.5 (see Table S1A, SI). This shift in the O=U=O stretching vibration is typically attributed to a weakening of the U=O bond upon ligand coordination [21,22]. With an increasing U(VI):PBTC ratio up to 1:5, the Raman vibration undergoes a further successive bathochromic shift, reflecting progressive complexation. Beyond this ratio, no significant change in the Raman shift is observed, suggesting the completion of complex formation. An initial estimation of the number of coordinated ligands (*n*) can be derived using the empirical relationship between the maximum Raman shift (ν_1,max_) at the U(VI):PBTC ratio of 1:5 and the number of equatorially coordinated ligands, as described by Nguyen-Trung et al. (Equation (1)) [18,23]:

ν_1,max_ (cm^−1^) = 871 − Δν_1_⋅*n* (1)

where *n* is the number of equatorially coordinated ligands and Δν_1_ is the incremental Raman band displacement (slope) in cm^−1^_._

Calculated incremental Raman shift changes (Δν_1_) for assumed coordination numbers *n* ranging from 1 to 5 and the corresponding calculated Raman shift ν_1_ predicted for a potential 1:1 complex at pH 2 are given in Table S1B, SI. Analysis of the results suggests that coordination of three or more PBTC ligands is unlikely, as the calculated Raman shifts for a potential 1:1 complex are significantly smaller than the experimentally observed shifts for U(VI):PBTC ratios of 1:0.5 to 1:2 at pH 2, which are around (850 ± 1) cm^−1^ (Table S1A, SI). If only the 1:1 complex were present, the Raman shift would be significantly larger at low U(VI):PBTC ratios, reaching approximately 833 cm^−1^, which is not observed experimentally. The experimental data indicate *n* = 2 as the most plausible maximum number of coordinated ligands. At the given pH of 2, where deprotonation is relevant only for the coordinating sites, for increasing PBTC concentration, the order of predominating species is, besides the free uranyl ion, the 1:1 complex, UO_2_(H_3_PBTC)^0^, and the 1:2 complex, UO_2_(H_3_PBTC)_2_^2−^ (see below), corresponding to Raman bands observed at 850 and 833 cm^−1^, respectively.

For the PBTC excess system (Figure 1B), upon increasing pH, ν_1_(UO_2_) shifts to lower wavenumbers almost linearly with a slope of about −3.2 cm^−1^ per pH unit, ranging from 833 cm^−1^ at pH 2 to 804 cm^−1^ at pH 11 (Table S1C, SI). This systematic decrease in the Raman shift indicates a successive change in the speciation of the U(VI)-PBTC complexes with an increasing electronic (charge) effect due to different protonation states in the complex molecule [24,25]. In comparison, the average pH-induced shift is substantially smaller than the Raman band shift associated with successive ligand coordination. Therefore, once the 1:2 U(VI)-PBTC complex is formed, fundamental changes in the U(VI) coordination environment, such as alterations in the number or coordination mode of bound PBTC due to changes in pH, can be ruled out. Furthermore, there is no indication for the formation of pure uranyl(VI) hydroxo or carbonato complexes at alkaline pH, as corroborated by NMR spectroscopy (see below).

Moreover, the pH-dependent trend of the directly measured Raman modes (Figure 1B, and Table S1C, SI) agrees well with the trend of the average energy gap calculated from the spacing of neighboring main peaks (i.e., vibrational fine structure in emissive transitions) in the pH-dependent U(VI)-PBTC luminescence spectra at the initial delay time 0.5 µs, correlating with the Raman-active symmetric stretching vibration ν_1_(UO_2_) [26,27] (Figure S2 and Table S2, SI).

The results from UV-Vis spectroscopy agree well with those obtained from Raman spectroscopy. Again, upon titrating PBTC to U(VI), there is a significant change in the absorption spectrum of U(VI)-PBTC samples, revealing significant alterations as compared to the spectrum of free UO_2_^2+^, indicating progressive complex formation (Figure S3, SI). At pH 2, beyond a threefold excess in PBTC, the spectra display virtually no further changes. Complex formation results in an approximately threefold increase in absorption and a notable bathochromic shift of approximately 9 nm. At a fixed 3.5-fold excess in PBTC given in solution, pH titration translates into a monotonic red-shift and, especially at pH > 5, into an increase in absorption in the spectra as shown in Figure 1D. For instance, the wavelength of maximum absorptivity, λ(ε_max_), shifts from 424 to 428 nm, and the maximum molar absorptivity, ε_max_, increases from 20.7 to 28.2 M^−1^ cm^−1^, when increasing pH from 2 to 10 (Figure 1D). This behavior indicates minor changes in speciation. Furthermore, no indications for the formation of hydroxo- or (hydroxy)carbonato-uranyl species were observed. The UV-Vis spectra of such species differ significantly from those of the U(VI)-PBTC complexes observed here, typically exhibiting less pronounced or altered vibronic fine structure and substantially higher molar extinction coefficients [28]. A control experiment conducted under an inert gas atmosphere yielded identical spectra to those recorded under ambient conditions, thereby excluding any influence of atmospheric CO_2_ on the observed speciation. Analysis of the spectra of both series by Iterative Transformation Factor Analysis (ITFA) [29] using principal component analysis (PCA) [30] followed by VARIMAX rotation [31] provided a model-independent speciation-like plot of the factor loadings of eight components (i.e., U(VI) species) as a function of PBTC concentration and pH. Further details on the procedure are stated with Figures S4 and S5 as well as Table S3, SI. Based on the Raman results, we assign the components of the concentration series to free uranyl, a 1:1, and a 1:2 U(VI)-PBTC complex. From the modest but continuous spectral changes observed consistently along the pH series in both Raman and UV-Vis spectroscopies, we infer that the 1:2 U(VI)-PBTC complex undergoes stepwise deprotonation of the non-coordinating functional groups, resulting in the formation of up to five additional species. Each of these species corresponds to a different protonation state of the 1:2 U(VI)-PBTC complex.

To assign the components determined with PCA, one can use the p*K*_a_ values of the PBTC ligand. Considering the protonated ligand as H_n_PBTC where n enumerates the number of protons, the five corresponding p*K*_a_ values determined in a 0.5 m NaCl solution are 0.9, 3.9, 4.8, 6.1, and 9.8 [32]. At first, the phosphonate group deprotonates once, followed by deprotonation of the carboxyl groups, and finally, the second deprotonation of the phosphonate group. These p*K*_a_ values will be lowered for PBTC ligands in the uranyl complexes due to the positive charge of the uranyl ion (metal-ion-promoted ligand deprotonation) [33]. To determine this decrease in p*K*_a_ values, we carried out density functional (DF) calculations to estimate deprotonation energies of the complexes UO_2_(H_4_PBTC)^+^, UO_2_(H_3_PBTC)^0^, and UO_2_(H_5−*a*_PBTC)(H_5−*b*_PBTC)^2−*a*−*b*^ for selected combinations of (*a*,*b*) = (4,3), (3,3), and (3,2). These combinations represent possible 1:1 and 1:2 complex stoichiometries under the lowest experimental pH condition (pH~2) (details in SI). These calculations show that, upon coordination to U(VI), the deprotonation of a H_4_PBTC^−^ ligand is lowered to about a pH of 1.5 (Δp*K*_a,2_ ≈ −2.4) and for H_3_PBTC^2−^ to a pH of about 3 (Δp*K*_a,3_ ≈ −1.8). Thus, at the experimental pH of 2, most of the H_4_PBTC^−^ ligands are deprotonated as H_3_PBTC^2−^, which dominates the speciation. This result suggests identifying the U(VI)-PBTC complexes appearing in the deconvolution of the spectra of the concentration series at pH = 2 as UO_2_(H_3_PBTC)^0^ and UO_2_(H_3_PBTC)_2_^2−^ (components 2 and 3 in Figure S5, SI). Among the spectra recorded at various pH values, the spectra with ligand excess at pH 2 are dominated by component 3, which is assigned to the complex UO_2_(H_3_PBTC)_2_^2−^ (Figure S5, SI). This assignment is further supported by the relatively low calculated complexation energies of U(VI) complexes with H_4_PBTC^−^ compared to H_3_PBTC^2−^ (cf. Table 3, Section 2.2 below). At pH = 2 and with ligand excess, a 1:2 complex UO_2_(H_4_PBTC)(H_3_PBTC)^−^ is also unlikely because the complexation-promoted deprotonation of H_4_PBTC^−^, leading to the 1:2 complex UO_2_(H_3_PBTC)_2_^2−^, is calculated to be exothermic (Table 3). With increasing pH, the further spectral components determined are assigned to a series of 1:2 complexes with successive deprotonation of the ligands up to UO_2_(HPBTC)(PBTC)^7−^ corresponding to component 8 appearing in significant concentration at a pH < 10 (Figure S5, SI). Despite an (at least) 3.5-fold excess of PBTC over U(VI) in the experiments, 1:3 complexes are considered negligible, as the results of Raman spectroscopy underscore 1:2 complexes (see above) and DF calculations show that the addition of H_3_PBTC^2−^ to UO_2_(H_3_PBTC)_2_^2−^ is an endothermic reaction (51 kJ∙mol^−1^, cf. Table 3).

UV-Vis and Raman experiments were complemented by TRLFS measurements to obtain additional information about the speciation. TRLFS is a well-established method for speciation analysis of uranyl in solutions and solids [34,35,36,37,38,39,40]. However, since photo-excited uranyl is a strong oxidant [41,42], oxidation-induced degradation of organic ligands, such as PBTC in this study, limits its applicability. To minimize this effect, the TRLFS experiments were performed under cryogenic conditions, which reduced photodegradation to 10–15% over the course of a typical measurement, as indicated by the decrease in emission intensity (see Figure S6, SI).

**Figure 2:**
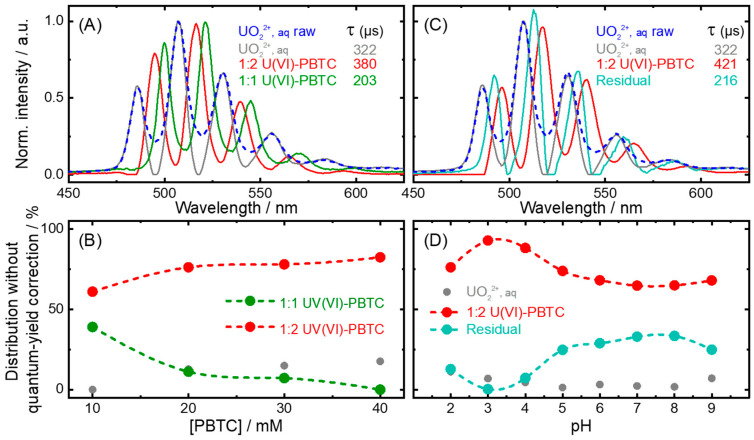
(**A**) Emission spectra of different U(VI) species obtained at *T* = 4 K, derived from a PARAFAC of the PBTC concentration series at pH = 2, and corresponding species distribution as a function of [PBTC] (**B**). (**C**) Emission spectra of different U(VI) species at *T* = 4 K, derived from a PARAFAC of the pH-series at a fixed U(VI):PBTC molar ratio of 1:5, and corresponding species distribution as a function of pH, [U(VI)] = 10 mM (**D**). The pure uranyl aquo ion has been included in the PARAFAC as a fixed species and compared to the non-PARAFAC-calculated spectra (UO_2_^2+^, aq raw, in blue). Color codes in (**B**) and (**D**) correspond to species represented by the same colors (**A**) and (**C**).

Two different sample sets were measured with TRLFS and analyzed with PARAFAC. The first set varied the PBTC concentration at pH 2 with a fixed uranyl concentration (Figure 2A,B), while the second set examined pH effects at a fixed U(VI):PBTC ratio of 1:5 (Figure 2C,D).

In line with our findings from Raman, UV-Vis, and NMR experiments (NMR results described below), we attributed the spectral components in Figure 2A to the uranyl aquo ion (gray) along with the 1:1 (green) and 1:2 U(VI)-PBTC complex (red), distinguished by the pronounced bathochromic shifts of 15 and 10 nm, respectively, compared to the aquo ion [43,44]. In the course of analyzing the time-resolved data, decay times (τ) and species distribution (Figure 2B) are obtained in addition to the single-component spectra. By increasing the PBTC concentration for a given pH of 2, the fraction of the 1:1 U(VI)-PBTC complex decreases, and that of the 1:2 complex increases. Keeping the U(VI):PBTC ratio fixed at 1:5 and altering the pH, we reasonably expect the 1:2 complex (red) to be the main species affected (Figure 2C,D). However, we now retrieve an additional spectral component—denoted “Residual” (Figure 2C, cyan), the origin of which is not clear. It could be a photodegradation product or due to the presence of impurities in the PBTC [9]. It seems that with increasing pH, its overall contribution is also increasing. It has to be kept in mind that other species (e.g., 1:1 complex observed at low pH) might also be present in the samples. However, due to low luminescence quantum yields and/or low overall concentrations, the PARAFAC based on the available data was not able to further resolve the residual signal into further single-component spectra, which limits the quantification of the different species. An alternative explanation could also be based on limitations in the PARAFAC with a limited data set: a small variation in the 1:2 complex (distribution) is translated into a single-component spectrum representing the average spectrum, with the variations reflected as the residual.

Comparing Figure 2A,C, both the bathochromic shifts (see Table 1) and the decay times (viz. 380 and 421 µs, both of which are larger than that of the free uranyl aquo ion) are in fair agreement, and we can make a strong argument towards our identification of the species. Further details are stated in Figure S7, SI.

Complementary to the other methods, NMR spectra of the U(VI)-PBTC system provide information from both the ligand’s (^1^H, ^13^C, ^31^P) and from the metal ion’s (^17^O) perspective. Comprehensive NMR data showcasing signal assignments, as well as supporting details on complex structures and dynamics, are provided as supplementary material compiled in Figures S10–S30, SI. This also includes exemplary spectra of samples with a near-equimolar U(VI):PBTC ratio and excess U(VI) given in solution.

The ^31^P nucleus of the phosphonate group constitutes a valuable and sensitive probe to comprehend the interaction between the ligand and U(VI), shown to be a persisting binding site throughout all complexes.

Extensive ^1^H, ^31^P, and ^13^C NMR pH-titration data of PBTC (along with assignment of several impurities apparently inherent in all commercially available products) by Kretzschmar et al. [32] now serve as reference spectra for the complexation studies, allowing for the identification of PBTC in its free and bound state. As exemplarily depicted in Figure 1C for pH-dependent U(VI)-containing samples with a given sevenfold excess in PBTC, the phosphonate-characteristic region can be divided into three ranges. That is, it constitutes a pH-dependent signal of unbound PBTC at *δ*_P_~13–20 ppm (gray, including several signals associated with impurities) and signals of U(VI)-bound PBTC at *δ*_P_~20–24 ppm. The latter comprises the orange region (*δ*_P_~20–23 ppm), highlighting the main species persisting over the entire pH range investigated, and the green region, which indicates a species forming exclusively under alkaline conditions (*δ*_P_~24 ppm).

As already reported [32], the unbound (excess) PBTC signal’s displacement upon increasing pH mirrors the various deprotonation steps of the ligand’s functional groups. The signal of U(VI)-bound PBTC is well-separated from that of the free ligand and always downfield, as caused by the high positive effective charge [45] of the uranyl(VI) ion. The very broad signals for both free and bound ligand observed at pH 2 indicate exchange dynamics owing to kinetically labile binding associated with competition between U(VI) and H^+^ for the binding at the carboxyl group (see below). Upon increasing pH, the signal sharpens because of the kinetically more stable ligand binding to U(VI). As compared to the ^31^P signal of the unbound PBTC (Figure 1C, gray region), that of the U(VI)-bound ligand shows much smaller variance in observed chemical shift (orange region), revealing only minor displacements of the complex species’ signal along the pH series consistent with observation from Raman and UV-Vis spectroscopies. Therefore, we conclude from ^31^P NMR that the same binding motif between PBTC and U(VI) is retained over almost the entire pH range (2–11) with little spectral variation caused by changing the complexes’ overall charge upon deprotonation of the non-binding carboxyl groups. Further observations, such as the occurrence of a seemingly split complex signal between pH 8 and 10, as well as further examination of the samples at the limiting pH values, will be discussed in Section 2.4.

In summation, in samples of excess PBTC, all three methods show that, as a function of pH, the spectra undergo a continuous but modest variation instead of a principal change in their appearance at any certain point. Consequently, the U(VI) species forming with PBTC, once established, retains its principal binding motif over a wide pH range and exhibit successive deprotonation of the dominant complex species.

### 2.2. Characterization of the Principal U(VI)-PBTC Binding Motif

ATR FT-IR and NMR spectroscopy were utilized to examine the functional groups involved in and the binding motif formed in the complexation of U(VI) by PBTC.

For ATR FT-IR analyses, spectra of the ligand dissolved in NaCl background electrolyte solution were recorded in the pH range 2–10 in the absence (blank) and presence of U(VI). The 1610–1680 cm^−1^ spectral region suffers from severe interference with the solvent. Therefore, this range is neglected in the following discussions.

Spectra obtained from the pH series in the absence of U(VI) reflect the different protonation states of PBTC [32]. Assignment of the observed band maxima to the vibrational modes of the free ligand was based on frequencies determined by DF calculations (Figure S8, SI), which are in fair agreement with literature data (Table S4, SI). At low pH, predominant bands observed around 1715, 1410, and 1270 cm^−1^ represent the stretching and deformation modes, respectively, of protonated carboxyl groups [46,47,48]. As the pH increases, the intensity of these vibrational modes decreases due to the deprotonation of the carboxyl groups, while a band at 1556 cm^−1^ and a broad multimodal feature between 1399 and 1311 cm^−1^ appear. These are assigned to the antisymmetric and symmetric carboxylate stretching vibration, ν_as_(COO^−^) and ν_s_(COO^−^) of the unbound ligand, respectively (Figure S8, SI). It is not possible to clearly distinguish between the individual carboxylate groups of PBTC by means of ATR FT-IR spectra, as the frequencies of these modes are very similar. The 1200 to 850 cm^−1^ spectral region is indicative of the phosphonate group-associated modes [49,50] (Table S4, SI), whose pH-dependent changes are attributed to elimination of intramolecular hydrogen bonds caused by deprotonation of the carboxyl groups, as no alterations in the phosphonate group’s protonation state occur in the pH range studied by ATR FT-IR [32].

For the unambiguous assignment of complex-related IR bands, difference spectra were calculated by subtracting spectra of unbound PBTC from those of the corresponding U(VI)-containing PBTC samples (Figure S9, SI). As stated above, according to the carboxyl groups’ associated p*K*_a_ values being >3.9 [32], at pH 2, the ligand spectrum displays features only of protonated COOH groups. The corresponding difference spectrum (bottom spectrum in Figure S9A, SI), however, lacks these features, and instead, two vibrational modes appear at 1587 and 1350 cm^−1^ which are due to ν_as_(COO^−^) and ν_s_(COO^−^), respectively. Thus, in the presence of U(VI), even in an acidic medium, at least one carboxyl group is being deprotonated because of complex formation. The extent of spectral splitting, i.e., the difference in wavenumber of these modes, Δ$\tilde{\nu }$(ν_as_,ν_s_), provides information about the carboxyl group’s type of coordination. The Δ$\tilde{\nu }$ value of the uncomplexed ligand (obtained as an average from spectra at pH 5 and 8) serves as a reference in the present case (154 ± 4) cm^−1^. In general, the band separation decreases if carboxylate–metal ion coordination is bidentate, but increases if monodentate [51,52,53]. Δ$\tilde{\nu }$ is calculated as 237 cm^−1^ at pH 2 and increases further to 256 and 267 cm^−1^ at pH 5 and 8, respectively, indicating both monodentate O=CO-U(VI) coordination and the stability of this binding motif irrespective of pH. This observation indicates the presence of a consistent binding motif at the carboxyl group(s), which aligns with the findings from the speciation studies (Section 2.1). Furthermore, the difference spectra exhibit features in the 1200–850 cm^−1^ spectral region, associated with the phosphonate group, indicating its participation in U(VI) coordination (cf. Figure S9, SI). In addition, the antisymmetric stretching vibration of the uranyl unit, ν_3_(UO_2_), evidences U(VI) complexation. As compared to the uranyl aquo ion’s ν_3_(UO_2_) observed at 961 cm^−1^ [22,54,55], the bathochromic shift to 932 cm^−1^ clearly evidences PBTC complex formation. Taking into account the invariance of the binding motif as inferred from NMR in this pH range, the gradual shift to 915 and 899 cm^−1^ at pH 5 and 8, respectively, again mirrors the successive deprotonation of the carboxyl groups not involved in U(VI) binding. The accompanied cleavage of hydrogen bonds as well as increasing overall negative charge in the complex further increases the electron density surrounding the metal center, effectively weakening the U–O_yl_ bond, thereby reducing its force constant and lowering the vibrational frequency. Thus, the ATR FT-IR investigations not only provide clues on the binding motif but are also in good agreement with the speciation investigations.

**Figure 3:**
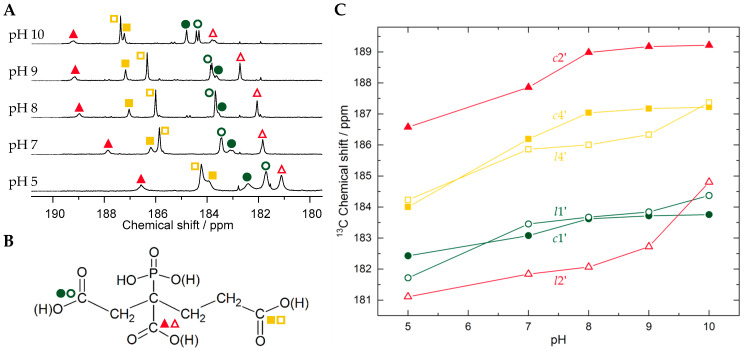
Carboxyl region of ^13^C{^1^H} NMR spectra obtained from solutions 40 mM in U(VI) and 290 mM in PBTC, at pH values stated with the spectra (**A**). The carboxyl groups in the free and U(VI)-bound ligand are denoted by open and filled symbols, respectively, according to the sites indicated in (**B**). (**C**) depicts the pH-dependent carboxyl carbons’ ^13^C chemical shifts in PBTC as free ligand (*l*) and complexing U(VI) (*c*).

In order to determine the carboxyl group(s) involved in U(VI) coordination, ^13^C NMR was performed. Unambiguous assignment of the carbons is accomplished by ^1^H–^13^C correlation spectra provided as supplementary material in the section *Analysis of NMR data to identify structure features* in the SI. Figure 3 depicts the carboxyl region of ^13^C NMR spectra along with a corresponding graph showing the pH-dependent carboxyl carbons’ ^13^C chemical shifts in PBTC as free ligand and complexing U(VI) (open and filled symbols, respectively). The largest U(VI) complexation-induced ^13^C NMR chemical shift changes (5.5–7.0 ppm, somewhat varying upon the pH-dependent protonation state, hence the charges of the other functional groups influencing the resulting signal position) are observed for carboxyl carbon C2′.

By contrast, the signals of carboxyl carbons C1′ and C4′ reveal much smaller chemical shift differences in PBTC carbons in the free and U(VI)-bound state. Both these facts evidence the participation of the C2′ carboxyl group in U(VI) binding via a six-membered ring chelation motif and the presence of this feature from acidic through alkaline conditions. Deviations of pH-dependent chemical shifts for C1′ and C4′ carboxyl carbons for free and U(VI)-bound PBTC arise from the individual chemical environments distinct from the free ligand, as well as complexation-induced p*K*_a_ changes for these non-complexing sites. Additionally, for corresponding pH, U(VI)-induced signal displacements of PBTC’s backbone carbons are rather small (Figure S12, SI). Of these, the largest effects, viz. 0.65 and 0.57 ppm, are observed for carbons C3 and C2, respectively, mainly caused by electronic effects (C2) and/or steric effects (C3) upon complexation and concomitantly changing conformation. Again, as sites C1 and C4 are distant and/or less sterically affected, their U(VI)-induced signal displacements are no larger than 0.1 ppm.

Possessing a chiral center at the asymmetric carbon C2, PBTC is present as a racemic mixture of the two (*R*) and (*S*) enantiomers. Consequently, in U(VI) complexes forming upon coordination of two ligands, in principle, two diastereomeric pairs of enantiomers are to be expected, viz. of configuration (*R*,*R*) and (*S*,*S*) as well as (*R*,*S*) and (*S*,*R*). Generally, these isomers can be distinguished by their relative position of the unbound CH_2_COO(H) (R^1^) and CH_2_CH_2_COO(H) (R^2^) residues (Figure S31, SI). This results in either *syn-* or *anti*-orientation of corresponding residues R^1^ or R^2^, with the resulting isomers distinguishable by ^17^O NMR spectroscopy.

Complementarily, within the plane perpendicular to the O=U=O axis, in 1:2 complexes, the two U(VI)-coordinating phosphonate groups (similar to the two involved C2′ carboxylate groups) can be in a *cis-* or *trans*-orientation relative to each other, resulting in a total of six irreducible distinct isomers (see Figure S31, SI). The functional groups’ *cis-* or *trans*-orientation is reflected in the presence of two distinct ^31^P NMR signals, one for each arrangement, with subtle differences in the local electronic environment of the phosphorus nuclei.

Note that we will use the terms *syn* and *anti* to refer to the relative position of the ligand residues R^1^ and R^2^, and the terms *cis* and *trans* to refer to the arrangement of corresponding functional groups (phosphonates as well as C2′ carboxyl). Consequently, four isomers that differ in these structural features can be distinguished by NMR: (*anti*, *trans*), (*anti*, *cis*), (*syn*, *trans*), and (*syn*, *cis*).

**Figure 4:**
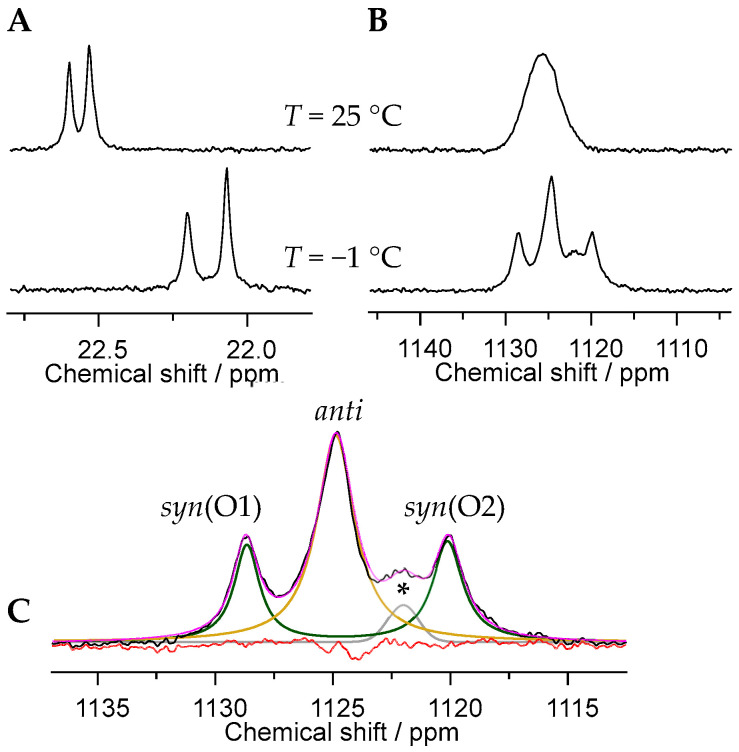
^31^P{^1^H} (**A**) and ^17^O NMR spectra (**B**) of a pH 8 solution 10 mM in U(VI) (enriched in ^17^O) and 50 mM in PBTC acquired at 25 °C (top) and −1 °C (bottom), along with spectral deconvolution of the uranyl ^17^O NMR signals obtained at −1 °C (**C**). For further details, see text.

To further corroborate the existence of isomers experimentally, we performed ^17^O NMR spectroscopy, particularly at low temperatures, to probe the uranyl oxygen (O_yl_) resonances. This approach allows us to distinguish between *syn-* and *anti*-orientations, as these influence the local symmetry and chemical environment of the O=U=O moiety. In the case of *anti*-orientation, where there are identical residues on opposite sides of the molecular plane, both O_yl_ atoms are identical and hence give rise to a single resonance. However, when the two identical R residues are positioned on the same side of the molecular plane (*syn*-orientation), the two O_yl_ are distinct and exhibit individual resonances of equal intensity as depicted in Figure 4B,C, denoted *syn*(O1) and *syn*(O2), respectively. Based on spectral deconvolution and subsequent signal area integration, and in line with steric and energetic expectations, the *anti*-orientation is slightly favored over the *syn*-orientation, with a ratio of 58:42. The ^17^O NMR spectrum obtained at −1 °C (Figure 4C) displays one additional, albeit minor signal (denoted by an asterisk), presumably due to a transient intermediate species involved in the interconversion between the isomers. These dynamics explain why the ^17^O NMR spectrum at room temperature reveals only one broad average signal (Figure 4B, top). Isomer interconversion accelerates with increasing temperature (cf. also ^13^C NMR spectra obtained at 0, 25, and 70 °C in Figure S14, SI).

Examination of the ^31^P NMR spectra obtained from pH-, concentration-, and U(VI):PBTC ratio-dependent series (Figure 1C and Figure S15–S18, SI), provides further insights into the dynamics of the *cis*–*trans* rearrangement. At acidic to near-neutral pH values (up to pH ~7.5), the U(VI)-PBTC complex species give rise to a single, but broadened, ^31^P NMR signal, independent of U:PBTC ratio and absolute concentration (Figure 1C and Figure S16A, SI). In contrast, under alkaline conditions, the spectrum exhibits a seemingly split signal. The two lines indeed correspond to distinct resonances arising from the presence of *cis* and *trans* ligand arrangements. This interpretation is supported by magnetic field-dependent ^31^P NMR measurements, which show that the respective chemical shifts (in ppm) remain invariant while the separation expressed in frequency units (in Hz) is field-dependent. Consequently, the apparent signal splitting cannot be attributed to scalar spin–spin coupling (*J*), as such couplings would yield a constant splitting in Hz regardless of field strength (cf. Figure S19, SI).

Line broadening or signal averaging indicating site exchange dynamics are observed not only upon temperature variation (Figure 4 and Figure S14, SI) but also at low pH, as evidenced by various NMR spectra (^1^H, ^13^C, ^31^P; cf. Figure 1C and Figure 3A, as well as Figures S10, S12A, S15, and S16, SI). We attribute the broadening at low pH to the competition between U(VI) and H^+^ for coordination at the functional groups, particularly at the carboxyl group C2′. This group has been shown to be a part of the chelation motif and has a p*K*_a_ of 6.1 in the free ligand, i.e., the highest among all carboxyl groups [32]. As inferred from ^1^H,^1^H-EXSY (exchange spectroscopy, Figure S21, SI), dynamics seem to comprise both intramolecular and intermolecular processes: site exchange may occur either unimolecularly, within the same molecule, or bimolecularly, via ligand displacement between free and bound ligand. In the alkaline medium, however, the two individual ^31^P resonances are well resolved, indicating a significant decrease in the ligand exchange dynamics. Site exchange might be compensated by (outer-spherical) sodium ions (vide infra), kinetically stabilizing the complexes’ conformation.

DF calculations of the fully protonated ligand, H_5_PBTC, showed essentially no energy differences (<1 kJ/mol) between its (*R*) and (*S*) enantiomers. This suggests that the observed difference in concentrations of diastereomers (i.e., diastereomeric excess) results from energy differences in the 1:2 complexes due to their composition by (*R*) and (*S*) enantiomers. As an example, we inspected the complex UO_2_(HPBTC)_2_^6−^, which is the prevailing species present in the pH range of approximately 7.5 to 9 (cf. Section 2.3 and Figure S5, SI, component 7). Calculations of compositions of this complex with varying enantiomers of HPBTC as (*R,R*) and (*S*,*S*) as well as (*R,S*) and (*S*,*R*), each with either a *cis* or *trans* arrangement of the two phosphonate groups yields in total six species, being distinguishable *cis*/*trans* pairs of *anti*- and *syn*-isomers, respectively (cf. Figure S31, SI). The energy differences between these isomers essentially vanish, not exceeding 1 kJ/mol (Table S5, SI). The largest energy difference between *cis* and *trans* structures of 0.9 kJ/mol is found for the (*S*,*S*) isomers, and all other variants show energies in the same interval. As the calculated energy differences between all isomers are smaller than the expected accuracy of the method and are essentially degenerate, they cannot be reliably interpreted. On the other hand, this overall degeneracy of all isomers of UO_2_(HPBTC)_2_^6−^ is consistent with and supports the small differences in concentrations between diastereomers seen in the ^31^P and ^17^O NMR measurements discussed above. However, a definitive assignment of the two distinct ^31^P signals to the *cis* and *trans* arrangements cannot be made at this point.

**Figure 5:**
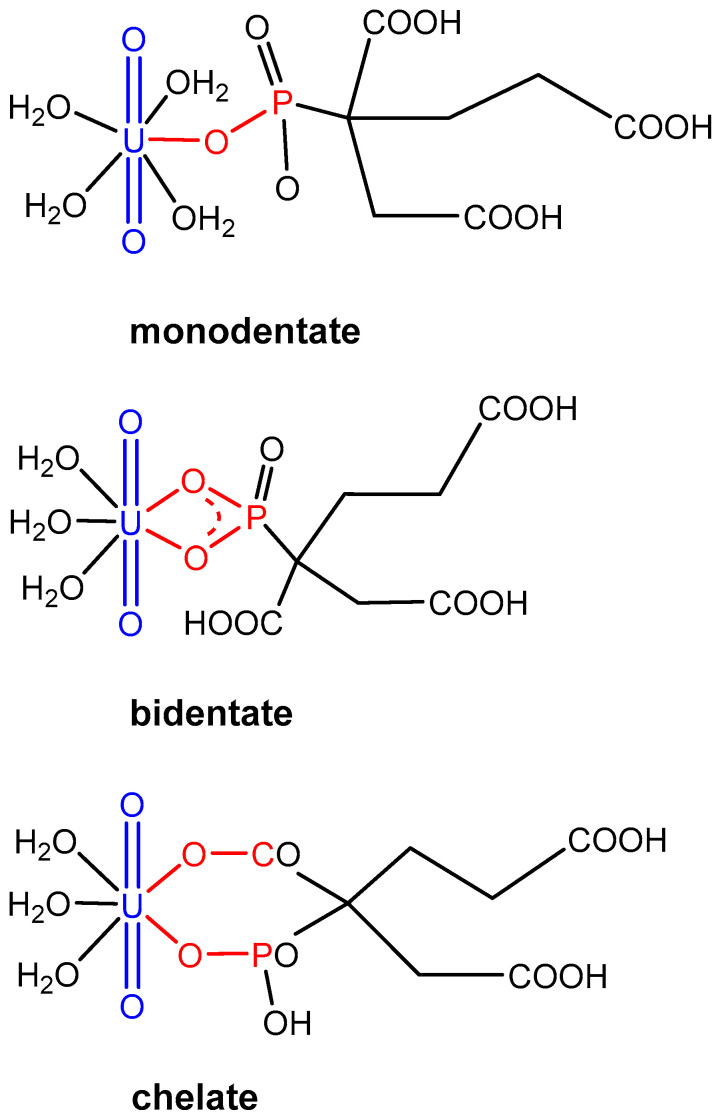
Coordination modes of PBTC to uranyl for the example of UO_2_(H_3_PBTC)^0^.

The DF calculations of the U(VI)-PBTC coordination modes (Figure 5, Table 2) confirm that PBTC favors chelate binding to uranyl over both mono- and bidentate coordination modes of only phosphonate. In a charge-neutral U(VI)-PBTC 1:1 complex, the chelate binding motif is energetically favorable by 37 or 43 kJ/mol compared to the mono- or bidentate coordination mode, respectively.

The chelation occurs via the phosphonate and the carboxyl group at the second position of PBTC, as concluded from ^13^C NMR. This preference is attributed to the formation of a stable six-membered ring structure, which enhances the stability of the complex. This chelation mode allows the ligand to effectively coordinate the metal ion, minimizing steric strain and maximizing binding affinity compared to alternative coordination configurations. In the following, we refer to the different protonation states of PBTC using the above-mentioned notation H*_n_*PBTC. H_5_PBTC designates the fully protonated charge-neutral ligand, while PBTC corresponds to the fully deprotonated ligand with a charge of −5 *e* at a very high pH. Table 2 summarizes the reaction energies for the formation of various neutral U(VI)-PBTC 1:1 complexes, as given by Equation (2). Isomers with different coordination numbers (CNs) around the metal ion were optimized, revealing that CN = 5 and 4 are most favorable for the stable chelate coordination of PBTC. Monodentate and bidentate binding are significantly less stable (cf. Figure 5).

H_3_PBTC^2−^ + UO_2_(H_2_O)_5_^2+^ ⇌ UO_2_(H_3_PBTC)(H_2_O)*_n_* + (5 − *n*)H_2_O (2)

Table 3 presents the Gibbs free energies of formation of various 1:2 uranyl-PBTC complexes, calculated based on reactions between uranyl and different protonation states of PBTC according to Equation (3).

x UO_2_^2+^ + y_1_ H_a_PBTC^(5−a)−^ + y_2_ H_b_PBTC^(5−b)−^ ⇌ (UO_2_)_x_(H_a_PBTC)_y1_(H_b_PBTC)_y2_^(2x+z−5y)^ (3)

where y = y_1_ + y_2_ denotes the total number of coordinated PBTC ligands and z = y_1_·a + y_2_·b represents the total number of protons.

**Table 3:** Gibbs free energies of formation in kJ/mol of various (UO_2_)_x_(H_a_PBTC)_y1_(H_b_PBTC)_y2_^(2x+z+5y)^ complexes, computed according to Equation (3).

Complex	U(VI)-Ligand-Proton	Δ*G*
UO_2_(H_4_PBTC)^+^	114	−41
UO_2_(H_3_PBTC)^0^	113	−119
UO_2_(H_4_PBTC)_2_^0^	128	−37
UO_2_(H_4_PBTC)(H_3_PBTC)^−^	127	−106
UO_2_(H_3_PBTC)_2_^2−^	126	−180
UO_2_(H_3_PBTC)(H_2_PBTC)^3−^	125	−216
UO_2_(H_2_PBTC)_2_^4−^	124	−278
UO_2_(H_1_PBTC)_2_^6−^	122	−294
UO_2_(H_3_PBTC)_3_^4−^	139	−129

These values demonstrate that the Gibbs free energy of formation, Δ*G*, for the uranyl complex decreases with the progressive deprotonation of the PBTC ligand, reflecting an increase in complex stability. The extent of ligand deprotonation in aqueous solution is governed by the pH. Thus, at pH values below 3.5, the anionic 1:2 complex, UO_2_(H_4_PBTC)(H_3_PBTC)^−^, with a free energy of formation of −106 kJ/mol, is slightly less stable than the neutral 1:1 complex, UO_2_(H_3_PBTC). This suggests that the neutral 1:1 complex is thermodynamically more preferred under strong acidic conditions than UO_2_(H_4_PBTC)(H_3_PBTC)^−^. The neutral 1:2 complex, UO_2_(H_4_PBTC)_2_, with Δ*G* of −37 kJ/mol, is significantly less stable than the neutral 1:1 complex. As a result, under acidic conditions (pH < 2.5), the neutral 1:1 complex, UO_2_(H_3_PBTC), predominates even with a moderate excess of PBTC in the solution. Regarding the 1:3 complex with a charge of −4 e, UO_2_(H_3_PBTC)_3_^4–^, its Δ*G* is considerably less favorable than that of the anionic 1:2 complexes with charges < −1 e. This is probably due to the enhanced steric repulsion between the bulky PBTC ligands rather than due to electrostatic repulsion between those ligands. The reason for this assumption is that all ligands in the 1:2 complex bear at least the same or an even more negative charge than in the 1:3 complex. Also, in the 1:3 complex, only two of the ligands show a chelated coordination, while the third one binds monodentate as a result of steric restrictions. Thus, the structure of the complex is in line with its rather low stability (Table 3). Consequently, the 1:3 complex UO_2_(H_3_PBTC)_3_^4–^ is expected to be a minority species in the solution, occurring only in trace amounts, if at all. For the strongly anionic complex UO_2_(H_2_PBTC)_2_^4–^, we suspected a possible stabilization by Na^+^ ions from the solution. The addition of two Na^+^ ions destabilized this complex by 58 kJ/mol. Even for anionic complexes with a higher negative charge, stable ternary complexes with Na^+^ ions in the inner-sphere are thus unlikely. For the strongly anionic U(VI)-PBTC complexes, charge compensation in solution appears to be more feasible in the form of an outer sphere cation coordination. Whereas three distinct Na^+^ environments were resolved for aqueous uranyl citrate sandwich complexes by ^23^Na NMR [56], analogous measurements for the U(VI)-PBTC system (even at −5 °C) revealed only one, but with a broadened NMR signal. This observation is in line with rapid exchange between outer-sphere Na^+^ ions and bulk Na^+^ aquo species.

In the following, we briefly discuss the binding motif in U(VI) complexes with chelating ligands structurally related to PBTC.

Single crystals obtained from hydrothermal syntheses display uranyl complexation with phosphonoacetate upon six-membered ring chelation by carboxylate and one phosphonate oxygen [57]. In solution, the three phosphonomonocarboxylates, phosphonoformate, phosphonoacetate (PAA), and phosphonopropionate (PPA), are reported to form 1:1 complexes with UO_2_^2+^ at pH 2, with the ligands being in their monoprotonated form [UO_2_(LH)], showing almost identical complex stabilities among each other. Corresponding chelates form five-, six-, and seven-membered rings, respectively, through the participation of one oxygen each from the carboxylate and phosphonate, involving an intramolecular hydrogen bond between one uranyl oxygen and the remaining proton of the phosphonate group [16]. Although complexation of U(VI) by PBTC’s phosphonate group and two of the three other carboxyl groups (i.e., in 1,1- and 1,2-position) yield the same structural chelation motif as in the above discussed U(VI) complexes of PAA and PPA, i.e., six-, and seven-membered rings, respectively, the six-membered ring is the only and persisting coordination motif. Intramolecular hydrogen bonding was also observed in the DF-calculated U(VI)–PBTC complex structures; however, in contrast to the phosphonomonocarboxylates, the hydrogen bond forms between the phosphonate group’s OH and the C4′ carbonyl oxygen rather than to a uranyl oxygen. This interaction is sterically preferred by about 24 kJ/mol and cannot occur in the case of the simpler phosphonomonocarboxylates. Though being a phosphonocarboxylate, glyphosate’s molecular structure is somewhat different from PBTC, as the phosphonate group is in 1,3-position relative to the carboxyl group and each of the former two is in 1,2-position to a secondary amine being involved in coordination, hence acting as a tridentate chelate forming two five-membered rings. Moreover, the two remaining coordination sites, in the 1:1 complex occupied by water ligands, can be displaced by one or two non-chelating ligands binding by the phosphonate group. Formation of these 1:2 and 1:3 complexes is detectable already for a mere 1.25-fold ligand excess. As determined from signal area integration of quantitative ^31^P NMR spectra with up to 18-fold ligand excess, signals associated with coordinated PBTC invariantly revealed two bidentately chelating ligands, consistent with the other spectroscopies and DFT. Compared to the glyphosate system, displacing the remaining one water ligand (given a CN of 5) is much less likely in the 1:2 U(VI)–PBTC complexes due to the sterically demanding and more negatively charged PBTC ligand. Similar to citrate [33] and glyphosate [17], PBTC also forms isomers upon complexation with uranyl(VI), as evidenced from NMR spectroscopy, especially when probing the uranyl oxygens by ^17^O NMR, giving rise to unique sets of signals per isomer. In summary, there are similarities and differences in the coordination behavior of PBTC in comparison to related molecules. However, PBTC is a polyfunctional ligand similar to the related ligands discussed yet unique in its constitution, i.e., it possesses one phosphonate group and three carboxyl groups in 1,1-, 1,2-, and 1,3-position, respectively. This makes PBTC the only phosphono*poly*carboxylate studied regarding U(VI) complexation. This requires rigorous examination of whether and to what extent exclusive coordination of any functional group or combinations of them, i.e., chelate motifs with variable ring size, is present.

### 2.3. Complex Stability Constant of the Main U(VI)-PBTC Complex

For the thermodynamic calculations of the complexation constants $\beta_{xyz}$, Equation (3) was reformulated to express the reaction between U(VI) and PBTC in terms of the basic species UO_2_^2+^, deprotonated PBTC ligand, and H^+^ as follows (Equations (4) and (5)):

x UO_2_^2+^ + y PBTC^5−^ + z H^+^ ↔ (UO_2_)_x_(PBTC)_y_(H)_z_^(2x+z−5y)^ (4)

where

(5) $$\beta_{xyz}=\frac{\left[(UO_{2}{)}_{x}(PBTC{)}_{y}(H{)}_{z}^{\left(2x+z-5y\right)}\right]}{[UO_{2}^{2+}{]}^{x}\cdot [PBTC^{5-}{]}^{y}\cdot [H^{+}{]}^{z}}$$

In the evaluation of the absorption spectra, both the concentration series at pH 2 and the pH titration series with an (at least 3.5-fold) excess of PBTC and varying U(VI) concentrations (0.5, 1, and 10 mM) were included. The spectrum of free UO_2_^2+^ at pH 2 was fixed in the calculations as a known input parameter. Based on the results of the ITFA (number of components, Section 2.1) and the characterization of the binding motif with PBTC excess (Section 2.2), a complexation model was formulated that includes a neutral 1:1 (at pH 2) and various 1:2 U(VI)-PBTC complexes with different deprotonation states of the PBTC ligand based on Equation (4). The complexation constants and the spectra of the single components obtained from the individual series have been averaged.

**Figure 6:**
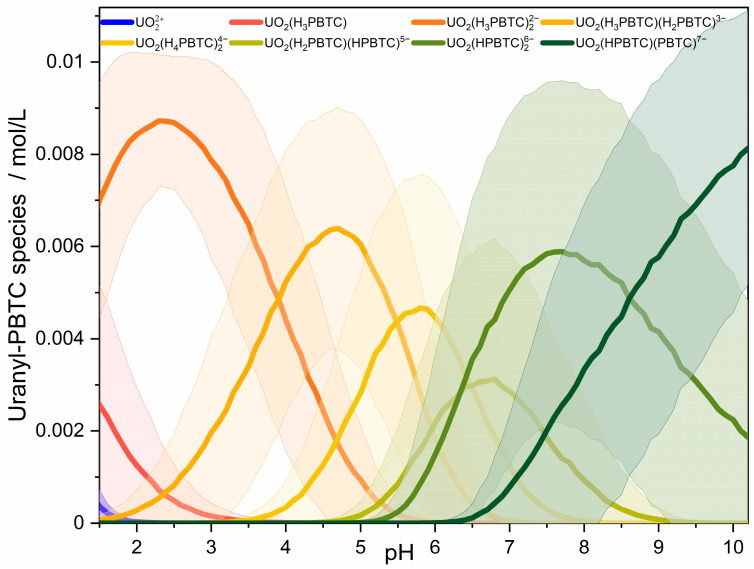
Species distribution diagram for the U(VI)–PBTC system, calculated as a function of pH for 10 mM U(VI) and 50 mM PBTC using the stability constants determined in this work with uncertainty propagation via the Monte Carlo simulation. For each pH step, from 1.5 to 10 in 0.1 increments, the stability constants were randomly sampled within their experimental error bounds. The bold curves represent the mean values of all successful Monte Carlo runs (5000 runs per pH step) with shaded error bands indicating the corresponding uncertainty (2σ).

The results show that the ligand concentration series at pH 2 can be well fitted with the formation of a UO_2_(H_3_PBTC)^0^ complex, followed by the UO_2_(H_3_PBTC)_2_^2−^ complex, while the pH titration series can be described by the successive deprotonation of UO_2_(H_3_PBTC)_2_^2−^. This is in agreement with our theoretical findings on the complex stability and ligand p*K*_a_ shifts upon complexation. Table 4 summarizes the corresponding stability constants log *β_xyz_*, while the resulting speciation, calculated using these constants, is presented in Figure 6. For better comparability of the stability constants, they have been converted to conditional log *K* values (cf. Table 4) according to Equation (3), taking into account the deprotonation reaction of PBTC with the protonation constants from Kretzschmar et al. [32].

In contrast to the theoretically calculated Gibbs free energies of complex formation (Table 3), the experimentally determined stability constants and corresponding free energies do not indicate an increase in complex stability. The values show no clear trend with respect to pH and the degree of ligand deprotonation. Instead, the data allow the determination of an average stability constant of log *K* = 9.7 ± 1.0 and a Gibbs free energy Δ*G* = − (55.5 ± 6.0) kJ/mol for the ensemble of species attributed to the U(VI)-PBTC bis-chelate 1:2 complex—regardless of the protonation state of carboxyl groups not involved in complexation. This finding highlights the structural invariance of the coordination motif, unaffected by variations in peripheral functional groups. That is, even upon deprotonation, the remaining carboxyl groups do not engage in intramolecular rearrangement processes such as carboxyl site exchange between the free and the U(VI)-bound carboxyl group. The approximate constant measured complexation strength along the 1:2 complex series can tentatively be traced back to charge compensation of the anionic complexes by Na^+^. The calculated increasing complexation strength (Table 3) reflects essentially only the electrostatic energy, increasing for increasingly more negative ligands along the series. As inner-sphere Na^+^ coordination to the anionic complexes can be excluded (see above), charge compensation appears via outer-sphere coordination of Na^+^, and the corresponding energy gain is compensated by a loss of entropy in the solution. Furthermore, the nearly isoenergetic 1:2 chelate species with coordination numbers of 5 and 4 (differing only by the presence or absence of a coordinating water molecule (cf. Table 2)) indicate that the U(VI) center is essentially saturated by the two PBTC ligands. As the corresponding electron density in the complexes increases with successive deprotonation, the affinity of U(VI) for a fifth coordinating ligand, such as OH_2_ or OH^−^ (the latter due to Coulomb repulsion), becomes even less favorable. This electronic shielding effectively prevents U(VI) hydrolysis, even at U(VI) concentrations up to 0.1 M and pH of ~10, and also suppresses the formation of polynuclear species (e.g., via olation and/or oxolation), provided PBTC is in excess.

The averaged absorption spectra of the extracted single-components and the corresponding wavelength at maximum extinction are shown in Figure S32 and Table S8, SI, respectively. The values for free U(VI) agree reasonably well with those reported in the literature for comparable conditions [58,59].

### 2.4. Expanded Insights into Uranyl(VI)–PBTC Speciation via Detailed ^17^O NMR

Compared with the spectra obtained for aqueous uranyl (hydrolysis) species (at both room and low temperature) [60,61], the ^17^O NMR signals of the uranyl oxygen clearly indicate that PBTC interacts with U(VI) already at pD 2 (Figure S11, SI). Similar to the changes observed in Raman and UV-Vis spectra under acidic conditions, the ^17^O NMR spectrum also reveals significant alterations. That means upon addition of PBTC, the uranyl oxygen signal shifts by 6.4 ppm (at 25 °C, *δ*_O_ 1117.0 → 1123.4 ppm). Although the signal shape becomes more asymmetric, indicating overlap of several individual lines, the competition between UO_2_^2+^ and H^+^ for PBTC binding is so pronounced and thus dynamic at such low pH that even spectra recorded at −5 °C remain poorly resolved. Attempts to deconvolute the spectra and assign individual species were unsuccessful.

Besides the species observed in acidic solution along with the main U(VI)-PBTC complexes discussed in detail up to alkaline media, one more species emerging above pH 9 was identified and characterized by distinct signals in the ^1^H, ^13^C, ^31^P, and ^17^O NMR spectra, providing clear evidence for its unique coordination environment. We assume this species to be a U(VI)-PBTC-hydroxo species, hereafter referred to as *ternary* species, U(VI)-PBTC-OH. The structural features and spectroscopic signatures of this complex are detailed below.

**Figure 7:**
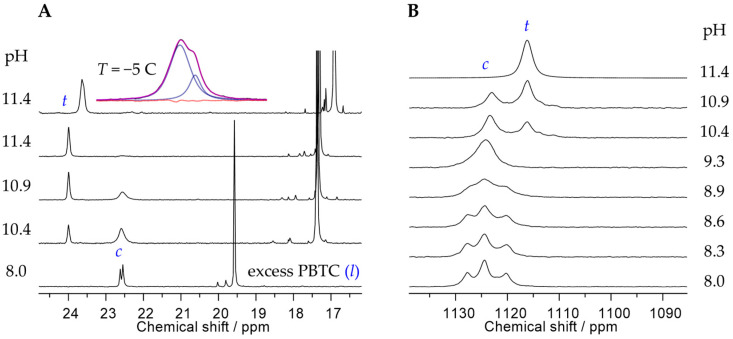
^31^P{^1^H} (**A**) and ^17^O NMR spectra ((**B**) region of uranyl oxygens) of 90/10 (*v*/*v*) H_2_O/D_2_O solutions 57 mM in U(VI) (enriched in ^17^O) and 200 mM in PBTC, obtained at varying pH. ^31^P NMR spectra were acquired at 25 °C, except for the pH 11.4 sample, which was also measured at −5 °C (top spectrum); a corresponding spectral deconvolution is given as an insert. All ^17^O NMR spectra were acquired at −5 °C. For further details, see the text.

Upon increasing pH beyond 9, the fraction of the ternary species (*t*) successively increases at the expense of the dominant 1:2 complex species (*c*), as observed in both the ^31^P and the ^17^O NMR spectra (Figure 7A and Figure 7B, respectively). We tentatively assign this ternary species to UO_2_(OH)(PBTC)_2_^9−^.

In contrast to the upfield shift of the ^31^P NMR signal associated with the second deprotonation of the phosphonate group in free PBTC (*l*) (*δ*_P_ = 19.6 → 17.4 ppm at 25 °C), the newly emerging ^31^P signal (*δ*_P_ = 24.0 ppm at 25 °C) is shifted downfield relative to the main 1:2 complex (*δ*_P_ = 22.6 ppm). Additionally, the low-temperature ^31^P signal still reveals two distinct resonances with a 77:23 area ratio (see insert in Figure 7A). This observation is plausible when considering that the OH^−^ ligand can be coordinated either *cis* (adjacent) or *trans* (opposite) to a phosphonate group, resulting in two different isomers with distinct ^31^P NMR signals and an area ratio reflecting their relative abundance. The ^17^O NMR signal associated with the ternary species appears upfield (*δ*_O_ 1116 ppm, at −5 °C) relative to the 1:2 main species (*δ*_O_ 1124 ppm at −5 °C, in the barycenter of the *syn*/*anti* fast exchange averaged signal; cf. Figure 7B, pH 9.3, and also see Figure S29B, SI). This shift is attributed to the coordination of an additional hard Lewis-base anionic ligand to the U(VI) center, consistent with the ^17^O NMR signal of UO_2_(OH)_4_^2−^ observed at *δ*_O_ 1111 ppm at −5 °C [62]. Notably, the absence of ^17^O NMR signals in the 1105–1095 ppm region excludes the presence of uranyl carbonate species, which would otherwise exhibit sharp and well observable resonances [63].

Furthermore, ^1^H and ^13^C NMR spectra also exhibit a distinct set of signals arising from the ternary species (*t*), being consistent with the structural features established for the 1:2 U(VI)-PBTC complex species (*c*). Not only is the doublet of doublets due to *t*1a also well separated, but it is also even more downfield shifted compared to *c*1a and *l*1a, as is the case for all other corresponding ^1^H signals in the three PBTC environments (*t*, *c*, and *l*); cf. Figure 1A and Figure S27B, SI for ^1^H assignment. Among the carboxyl carbon signals associated with the ternary species, C2′ is even further downfield than that of the 1:2 complex species (cf. Figure 3, Figures S28 and S29, SI).

It is important to note that the ternary species observed by NMR is different from the binary species observed by UV-Vis at high pH (such as component 8, cf. Section 2.1 and Section 2.3). Even at high pH, the UV-Vis spectral signatures are typical for binary species, that is, vibrational-coupled, structured absorption spectra (Figure S31, SI). By contrast, U(VI) hydroxo species (hydrolysis and ternary species) reveal broad, rather unstructured features, accompanied by notable changes in molar absorptivity as seen for, e.g., in U(VI) citrate complexes [33]. As seen from Table S8, SI, the molar absorptivity is virtually identical (~28 M^−1^ cm^−1^) for the last two species predominating at high pH. Given NMR’s low sensitivity in terms of concentration, the much higher U(VI) concentration used for NMR than those used for other spectroscopic techniques probably provoked the formation of a ternary species in the first place, and was thus not observed by the other methods due to the lower U(VI) concentration used. In contrast, NMR’s high sensitivity in terms of molecular structure, the comprehensibility of coordination-induced changes in the local electronic environment of nuclei allows for a reasonable assignment to a ternary species (see Figure 7 and Figures S26–S29, SI).

## 3. Materials and Methods

### 3.1. General Remarks

All preparation steps were performed with safety precautions according to both radio- and chemotoxicity of uranium. All chemicals were used without further purification. The samples have been covered with aluminum foil to protect them from alterations due to the influence of light.

### 3.2. Preparation of Aqueous Sample Solutions

For the analyses, PBTC (TCI Germany GmbH, 50% in water, >90%) and either uranyl(VI) nitrate or uranyl(VI) chloride (Merck, p.a.) were used, depending on the requirements of the analytical method. Stock solutions of the individual components were prepared separately and then combined in the required molar ratios. All preparations were carried out with deionized water (18.2 MΩ cm; Millipore, Taufkirchen, Germany). An ionic strength of *I*_m_ = 0.5 m NaCl (Merck, p.a.) was employed as a background electrolyte for the complex speciation studies.

The pH values of the sample solutions were adjusted using a pH meter (inoLab pH 730) equipped with a pH electrode (Schott, BlueLine, SI Analytics, Mainz, Germany) and either HCl or NaOH of various concentrations, unless stated otherwise. For sample solutions with a maximum ionic strength of 0.5 m NaCl, no pH correction was required, as the experimental pH (pH_exp_) and the corrected pH (pH_corr_) differed by only 0.05, which is within the measurement uncertainty. The pH meter was calibrated using a three-point calibration procedure, selecting the appropriate among buffer solutions at pH 1.679, 4.006, 6.865, 9.180, and 12.47 (standard DIN/NIST buffer solutions, WTW).

The concentrations of U, P, and Na in stock and sample solutions were verified by ICP-MS (Elan 9000 PerkinElmer, Shelton, CT, USA). Furthermore, the composition and purity of the PBTC stock solution were regularly checked by NMR spectroscopy as described in reference [32].

For NMR spectroscopy, stock solutions 0.5 M in uranyl nitrate were prepared by accurately weighing the solid and dissolving it in deionized H_2_O or D_2_O (99.98% D, Deutero, Kastellaun, Germany). PBTC stock solutions in H_2_O were prepared by dilution of aliquots of the original PBTC batch. For PBTC stock solutions in D_2_O, a concentrated aqueous PBTC solution was lyophilized, and the resulting pale yellow glassy material was re-dissolved in D_2_O; the concentration was determined by ^31^P q-NMR spectroscopy. Solutions in pure D_2_O were prepared only for ^1^H NMR measurements of diluted samples, whereas ^13^C, ^31^P, and ^17^O NMR measurements were performed in solutions containing 10% D_2_O, sufficient to facilitate spectrometer deuterium lock. Using these stock solutions, sample series were prepared with varying absolute analyte concentrations (U(VI) and PBTC), U(VI):PBTC molar ratios, and pH values by diluting the required amounts and adjusting the pH. Adjustments were made using HCl and NaOH (p.a.) of various concentrations, as well as NaOD (40% in D_2_O with 99% D, Deutero, Germany) and DCl (37% in D_2_O with 99% D, Deutero, Germany). For solutions in pure D_2_O, pH readings were corrected to pD using pD = pH_read_ + 0.4. For solutions containing 10% D_2_O, an additional correction of +0.04 pH units was applied, reflecting the nearly linear dependence of the pH meter reading on atom-% deuterium [64].

### 3.3. Nuclear Magnetic Resonance (NMR) Spectroscopy

Except for some dedicated measurements (see below), NMR spectra were recorded with an Agilent DD2-600 NMR system, operating at 14.1 T with corresponding ^1^H, ^31^P, ^23^Na, ^13^C, and ^17^O resonance frequencies of 599.8, 242.9, 158.7, 150.8, and 81.4 MHz, respectively, using a 5 mm one NMR^TM^ probe. Unless stated otherwise, spectra were acquired at (25 ± 0.2) °C. Temperature stability in variable temperature (VT) measurements was achieved by gas flow heating the tube by the probe-internal heating coil or cooling the sample at the desired temperature upon combination with an external cooling unit. After changing the set temperature, samples were allowed to equilibrate for at least 15 min, and constancy was verified by deuterium lock stability.

^1^H NMR spectra were obtained after a 2 s pre-saturation pulse with offset on the water resonance to suppress the water signal, followed by a π/6 (2.6 µs) excitation pulse, 2 s acquisition time (at) and 2 s relaxation delay (d1), accumulating and averaging a varying number of individual scans (nt, between 32 and 256), depending on the sample concentration. Regularly, ^1^H spectra were acquired without heteronuclear decoupling. Occasionally, ^1^H spectra were ^31^P broadband decoupled, denoted as ^1^H{^31^P}. ^31^P (^13^C{^1^H}) spectra were obtained after applying 4.2 µs (2.8 µs) π/6 excitation pulses, at = 2 s (1 s) and d1 = 5 s (5 s), and with nt ranging from 32 to 1024 (8k to 64k), using ^1^H inverse-gated decoupling (^1^H broadband decoupling) during FID acquisition, respectively. ^17^O NMR spectra of samples containing uranyl oxygens enriched in ^17^O (~30% enrichment) were acquired using a 23 µs π/2 excitation pulse, at = 50 ms and d1 = 200 ms, and with nt ranging between 4k and 64k, depending on sample conditions. In the case of ^17^O measurement at natural isotope abundance (0.038%), a 10 mm tube and a corresponding 10 mm low-gamma broadband direct detection probe were used, applying the following parameters at = 150 ms, d1 = 350 ms, nt = 640k. The FID was shifted by −8 data points in order to cut residual high-amplitude interference from acoustic ringing, helping to reduce the otherwise severe baseline perturbations and phasing issues. Quantification of PBTC stock solution aliquots was achieved by increasing d1 to 90 s (^31^P q-NMR), using weighted amounts of NaH_2_PO_4_ as an internal standard.

Heteronuclear single-quantum coherence (HSQC) and heteronuclear multiple-bond correlation (HMBC) were accomplished using pulse sequences taking advantage of gradient-selection and adiabatic pulses. All 2D correlation spectra were acquired using a 1 s pre-saturation pulse for HDO signal suppression. ^1^H,^13^C-HSQC, ^1^H,^13^C-HMBC, and ^1^H,^31^P-HMBC spectra were acquired with 2k × 512, 2k × 1k, and 2k × 256 complex points in *F*_2_ and *F*_1_, accumulating 32, 64, and 24 transitions per *F*_1_ increment, with a d1 of 1 s, respectively. For polarization transfer, (2 × *J*)^−1^ delays of 4.0 and 62.5 ms were chosen, corresponding to 125 Hz ^1^*J*(H,C) in HSQC and 8 Hz ^n^*J*(H,C/P) in HMBC, respectively. Homonuclear gradient-selected correlation spectroscopy (gCOSY) and the phase-sensitive nuclear Overhauser effect spectroscopy (NOESY, mixing time 500 ms) data were obtained applying 2k × 256 (*F*_2_ × *F*_1_) complex points, 16 and 64 transitions per *F*_1_ increment, with a d1 of 1 s, respectively.

Field-dependent ^31^P NMR measurements were also carried out on a 9.4 T Agilent 400-MR system, with corresponding ^1^H and ^31^P resonance frequencies of 399.8 and 161.9 MHz, respectively.

^1^H and ^13^C NMR spectra were referenced relative to the methyl signal of TMSP-*d*_4_ in D_2_O with *δ*_H_ and *δ*_C_ set to 0.00 ppm. ^31^P NMR spectra were referenced to external H_3_PO_4_ with *δ*_P_ = 0.0 ppm. For ^17^O NMR, the bulk water signal was used as a reference, *δ*_O_ = 0 ppm.

^17^O isotopic enrichment in uranyl oxygens was achieved by irradiating 1 mL of a 1.017 M uranyl nitrate solution prepared in H_2_^17^O (90% ^17^O, Sigma-Aldrich, St. Louis, MO, USA) at 370 nm in a 1 cm quartz cuvette overnight [65,66].

### 3.4. Attenuated Total Reflection Fourier-Transform Infrared (ATR FT-IR) Spectroscopy

Analyses were performed using a Vertex 80/v vacuum FT-IR spectrometer (Bruker Optics Inc., Billerica, MA, USA) equipped with a mercury cadmium telluride detector. For the measurements, an attenuated total reflection (ATR) unit (DURA SamplIR II, Smiths Inc., Dothan, AL, USA) with a horizontal diamond with nine internal reflections on the upper surface and an incidence angle of 45° was used. A flow cell with a volume of 200 µL was mounted to ensure an adequate background subtraction without external thermal interferences. Spectra were recorded in the range of 1800 to 800 cm^−1^ at a spectral resolution of 4 cm^−1^ under ambient atmosphere.

First, the spectral properties of the pure ligand were investigated and used as a blank at distinct pH values in the range from 2 to 10. Afterwards, the complexation of U(VI)-chloride and PBTC was studied at fixed metal to ligand ratios of 1:1 (5 mM U(VI):5 mM PBTC) and 1:5 (5 mM U(VI):25 mM PBTC), with *I*_m_ = 0.5 m NaCl at the same pH range. For background subtraction, an aqueous NaCl solution with the same ionic strength was used.

### 3.5. UV-Vis Spectroscopy

The UV-Vis measurements were performed using the multichannel spectrometer MCS UV-NIR 601 (Carl Zeiss, Jena, Germany), which consists of a 1024 silicon photodiode array detector and operates in the wavelength range from 190 to 1000 nm with a pixel dispersion λpixel of 0.8 nm/pixel, and thus has a spectral resolution of 2.4 nm (by Rayleigh criterion ΔλRayleigh = 3Δ∙λpixel). Via fiber optics, a variable path length cuvette holder (Avantes) was connected with the spectrometer and the deuterium-halogen light source (DH-2000, Avantes, Eerbeek, Netherlands). Absorption spectra were recorded in the wavelength range between 450 and 650 nm using a macro cuvette with a 50 mm optical path length and two openings, operated as continuous pH- and ligand titration measurements. Two sets of experiments were performed: first, ligand titration at constant pH~2, and second, pH titrations with constant U(VI) to PBTC ratio. For the ligand titration, 7 mL of a solution containing 0.5, 1, or 10 mM U(VI) at pH 2 and an ionic strength *I*_m_ of 0.5 m NaCl was prepared. Aliquots of a 125 mM PBTC stock solution (adjusted to the same pH and ionic strength) were gradually added until the target U(VI):PBTC ratios were reached, namely 1:5 and 1:10 for the 0.5 and 1 mM U(VI) solutions, and 1:3.5 for the 10 mM U(VI) solution. Subsequently, pH titration was carried out up to pH 10 by gradually adding small amounts of 1 M or 5 M NaOH, resulting in increments of 0.2 to 0.5 pH units. After each addition of NaOH, the sample was equilibrated upon stirring for at least 3 to 5 min to achieve pH stability. The recorded absorption spectra were baselinecorrected by using OriginLab (OriginPro, version 2020b, OriginLab Corporation, Northampton, MA, USA). The number of components and their distribution were determined using ITFA software, version V1-4, which is described in detail in [29] and applied in [29,55,67,68,69].

From different UV-Vis series, the complexation constants were determined using the multivariate factor analysis programs SPECFIT/32 [70] and HypSpec (Vers. 1.1.18, Protonic Software Leeds, UK [71]), with SPECFIT/32 being the primary tool used. More details about the software are described in Guo et al. [72] and Gampp [73,74,75], and several examples of successful applications of SPECFIT/32 are highlighted in references [33,46,76,77,78]. To evaluate the quality of the fits, the Durbin–Watson statistic was analyzed in combination with the relative error of fit. A fit was considered acceptable if the Durbin–Watson factor was within the range 1.5 < DW < 2.4, indicating no autocorrelation in the residuals and the correctness of the chosen model [79]. Additionally, the relative error of fit (REF) was required to be below 2%.

### 3.6. Raman Spectroscopy

Raman investigations of PBTC with U(VI) were performed using a DRX SmartRaman (ThermoFisher Scientific, Dreieich, Germany) equipped with a 780 nm excitation laser with 14 mW laser energy and a grating of 830 lines per mm, resulting in a resolution of 3–4 cm^−1^ at an aperture of the slit of 50 µm. The spectra were recorded in the range of 1150–600 cm^−1^ with a 10 mm quartz cuvette in the automatic fluorescence correction mode.

All Raman spectra were baseline-corrected and represent the difference between spectra obtained from U(VI)-containing and U(VI)-free solutions of the same PBTC contents and pH. The resulting Raman difference spectra were normalized according to the area of the symmetric N-O stretching vibration of the NO_3_^−^ ion at 1048 cm^−1^. The Raman signals of the uranyl and nitrate ions were fitted by a Lorentzian fit using the Origin Peak Fitting Module (OriginPro 2020b, OriginLab Corporation, USA).

### 3.7. Time-Resolved Laser-Induced Luminescence Spectroscopy (TRLFS)

The TRLFS measurements were performed using a pulsed Nd:YAG laser (10 Hz, Quanta Ray, Spectra Physics, Santa Clara, CA, USA) combined with an OPO (GWU-Lasertechnik, Germany) set to 265 nm. The laser energy was tuned to an average of 7 mJ. A spectrograph (Shamrock 303i, Andor Technology, Oxford Instruments, Oxfordshire, UK) equipped with a 150 lines/mm grating (blaze: 500 nm) with a slit width of 150 µm and an ICCD camera (iStar DH720-18V-73, Andor Technology, Oxford Instruments, UK) were used to detect the emitted light in the spectral range between 285 and 855 nm. Time-resolved emission spectra were recorded with an initial delay of 500 ns at a gate width of 10 ms. To collect the emission kinetics, emission spectra (each accumulated for 100 laser pulses) were measured with an increasing delay time (gate step 40 µs, 100 steps). To minimize photodegradation of the U(VI)-PBTC complexes, TRLFS measurements were carried out under cryogenic conditions (*T* = 4 K) using a closed-cycle liquid helium cryostat (CKW-21, Sumitomo Heavy Industries Ltd., Tokyo, Japan). To semi-quantify the extent of photodegradation under cryogenic conditions, the irradiation by short laser pulses, a series of emission spectra of the time equivalent to the TRLFS kinetics measurements, was collected. For two distinct U(VI):PBTC ratios at pH = 2, we calculate a decrease of approximately 15%. Two distinct sets of samples were analyzed, both with a fixed uranyl concentration of 10 mM: one with varying uranyl-to-PBTC ratios up to 1:5 at pH 2, and the other with a constant uranyl-to-PBTC ratio of 1:5 and a pH range extending up to 9. TRLFS data were analyzed with parallel factor analysis (PARAFAC) as described by Drobot et al. [80] using MATLAB 2020b software (The Mathworks Corporation, Natick, MA, USA) and Origin (OriginPro 2020b, OriginLab Corporation, USA).

### 3.8. Quantum Chemistry Calculations Based on the Density Functional Theory (DFT)

Quantum chemistry calculations based on the density functional theory using the PBE exchange–correlation functional [81] were performed with the Turbomole software suite (version 6.6) [82]. Triple zeta quality basis sets with polarization functions, def-TZVP, were applied for all atoms. For U, the Stuttgart–Dresden small core effective core potential (ECP) was used to account for core electrons and related relativistic effects [83]. The electronic structure calculations were spin-restricted, considering the closed-shell nature of the U(VI) ion in the uranyl complexes. In addition, the resolution of identity (RI) approximation was employed for the electronic Coulomb interaction to speed up the self-consistent field process during geometry optimizations, without significantly sacrificing accuracy. In the gas phase, a vibrational normal mode analysis was performed, and corresponding thermodynamic corrections were applied to the solution energy to derive Gibbs free energies in water for each species based on a thermodynamic cycle. Also, in solution, a normal mode analysis was performed to confirm the energy minima on the potential energy surface. In several cases, 1–3 small imaginary frequencies below 100 cm^−1^ were obtained, corresponding to strongly delocalized vibrational modes. Attempts to overcome these modes resulted in energy gains of up to 1 kJ/mol, demonstrating the boundary of geometry optimization accuracy of the rather flexible functional groups in the PBTC ligands. While explicit water molecules in the complexes accounted for short-range solvation effects, the COSMO [84] variant of the polarizable continuum solvation model [85] was used for long-range solvation effects with a dielectric constant of 78.4 and default parametrization for water as implemented in Turbomole. All studied structures were generated from preoptimized neutral PBTC and the uranyl dication aquo complex using a systematic approach. Initially, the PBTC ligand and the uranyl aquo complex were individually optimized to ensure their minimal energy configurations. For PBTC, this involved a screening of various intramolecular hydrogen bonding patterns. In contrast, for the rigid UO_2_^2+^ linear molecule, we checked the number of explicit aqua ligands in the equatorial plane, having uranium in the center and uranyl’s oxygens in the axial positions. As a result, the pentaaquo-uranyl, [UO_2_(H_2_O)_5_]^2+^, was retained and, together with PBTC, served as the building blocks for the subsequent assembly of the studied complexes. Different coordination modes of PBTC were checked, all of them involving a coordinated phosphonate oxygen, which was shown to be the first to deprotonate in water and thus a primary coordination target.

## 4. Conclusions

This study presents the first comprehensive investigation of the formation of complexes between the uranyl(VI) ion (UO_2_^2+^) and 2-phosphonobutane-1,2,4-tricarboxylic acid (PBTC) in aqueous solution over a broad pH range (2–11). PBTC is a common cement additive and might affect the mobility of radionuclides by binding them. Using a combination of spectroscopic techniques (NMR, Raman, ATR FT-IR, UV-Vis, and TRLFS) and quantum chemical DF calculations, we determined speciation and structures of the resulting complexes. In solutions with PBTC excess, uranyl(VI) predominantly forms stable 1:2 chelate complexes. In these complexes, each PBTC ligand coordinates via its phosphonate and the *geminal* C2′ carboxylate group, resulting in the formation of a stable six-membered chelate ring. This binding motif persists over a very wide pH range, exceptions occurring only under strongly acidic conditions and with low ligand excess, where a 1:1 complex forms, while 1:3 complexes are excluded by both calculation and experiment. NMR and ATR FT-IR data, supported by DF calculations, confirm the invariance of this chelate structure regardless of the ligand’s protonation state. There are multiple isomers of the 1:2 complex, which differ in their *syn*/*anti* and *cis*/*trans* ligand arrangements and dynamically interconvert. This formal and effective 1:2 “main species” has a high stability constant (log *K* ≈ 9.7 ± 1) and a formation free energy of approximately –55.5 kJ/mol, with no significant further stabilization upon additional deprotonation. As indicated by NMR, a ternary U(VI)-PBTC-hydroxo species only forms at high uranium concentrations and very high pH. Overall, excess PBTC prevents uranyl(VI) hydrolysis and precipitation, even in high millimolar concentrations and under alkaline conditions, inhibiting polynuclear or hydroxo complex formation. In summary, PBTC is demonstrated to be a highly effective chelator of uranyl(VI) over a broad pH range, forming stable 1:2 chelate complexes. These findings could be relevant for long-term safety assessments of nuclear waste repositories, as PBTC may influence the mobility of uranium if it is released from cementitious materials in relevant concentrations in such environments. To simulate the effect of PBTC under more realistic repository conditions, U(VI) retention by cementitious materials in cement pore waters containing potentially competing ions such as Ca^2+^, Mg^2+^, and Al^3+^ is currently being investigated in the absence and presence of PBTC.

## Figures and Tables

**Table 1:** Species-associated emission band maxima obtained from PARAFAC analyses of the two titration series.

Species	Main Peak Position/nm				
	P1	P2	P3	P4	P5
UO_2_^2+^ *^a^*	486	507	530	556	(584)
UO_2_^2+^ from [36]	484	506	529		
	*concentration series*				
1:1 U(VI)-PBTC species	500	522	545	570	(599)
1:2 U(VI)-PBTC species	495	516	540	564	(593)
	*pH series*				
1:2 U(VI)-PBTC species	496	517	540	565	(593)
Residual spectral component	492	513	535	559	(587)

*^a^* The spectrum of the uranyl aquo ion was used as a fixed input parameter.

**Table 2:** Relative Gibbs free energies of formation, in kJ/mol, of the neutral 1:1 U(VI):PBTC complex UO_2_(H_3_PBTC)(H_2_O)_n_, according to Equation (2), calculated for varying coordination modes (CMs) and uranium coordination numbers (CNs).

CM	CN	∆*G*_rel_
mono	5	36.9
	4	75.6
	3	86.9
bi	5	42.6
	4	53.7
	3	92.1
chel	5	0.0
	4	2.0
	3	39.6

**Table 4:** Complex formation constants of U(VI)-PBTC species at *I_m_* = 0.5 m NaCl, *T* = (25 ± 1) °C, errors 2σ.

Species	U(VI)-Ligand- Proton (xyz)	$\log\;\beta_{xyz}$	log *K*	Δ*G* in kJ/mol
UO_2_(H_3_PBTC)^0^	113	25.5 ± 1.0	4.8 ± 0.2	−27
UO_2_(H_3_PBTC)_2_^2−^	126	51.0 ± 0.7	9.7 ± 0.1	−55
UO_2_(H_3_PBTC)(H_2_PBTC)^3−^	125	47.1 ± 0.7	10.5 ± 0.2	−60
UO_2_(H_2_PBTC)_2_^4−^	124	41.6 ± 0.8	9.8 ± 0.2	−56
UO_2_(H_2_PBTC)(HPBTC)^5−^	123	35.1 ± 0.9	9.3 ± 0.3	−53
UO_2_(HPBTC)_2_^6−^	122	28.5 ± 1.5	8.9 ± 0.5	−51
UO_2_(HPBTC)(PBTC)^7−^	121	19.9 ± 1.5	10.2 ± 0.7	−58

## Data Availability

The original contributions presented in this study are included in the article/Supplementary Material. Further inquiries can be directed to the corresponding author(s).

## Supplementary Materials

The following supporting information can be downloaded at: https://www.mdpi.com/article/10.3390/molecules30204144/s1, Figure S1: Raman spectra obtained at different U(VI):PBTC ratios; Figure S2: Example of luminescence spectra and the correlation of the determined ν_1_(UO_2_) with those calculated from the luminescence spectra; Figure S3: Exemplary UV-Vis spectra for varying PBTC concentrations up to 35 mM; Figure S4: Abstract spectra of the first 12 components from ITFA; Figure S5: Factor loading from VARIMAX rotation for concentration and pH series; Figure S6: Luminescence spectra of U(VI)–PBTC complexes at pH = 2 for two different U(VI):PBTC ratios; Figure S7: Species-associated luminescence spectra of the presumed U(VI)-PBTC 1:2 complex obtained from PARAFAC analyses; Figure S8: Assignment of ATR FT-IR bands of PBTC at pH 2, 5, and 8; Figure S9: ATR FT-IR spectra of 25 mM PBTC, U(VI) + PBTC solution and the corresponding difference spectra of both; Figures S10–S30: Additional various ^1^H, ^13^C, ^31^P, and ^17^O NMR spectra of selected samples. Where relevant, spectra are accompanied by brief annotations highlighting characteristic resonances, signal assignments, or changes in chemical shifts discussed in the main text; Figure S31: Generic structures of the six possible ligand arrangements occurring for racemic PBTC in 1:2 U(VI)-PBTC complexes; Figure S32: Extracted single-component UV-Vis spectra of the individual (UO2)_x_(PBTC)_y_(H)_z_ complex species; Table S1: Results of Raman studies: Peak position of Raman active symmetric stretching vibration mode ν_1_(UO_2_) of U(VI)-PBTC samples for the concentration series at pH 2; Table S2: Characteristics of the pH dependent luminescence spectra; Table S3: Example of statistical results of the ITFA of the UV-Vis spectra; Table S4: Assignment of the infrared absorption bands of PBTC and of some selected compounds for comparison; Table S5: Relative Gibbs free energies of various isomers of UO_2_(HPBTC)_2_^6−^; Table S6: Ratio of calculated to measured p*K*_a_ values; Table S7: Estimated lowering of p*K*_a_ values of PBTC ligands in U(VI) complexes; Table S8: Wavelength and corresponding maximum extinction determined from the deconvoluted single-component UV-Vis spectra of the U(VI)-PBTC complex species. All references cited in the supporting information file are included as references of the manuscript.

## References

[1] Saedi A., Jamshidi-Zanjani A., Darban A.K. (2021). A review of additives used in the cemented paste tailings: Environmental aspects and application. J. Environ. Manag..

[2] Chen J., Jia J., Zhu M. (2024). A critical review of the effect of chemical organic admixtures for OPC-based materials. Mater. Chem. Phys..

[3] Demadis K.D., Lykoudis P. (2005). Chemistry of Organophosphonate Scale Growth lnhibitors: 3. Physicochemical Aspects of 2-Phosphonobutane-1,2,4-tricarboxylate (PBTC) And Its Effect on CaCO_3_ Crystal Growth. Bioinorg. Chem. Appl..

[4] Demadis K.D., Lykoudis P., Raptis R.G., Mezei G. (2006). Phosphonopolycarboxylates as chemical additives for calcite scale dissolution and metallic corrosion inhibition based on a calcium-phosphonotricarboxylate organic−inorganic hybrid. Cryst. Growth Des..

[5] Rott E., Happel O., Armbruster D., Minke R. (2020). Behavior of PBTC, HEDP, and Aminophosphonates in the Process of Wastewater Treatment. Water.

[6] Rott E., Steinmetz H., Metzger J.W. (2018). Organophosphonates: A review on environmental relevance, biodegradability and removal in wastewater treatment plants. Sci. Total Environ..

[7] Qiao Z., Fan W., Zhang Y., Fu X., Yang H., Zhang F. (2025). Enhancement of Magnesium Oxysulfate Cement by Acid Modifiers and Its Reaction Mechanism. Materials.

[8] Heal K., Smith K., Younger P., McHaffie H., Batty L. (2004). Phosphorus in Environmental Technology: Principles and Applications.

[9] Armbruster D., Müller U., Happel O. (2019). Characterization of phosphonate-based antiscalants used in drinking water treatment plants by anion-exchange chromatography coupled to electrospray ionization time-of-flight mass spectrometry and inductively coupled plasma mass spectrometry. J. Chromatogr. A.

[10] Rickert J., Thielen G. (2004). Influence of a long-term retarder on the hydration of clinker and cement. Cem. Concr. Aggreg..

[11] Salvadó V., Escoda M.L.s., de la Torre F. (1999). A study of the complex formation between trivalent ions (Al^3+^, Fe^3+^) and 2-phosphonobutane-1,2,4-tricarboxylic acid and their industrial applications. Polyhedron.

[12] Clearfield A., Demadis K. (2012). Metal Phosphonate Chemistry: From Synthesis to Applications.

[13] Huang Y., Zheng H., Li H., Zhao C., Zhao R., Li S. (2020). Highly selective uranium adsorption on 2-phosphonobutane-1,2,4-tricarboxylic acid-decorated chitosan-coated magnetic silica nanoparticles. Chem. Eng. J..

[14] Spinthaki A., Matheis J., Hater W., Demadis K.D. (2018). Antiscalant-driven inhibition and stabilization of “magnesium silicate” under geothermal stresses: The role of magnesium–phosphonate coordination chemistry. Energy Fuels.

[15] Knepper T.P. (2003). Synthetic chelating agents and compounds exhibiting complexing properties in the aquatic environment. TrAC Trends Anal. Chem..

[16] Srivastava A., Dumpala R.M.R., Kumar P., Kumar R., Rawat N. (2022). Chemical and redox speciation of uranyl with three environmentally relevant bifunctional chelates: Multi-technique approach combined with theoretical estimations. Inorg. Chem..

[17] Szabó Z. (2002). Structure, equilibrium and ligand exchange dynamics in the binary and ternary dioxouranium(VI)–glyphosate–fluoride system. A multinuclear NMR study. J. Chem. Soc. Dalton Trans..

[18] Nguyen Trung C., Begun G., Palmer D.A. (1992). Aqueous uranium complexes. 2. Raman spectroscopic study of the complex formation of the dioxouranium(VI) ion with a variety of inorganic and organic ligands. Inorg. Chem..

[19] Nguyen-Trung C., Palmer D., Begun G., Peiffert C., Mesmer R. (2000). Aqueous uranyl complexes 1. Raman spectroscopic study of the hydrolysis of uranyl(VI) in solutions of trifluoromethanesulfonic acid and/or tetramethylammonium hydroxide at 25 C and 0.1 MPa. J. Solut. Chem..

[20] Lu G., Forbes T.Z., Haes A.J. (2016). Evaluating best practices in Raman spectral analysis for uranium speciation and relative abundance in aqueous solutions. Anal. Chem..

[21] Tsushima S. (2011). On the “yl” bond weakening in uranyl(VI) coordination complexes. Dalton Trans..

[22] Lu G., Haes A.J., Forbes T.Z. (2018). Detection and identification of solids, surfaces, and solutions of uranium using vibrational spectroscopy. Coord. Chem. Rev..

[23] Yang Y., Liu Q., Lan Y., Zhang Q., Zhu L., Yang S., Tian G., Cao X., Dolg M. (2024). Systematic Raman Spectroscopic Study of the Complexation of Uranyl with Fluoride. Phys. Chem. Chem. Phys..

[24] Kirkham A.J., Bryan N.D., May I. (2006). Uranyl Coordination to Environmentally Relevant Polyhydroxy Carboxylate Ligands. Recent Advances in Actinide Science.

[25] Birjkumar K.H., Bryan N.D., Kaltsoyannis N. (2011). Computational investigation of the speciation of uranyl gluconate complexes in aqueous solution. Dalton Trans..

[26] Faulques E., Kalashnyk N., Massuyeau F., Perry D. (2015). Spectroscopic markers for uranium(VI) phosphates: A vibronic study. RSC Adv..

[27] Drobot B., Bauer A., Steudtner R., Tsushima S., Bok F., Patzschke M., Raff J., Brendler V. (2016). Speciation studies of metals in trace concentrations: The mononuclear uranyl(VI) hydroxo complexes. Anal. Chem..

[28] Meinrath G. (1997). Uranium(VI) speciation by spectroscopy. J. Radioanal. Nucl. Chem..

[29] Rossberg A., Reich T., Bernhard G. (2003). Complexation of uranium(VI) with protocatechuic acid—Application of iterative transformation factor analysis to EXAFS spectroscopy. Anal. Bioanal. Chem..

[30] Malinowski E.R., Howery D.G. (2002). Factor Analysis in Chemistry.

[31] Kaiser H.F. (1958). The varimax criterion for analytic rotation in factor analysis. Psychometrika.

[32] Kretzschmar J., Wollenberg A., Tsushima S., Schmeide K., Acker M. (2022). 2-Phosphonobutane-1,2,4,-Tricarboxylic Acid (PBTC): pH-Dependent Behavior Studied by Means of Multinuclear NMR Spectroscopy. Molecules.

[33] Kretzschmar J., Tsushima S., Lucks C., Jäckel E., Meyer R., Steudtner R., Müller K., Rossberg A., Schmeide K., Brendler V. (2021). Dimeric and trimeric uranyl(VI)–citrate complexes in aqueous solution. Inorg. Chem..

[34] Geipel G. (2006). Some aspects of actinide speciation by laser-induced spectroscopy. Coord. Chem. Rev..

[35] Kato Y., Meinrath G., Kimura T., Yoshida Z. (1994). A study of U (VI) hydrolysis and carbonate complexation by time-resolved laser-induced fluorescence spectroscopy (TRLFS). Radiochim. Acta.

[36] Demnitz M., Hilpmann S., Lösch H., Bok F., Steudtner R., Patzschke M., Stumpf T., Huittinen N. (2020). Temperature-dependent luminescence spectroscopic investigations of uranyl(VI) complexation with the halides F^−^ and Cl^−^. Dalton Trans..

[37] Shang C., Reiller P.E. (2020). Determination of formation constants and specific ion interaction coefficients for Ca_n_UO_2_(CO_3_)_3_^(4− 2n)−^ complexes in NaCl solution by time-resolved laser-induced luminescence spectroscopy. Dalton Trans..

[38] Sirven J.-B., Szenknect S., Vors E., Anzalone E., Benarib S., Sarr P.-M., Reiller P.E., Mesbah A., Dacheux N., Vercouter T. (2023). Time-resolved laser-induced fluorescence spectroscopy and chemometrics for fast identification of U(VI)-bearing minerals in a mining context. Spectrochim. Acta Part A Mol. Biomol. Spectrosc..

[39] Billard I., Ansoborlo E., Apperson K., Arpigny S., Azenha M.E., Birch D., Bros P., Burrows H.D., Choppin G., Couston L. (2003). Aqueous Solutions of Uranium(VI) as Studied by Time-Resolved Emission Spectroscopy: A Round-Robin Test. Appl. Spectrosc..

[40] Oher H., Ferru G., Couston L., Berthon L., Guillaumont D., Réal F., Vercouter T., Vallet V. (2021). Influence of the first coordination of uranyl on its luminescence properties: A study of uranyl binitrate with N, N-dialkyl amide DEHiBA and water. Inorg. Chem..

[41] Burrows H., Kemp T. (1974). The photochemistry of the uranyl ion. Chem. Soc. Rev..

[42] Kim Y., Marcano M.C., Ellis B.R., Becker U. (2018). Photocatalytic reduction of uranyl: Effects of organic ligands and UV light wavelengths. Am. J. Sci..

[43] Wang Z., Zachara J.M., Yantasee W., Gassman P.L., Liu C., Joly A.G. (2004). Cryogenic laser induced fluorescence characterization of U(VI) in Hanford vadose zone pore waters. Environ. Sci. Technol..

[44] Marcantonatos M.D., Pawlowska M.M. (1989). The second emission of the uranyl ion in aqueous solution. J. Chem. Soc. Faraday Trans. 1 Phys. Chem. Condens. Phases.

[45] Choppin G.R., Rao L.F. (1984). Complexation of pentavalent and hexavalent actinides by fluoride. Radiochim. Acta.

[46] Heller A., Senwitz C., Foerstendorf H., Tsushima S., Holtmann L., Drobot B., Kretzschmar J. (2023). Europium(III) meets etidronic acid (HEDP): A coordination study combining spectroscopic, spectrometric, and quantum chemical methods. Molecules.

[47] Pasilis S.P., Pemberton J.E. (2003). Speciation and coordination chemistry of uranyl (VI)− citrate complexes in aqueous solution. Inorg. Chem..

[48] Zenobi M.C., Luengo C.V., Avena M.J., Rueda E.H. (2008). An ATR-FTIR study of different phosphonic acids in aqueous solution. Spectrochim. Acta A Mol. Biomol. Spectrosc..

[49] Zou C.J., Tang Q.W., Lan G.H., Tian Q., Wang T.Y. (2012). Enhancement inhibition efficiency of PBTCA depending on the inclusion complex with hydroxypropyl-β-cyclodextrin. J. Incl. Phenom. Macrocycl. Chem..

[50] Barkleit A., Foerstendorf H., Li B., Rossberg A., Moll H., Bernhard G. (2011). Coordination of uranium(VI) with functional groups of bacterial lipopolysaccharide studied by EXAFS and FT-IR spectroscopy. Dalton Trans..

[51] Kakihana M., Nagumo T., Okamoto M., Kakihana H. (1987). Coordination structures for uranyl carboxylate complexes in aqueous solution studied by IR and carbon-13 NMR spectra. J. Phys. Chem..

[52] Deacon G., Phillips R. (1980). Relationships between the carbon-oxygen stretching frequencies of carboxylato complexes and the type of carboxylate coordination. Coord. Chem. Rev..

[53] Kretzschmar J., Strobel A., Haubitz T., Drobot B., Steudtner R., Barkleit A., Brendler V., Stumpf T. (2020). Uranium(VI) complexes of glutathione disulfide forming in aqueous solution. Inorg. Chem..

[54] Müller K., Brendler V., Foerstendorf H. (2008). Aqueous uranium(VI) hydrolysis species characterized by attenuated total reflection Fourier-transform infrared spectroscopy. Inorg. Chem..

[55] Lucks C., Rossberg A., Tsushima S., Foerstendorf H., Scheinost A.C., Bernhard G. (2012). Aqueous uranium(VI) complexes with acetic and succinic acid: Speciation and structure revisited. Inorg. Chem..

[56] Kretzschmar J., Tsushima S., Drobot B., Steudtner R., Schmeide K., Stumpf T. (2020). Trimeric uranyl(VI)–citrate forms Na^+^, Ca^2+^, and La^3+^ sandwich complexes in aqueous solution. Chem. Commun..

[57] Alsobrook A.N., Alekseev E.V., Depmeier W., Albrecht-Schmitt T.E. (2011). Incorporation of Mn(II) and Fe(II) into Uranyl Carboxyphosphonates. Cryst. Growth Des..

[58] Altmaier M., Yalçıntaş E., Gaona X., Neck V., Müller R., Schlieker M., Fanghänel T. (2017). Solubility of U(VI) in chloride solutions. I. The stable oxides/hydroxides in NaCl systems, solubility products, hydrolysis constants and SIT coefficients. J. Chem. Thermodyn..

[59] Migdisov A.A., Boukhalfa H., Timofeev A., Runde W., Roback R., Williams-Jones A.E. (2018). A spectroscopic study of uranyl speciation in chloride-bearing solutions at temperatures up to 250 °C. Geochim. Cosmochim. Acta.

[60] Szabó Z., Glaser J., Grenthe I. (1996). Kinetics of ligand exchange reactions for uranyl(2+) fluoride complexes in aqueous solution. Inorg. Chem..

[61] Jung W.-S., Harada M., Tomiyasu H., Fukutomi H. (1988). Oxygen-17 NMR study of the uranyl ion. V. Kinetics and mechanisms of formation and decomposition reactions of Di-. MU.-hydroxo-bis-[uranyl(VI)] ion in aqueous nitrate solutions. Bull. Chem. Soc. Jpn..

[62] Moll H., Reich T., Szabo Z. (2000). The hydrolysis of dioxouranium(VI) investigated using EXAFS and ^17^O-NMR. Radiochim. Acta.

[63] Banyai I., Glaser J., Micskei K., Toth I., Zekany L. (1995). Kinetic behavior of carbonate ligands with different coordination modes: Equilibrium dynamics for uranyl(2+) carbonato complexes in aqueous solution. A ^13^C and ^17^O NMR study. Inorg. Chem..

[64] Glasoe P.K., Long F. (1960). Use of glass electrodes to measure acidities in deuterium oxide^1,2^. J. Phys. Chem..

[65] Szabo Z., Grenthe I. (2007). Reactivity of the “yl”-Bond in Uranyl (VI) Complexes. 1. Rates and Mechanisms for the Exchange between the trans-dioxo Oxygen Atoms in (UO_2_)_2_(OH)_2_^2+^ and Mononuclear UO_2_(OH)_n_^2-n^ Complexes with Solvent Water. Inorg. Chem..

[66] Réal F., Vallet V., Wahlgren U., Grenthe I. (2008). Ab Initio Sstudy of the Mechanism for Photoinduced Yl-Oxygen Exchange in uranyl(VI) in Acidic Aqueous Solution. J. Am. Chem. Soc..

[67] Ikeda A., Hennig C., Rossberg A., Tsushima S., Scheinost A.C., Bernhard G. (2008). Structural determination of individual chemical species in a mixed system by iterative transformation factor analysis-based X-ray absorption spectroscopy combined with UV− Visible absorption and quantum chemical calculation. Anal. Chem..

[68] Hennig C., Servaes K., Nockemann P., Van Hecke K., Van Meervelt L., Wouters J., Fluyt L., Görller-Walrand C., Van Deun R. (2008). Species distribution and coordination of uranyl chloro complexes in acetonitrile. Inorg. Chem..

[69] Brinkmann H., Patzschke M., Kaden P., Raiwa M., Rossberg A., Kloditz R., Heim K., Moll H., Stumpf T. (2019). Complex formation between UO_2_^2+^ and α-isosaccharinic acid: Insights on a molecular level. Dalton Trans..

[70] Binstead R., Zuberbühler A., Jung B. (2005). SPECFIT Global Analysis System.

[71] Gans P., Sabatini A., Vacca A. (1996). Investigation of equilibria in solution. Determination of equilibrium constants with the HYPERQUAD suite of programs. Talanta.

[72] Guo Y., Liu H., Cao H., Dong X., Wang Z., Chen J., Xu C. (2023). Complexation of uranyl with benzoic acid in aqueous solution at variable temperatures: Potentiometry, spectrophotometry and DFT calculations. Dalton Trans..

[73] Gampp H., Maeder M., Meyer C.J., Zuberbühler A.D. (1985). Calculation of equilibrium constants from multiwavelength spectroscopic data—III: Model-free analysis of spectrophotometric and ESR titrations. Talanta.

[74] Gampp H., Maeder M., Meyer C.J., Zuberbühler A.D. (1986). Calculation of equilibrium constants from multiwavelength spectroscopic data—IV: Model-free least-squares refinement by use of evolving factor analysis. Talanta.

[75] Gampp H., Maeder M., Meyer C.J., Zuberbühler A.D. (1985). Calculation of equilibrium constants from multiwavelength spectroscopic data—I: Mathematical considerations. Talanta.

[76] Günther A., Geipel G., Bernhard G. (2007). Complex formation of uranium(VI) with the amino acids L-glycine and L-cysteine: A fluorescence emission and UV-Vis absorption study. Polyhedron.

[77] Günther A., Steudtner R., Schmeide K., Bernhard G. (2011). Luminescence properties of uranium(VI) citrate and uranium(VI) oxalate species and their application in the determination of complex formation constants. Radiochim. Acta.

[78] Lütke L., Moll H., Bernhard G. (2012). A new uranyl benzoate species characterized by different spectroscopic techniques. Radiochim. Acta.

[79] Rutledge D., Barros A. (2002). Durbin–Watson statistic as a morphological estimator of information content. Anal. Chim. Acta.

[80] Drobot B., Steudtner R., Raff J., Geipel G., Brendler V., Tsushima S. (2015). Combining luminescence spectroscopy, parallel factor analysis and quantum chemistry to reveal metal speciation–a case study of uranyl(VI) hydrolysis. Chem. Sci..

[81] Perdew J.P., Burke K., Ernzerhof M. (1996). Generalized gradient approximation made simple. Phys. Rev. Lett..

[82] Ahlrichs R., Bär M., Häser M., Horn H., Kölmel C. (1989). Electronic structure calculations on workstation computers: The program system turbomole. Chem. Phys. Lett..

[83] Cao X., Dolg M. (2004). Segmented contraction scheme for small-core actinide pseudopotential basis sets. J. Mol. Struct. THEOCHEM.

[84] Klamt A., Schüürmann G. (1993). COSMO: A new approach to dielectric screening in solvents with explicit expressions for the screening energy and its gradient. J. Chem. Soc. Perkin Trans. 2.

[85] Miertuš S., Scrocco E., Tomasi J. (1981). Electrostatic interaction of a solute with a continuum. A direct utilizaion of AB initio molecular potentials for the prevision of solvent effects. Chem. Phys..

