# Supplementary material for: Uranyl(VI) Interaction with 2-Phosphonobutane-1,2,4-Tricarboxylic Acid (PBTC): A Spectroscopic and Computational Study over a Wide pH Range
Source: Molecules. 2025 Oct 21;30(20):4144. doi: 10.3390/molecules30204144 (PMC12566417; doi:10.3390/molecules30204144)
Supplement: Supplementary file 1 [file molecules-30-04144-s001.zip › molecules-3878833-supplementary.pdf]

# Supporting Information

## Uranyl(VI) interaction with 2-phosphonobutane-1,2,4-tricarboxylic acid (PBTC): A spectroscopic and computational study over a wide pH range

**Jerome Kretzschmar [1,*], Anne Wollenberg [2,§], Ion Chiorescu [3], Sven Krüger [3], Ronja Kraft [4], Michael U. Kumke [4], Satoru Tsushima [1,5], Katja Schmeide [1] and Margret Acker [6,*]**

1 Institute of Resource Ecology, Helmholtz-Zentrum Dresden-Rossendorf, 01328 Dresden, Germany; s.tsushima@hzdr.de (S.T.); k.schmeide@hzdr.de (K.S.)
2 Radiochemistry and Radioecology, Technical University Dresden, 01062 Dresden, Germany; a.wollenberg@hzdr.de
3 Department of Chemistry, School of Natural Sciences, Technical University of Munich, 87547 Garching, Germany; chiorescu@mytum.de (I.C.); krueger@ch.tum.de (S.K.)
4 Institute of Chemistry, Faculty of Science, University Potsdam, 14476 Potsdam, Germany; kraft2@uni-potsdam.de (R.K.); kumke@uni-potsdam.de (M.U.K.)
5 Laboratory for Zero-Carbon Energy, Institute of Science Tokyo, Tokyo 152-8550, Japan
6 Radiation Protection and Central Radionuclide Laboratory, Technical University Dresden, 01062 Dresden, Germany
* Correspondence: j.kretzschmar@hzdr.de (J.K.); margret.acker@tu-dresden.de (M.A.)
§ Current address: Helmholtz-Institute Freiberg for Resource Technology, Helmholtz-Zentrum Dresden-Rossendorf, 09599 Freiberg, Germany.

## U(VI)-PBTC complex speciation

### Raman spectroscopy

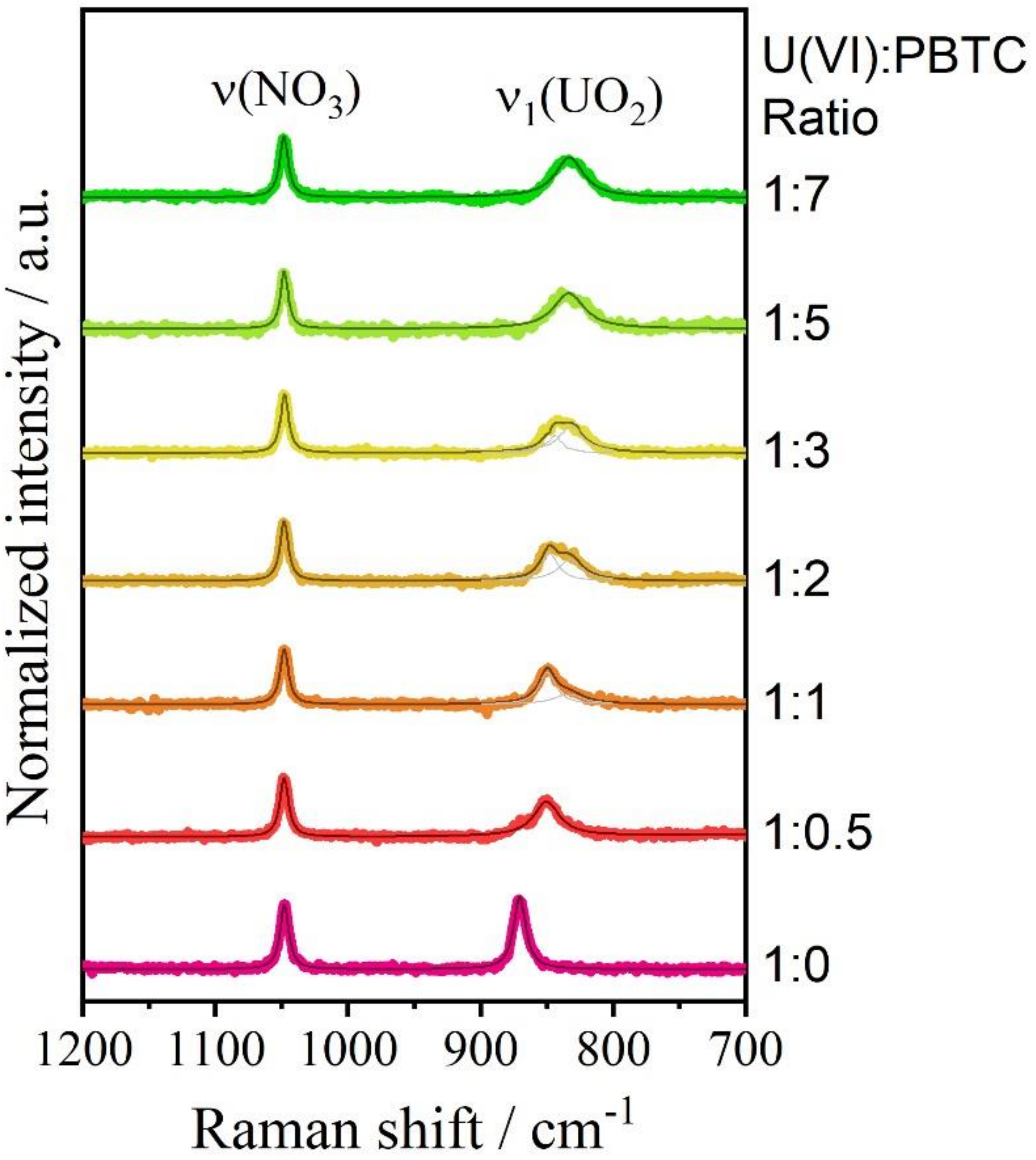


**Figure S1.** Raman spectra obtained at different U(VI):PBTC ratios (black lines correspond to Lorentzian line fitting, grey lines indicate individual components where applicable with sufficient accuracy); [U(VI)] = 10 mM, $I_m$ = 0.5 m NaCl, pH = 2.

**Table S1.** Results of Raman studies: (A) Peak position (± 1 $cm^{-1}$) of Raman-active symmetric stretching vibration mode $\nu_1(UO_2)$ of U(VI)-PBTC samples for the concentration series at pH 2. (B) Incremental Raman shift changes ($\Delta\nu_1$) were determined under the assumption of different numbers *n* of coordinated PBTC ligand using Equation 1 between the Raman vibration of free U(VI) and the highest Raman shift observed at a U(VI):PBTC ratio of 1:7. Based on these values, the Raman shift of $\nu_1(UO_2)$ of a potential 1:1 complex at pH 2 was calculated. (C) Raman mode maxima of U(VI)-PBTC samples for pH-series for U(VI):PBTC ratio of 1:7.

**(A)**

| U:PBTC ratio | Raman shift, $cm^{-1}$ | FWHM[a], $cm^{-1}$ |
|---|---|---|
| free $UO_2^{2+}$ | 871 | 11 |
| 1:0.5 | 851 | 27 |
| 1:1 | 850, (834)[b] | 15, (28) |
| 1:2 | 849, 833 | 15, 22 |
| 1:3 | 833, (849) | 25, (15) |
| 1:5 | 833 | 24 |
| 1:7 | 833 | 30 |

[a] Full width at half maximum. [b] Values in parentheses refer to bands of low intensity due to species present in small quantities (alongside a second, predominating species).

**(B)**

| assumed maximum number of coordinated ligands *n* | $\Delta\nu_1$, $cm^{-1}$ (slope) | calculated Raman shift $\nu_1$ for a 1:1 complex, $cm^{-1}$ |
|---|---|---|
| 1 | −38.0 | 833 |
| 2 | −19.0 | 852 |
| 3 | −12.7 | 858 |
| 4 | −9.5 | 861 |
| 5 | −7.6 | 863 |

**(C)**

| | Raman shift, $cm^{-1}$ | FWHM, $cm^{-1}$ | $\Delta\nu_1$, $cm^{-1}$ |
|---|---|---|---|
| free $UO_2^{2+}$ | 871 | 11 | 0 |
| +PBTC, pH 2 | 833 | 27 | 38 |
| +PBTC, pH 3 | 828 | 25 | 5 |
| +PBTC, pH 4 | 825 | 25 | 3 |
| +PBTC, pH 5 | 823 | 24 | 2 |
| +PBTC, pH 6 | 822 | 24 | 1 |
| +PBTC, pH 7 | 816 | 26 | 6 |
| +PBTC, pH 8 | 811 | 25 | 5 |
| +PBTC, pH 9 | 809 | 27 | 3 |
| +PBTC, pH 10 | 808 | 26 | 1 |
| +PBTC, pH 11 | 804 | 30 | 4 |

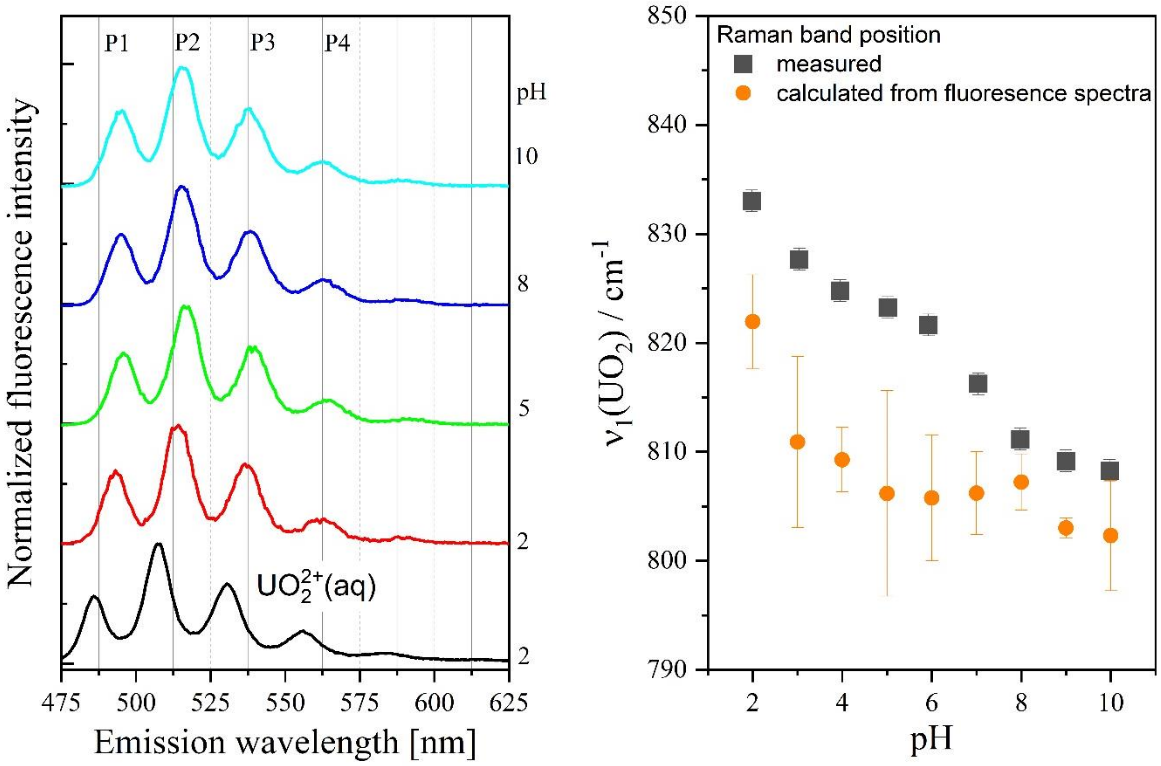


**Figure S2.** Example of TRLFS spectra at the initial delay time 0.5 µs obtained at different pH, [U(VI)] = 10 mM, [U(VI)]:[PBTC] = 1:5 (left), Correlation of the experimentally determined $\nu_1(UO_2)$ (values from Table S1C) with those calculated from the spacing of neighboring main peaks (vibrational fine structure) in the luminescence spectra of the U(VI)-PBTC samples according to Drobot et al. [1] (right).

**Table S2.** Characteristics of the pH-dependent luminescence spectra obtained at $T$ = 4 K, [U(VI)] = 10 mM, [U(VI)]:[PBTC] = 1:5, $I_m$ = 0.5 m NaCl.

| | **peak position / nm** | | | | **converted into wavenumbers / cm$^{-1}$** | | | |
|---|---|---|---|---|---|---|---|---|
| **pH** | **P1** | **P2** | **P3** | **P4** | **P1** | **P2** | **P3** | **P4** |
| 2 | 493.2 | 514.2 | 536.7 | 561.5 | 20273.7 | 19448.7 | 18631.6 | 17807.9 |
| 3 | 497.3 | 518.4 | 541.2 | 565.8 | 20107.2 | 19290.2 | 18476.5 | 17674.5 |
| 4 | 497.0 | 517.9 | 540.5 | 565.2 | 20120.9 | 19308.3 | 18501.5 | 17693.1 |
| 5 | 495.8 | 516.7 | 539.2 | 563.4 | 20167.7 | 19352.2 | 18545.8 | 17749.2 |
| 6 | 495.5 | 516.2 | 538.7 | 562.9 | 20182.5 | 19371.5 | 18564.8 | 17765.2 |
| 7 | 495.1 | 515.8 | 538.2 | 562.5 | 20197.2 | 19386.9 | 18581.4 | 17778.6 |
| 8 | 495.0 | 515.7 | 538.1 | 562.5 | 20200.4 | 19390.7 | 18583.2 | 17778.7 |
| 9 | 495.9 | 516.5 | 538.8 | 563.2 | 20166.1 | 19362.2 | 18560.1 | 17757.0 |
| 10 | 495.0 | 515.5 | 537.8 | 561.9 | 20203.3 | 19398.0 | 18592.9 | 17796.4 |
| $UO_2^{2+}$ (1) | 486.4 | 507.4 | 530.1 | 555.5 | 20558.9 | 19709.6 | 18862.9 | 18002.8 |

Table S2 *continued*

| pH | Δ(P1-P2) / $cm^{-1}$ | Δ(P2-P3) / $cm^{-1}$ | Δ(P3-P4) / $cm^{-1}$ | average Δ / $cm^{-1}$ |
|---|---|---|---|---|
| 2 | 825.1 | 817.1 | 823.7 | 822.0 ± 4.3 |
| 3 | 817.0 | 813.6 | 802.0 | 810.9 ± 7.9 |
| 4 | 812.6 | 806.8 | 808.4 | 809.3 ± 3.0 |
| 5 | 815.5 | 806.3 | 796.7 | 806.2 ± 9.4 |
| 6 | 811.1 | 806.7 | 799.6 | 805.8 ± 5.8 |
| 7 | 810.3 | 805.4 | 802.8 | 806.2 ± 3.8 |
| 8 | 809.6 | 807.5 | 804.5 | 807.2 ± 2.6 |
| 9 | 803.9 | 802.1 | 803.1 | 803.0 ± 0.9 |
| 10 | 805.3 | 805.1 | 796.5 | 802.3 ± 5.1 |
| $UO_2^{2+}$ [a] | 849.4 | 846.6 | 860.1 | 852.0 ± 7.1 |

[a] U(VI) in 0.5 M NaCl, pH = 2, without PBTC.

Reference data from Demnitz et al. [2]

| species | mean peak position / nm | | | converted into wavenumber / $cm^{-1}$ | | |
|---|---|---|---|---|---|---|
| | P1 | P2 | P3 | P1 | P2 | P3 |
| $UO_2^{2+}$ (273 K) | 488.1 | 509.9 | 533.7 | 20487.6 | 19611.7 | 18737.1 |
| $UO_2^{2+}$ (153 K) | 484.0 | 505.6 | 529.0 | 20661.2 | 19778.5 | 18903.6 |
| $UO_2Cl^+$ (153 K) | 488.6 | 510.6 | 534.5 | 20466.6 | 19584.8 | 18709.1 |

| species | Δ(P1-P2) / $cm^{-1}$ | Δ(P2-P3) / $cm^{-1}$ | average Δ / $cm^{-1}$ |
|---|---|---|---|
| $UO_2^{2+}$ (273 K) | 875.9 | 874.6 | 875.2 ± 1.0 |
| $UO_2^{2+}$ (153 K) | 882.7 | 874.9 | 878.8 ± 5.5 |
| $UO_2Cl^+$ (153 K) | 881.8 | 875.7 | 878.8 ± 4.3 |

## UV-Vis spectroscopy

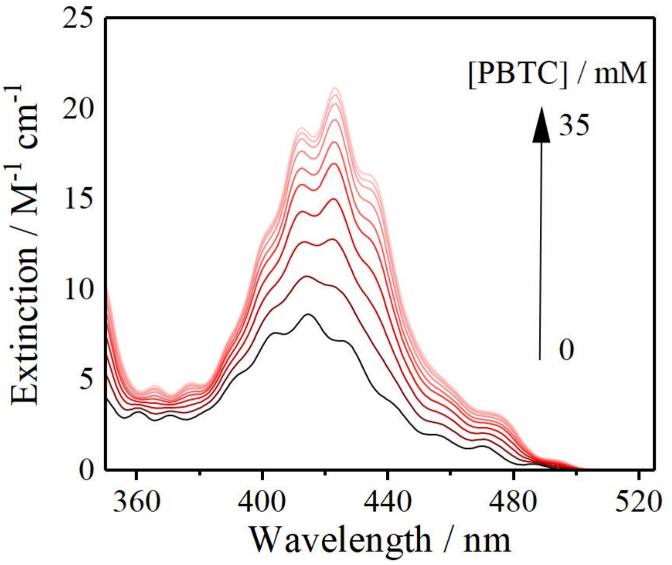


**Figure S3.** Exemplary UV-Vis spectra obtained from solutions of the U(VI)-PBTC system with varying PBTC concentration up to 35 mM at given [U(VI)] = 10 mM, pH = 2, $I_m$ = 0.5 m NaCl. The spectrum given in black corresponds to free $UO_2^{2+}$ at pH 2.

### Speciation analysis of UV-Vis data with Iterative Transformation Factor Analysis (ITFA)

The measured absorption spectra are a linear combination of the individual spectra of each spectroscopically active species in the solution, weighted according to their proportions based on the Beer-Lambert law (Eq. S1).

$$A_{ik}(\lambda_i) = d \cdot \sum_{j=1}^{n} e_{ij} \cdot c_{jk} \qquad \text{(Eq. S1)}$$

where $A_{ik}(\lambda_i)$ is the total absorption at wavelength $\lambda_i$ of the spectrum $k$, $d$ is the optical path length, $e_{ij}$ is the unknown extinction of the component $j$, and $c_{jk}$ is the concentration of the component $j$.

ITFA is a proven tool for decomposing such spectral mixtures into their spectral components [3-5]. At least 15 spectra of the concentration series and up to 70 spectra of the pH series were used to analyze the data. First, a Principal Component Analysis (PCA) was performed to determine the number of UV-Vis active uranyl components and their eigenvectors, followed by the model-independent VARIMAX rotation [6]. In that way, the eigenvectors can be transformed into factor loadings, which should be unimodal as a function of pH or PBTC concentration and correlate with a qualitative concentration profile of the corresponding uranyl species. The correct number of components, i.e. U(VI) species, was determined by considering the results of the indicator function IND (the minimum of IND gives the number of components), the abstract spectra of the eigenvectors (only those factors whose abstract plots are visibly different from noise should be included), as well as the abstract concentration distribution and the results of the VARIMAX rotation (should have a smooth shape or unimodal distribution) [4,7].

Results of a representative example for the use of ITFA in this study

The statistical results of the ITFA analysis for the first 12 factors[1] are shown in Table S3. The analysis of the eigenvalues, which represent a value for the weight of a factor, shows a strong decrease in the values from the first to the second factor and a further significant decrease up to about factor 7. Only small changes are observed for the following factors. Therefore, these factors do not play a role. The indicator function (IND) and the imbedded error (IE) result in 11 active components. However, it should be noted that this method can fail, especially in the case of UV-Vis spectra. The main reason for this is that the systematic error in the data is greater than its stochastic error, so too many components appear to be predicted here. From factor 8 onwards, there are only minor changes in the values of IND and IE, so that the presence of 8 or 9 components is more likely to be inferred. The percentage residual variance (PRV), which is often used as a criterion for determining the number of factors [8] and should be close to zero for the respective factor, indicates also that 8 or 9 components are more likely to be active.

Inspection of the abstract spectra (Figure S4) shows that component 8 still has a 'spectrum' and the spectra of the following components only represent the error in the data and can therefore be neglected. The same picture is given when analyzing the abstract concentration distribution. Up to factor 8 the abstract concentrations show a smooth shape, from factor 9 on the profile becomes noisier. To summarize, it can be concluded that 8 U(VI) species (= components) are present in both the ligand concentration and the pH series of the UV-Vis studies. The factor loadings obtained from the VARIMAX rotation, whose maxima represent the highest proportion of the corresponding component, are shown in Figure S5.

**Table S3.** Eigenvalues $\lambda_i$, real error (RE), imbedded error (IE), extracted error (XE), indicator function (IND), and percentage residual variance (PRV) as a function of the number of factors ($n$) used for data reproduction.

| number of factors | Eigenvalue $\lambda_i$ | RE($n$)·10$^4$ | IE($n$)·10$^4$ | XE($n$)·10$^4$ | IND($n$)·10$^4$ | PRV($n$) |
|---|---|---|---|---|---|---|
| | | | | | | |
| 1 | 3.350E+3 | 444.6962 | 52.7757 | 441.5535 | 90.7543 | 0.93336 |
| 2 | 2.850E+1 | 139.5671 | 23.4244 | 137.5873 | 29.3147 | 0.09062 |
| 3 | 2.719E+0 | 47.2100 | 9.7043 | 46.2018 | 10.2098 | 0.01022 |
| 4 | 2.029E−1 | 30.5627 | 7.2542 | 29.6893 | 6.8084 | 0.00422 |
| 5 | 1.054E−1 | 15.7464 | 4.1787 | 15.1818 | 3.6149 | 0.00110 |
| 6 | 2.883E−2 | 7.5660 | 2.1994 | 7.2392 | 1.7908 | 0.00025 |
| 7 | 3.731E−3 | 5.7072 | 1.7920 | 5.4185 | 1.3934 | 0.00014 |
| 8 | 2.197E−3 | 4.2187 | 1.4161 | 3.9739 | 1.0629 | 0.00008 |
| 9 | 8.082E−4 | 3.5167 | 1.2521 | 3.2862 | 0.9148 | 0.00005 |
| 10 | 3.337E−4 | 3.1891 | 1.1969 | 2.9560 | 0.8571 | 0.00004 |
| 11 | 1.048E−4 | 3.0942 | 1.2179 | 2.8444 | 0.8595 | 0.00004 |
| 12 | 7.150E−5 | 3.0339 | 1.2473 | 2.7657 | 0.8716 | 0.00004 |

where $RE(n) = \left(\frac{\sum_{j=n+1}^{c} \lambda_j^0}{r(c-n)}\right)^{\frac{1}{2}}$, $IND(n) = \frac{RE_n}{(c-n)^2}$, $IE(n) = RE(n)\left(\frac{n}{c}\right)^{\frac{1}{2}}$,

$XE(n) = RE(n)\left(\frac{c-n}{c}\right)^{\frac{1}{2}}$, $PRV(n) = 100\left(\frac{\sum_{j=n+1}^{c} \lambda_j^0}{\sum_{j=1}^{c} \lambda_j\ )}\right)$

[1] In the context of ITFA, “factors” refer to the mathematical–statistical dimensions obtained from the factor analysis (e.g., eigenvalue analysis, factor loadings), whereas “components” denote the corresponding physical or chemical entities (e.g., spectral components or chemical species).

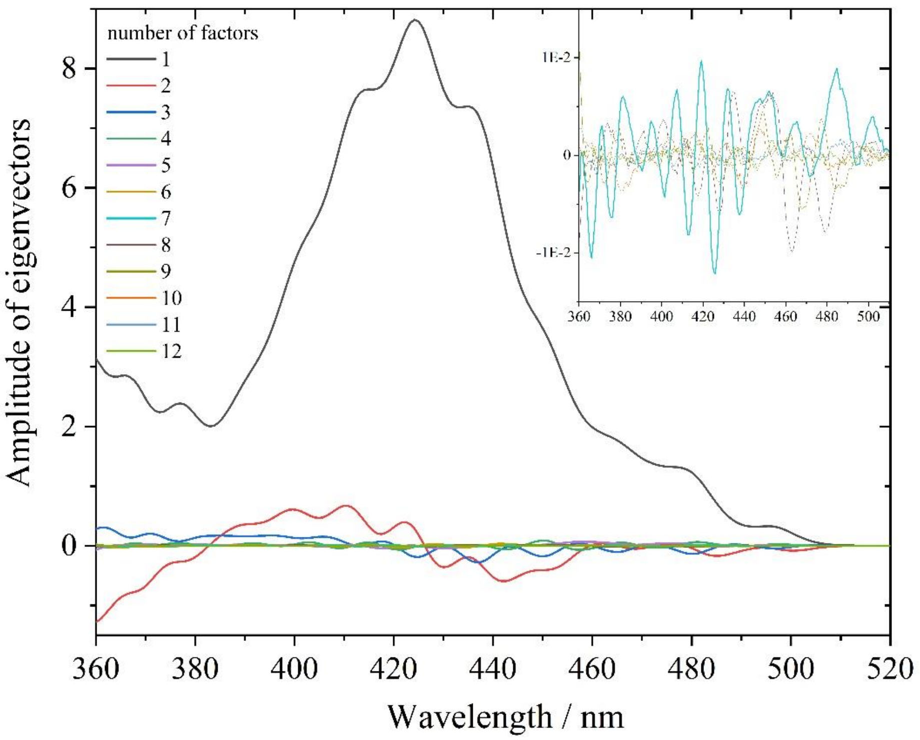


**Figure S4.** Abstract spectra of the first 12 factors. The insert shows a magnification for the cases of 7 through 12 factors.

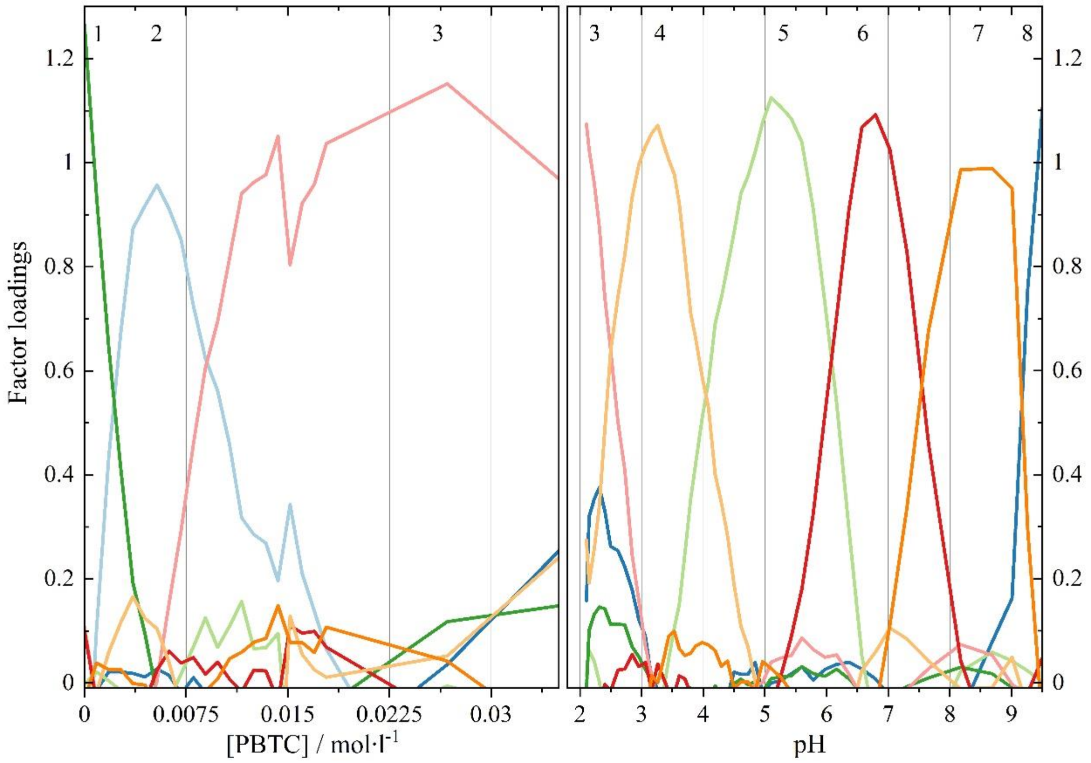


**Figure S5.** Factor loading from VARIMAX rotation calculated including 8 components for concentration series (left) and pH series (right), (numbers in the diagram = number of components) [U(VI)] = 10 mM, [PBTC] = 35 mM.

## TRLFS measurements

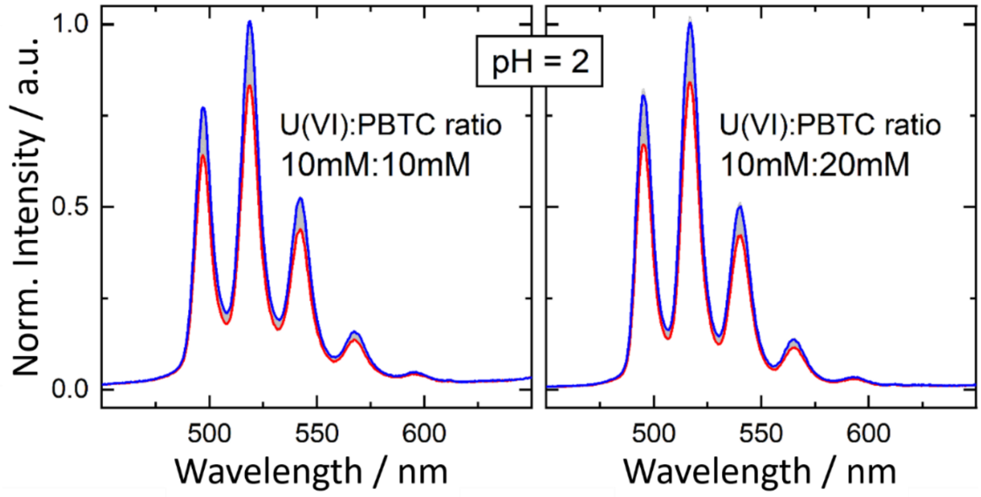


**Figure S6.** Luminescence spectra of U(VI)–PBTC complexes at pH = 2 for two different U(VI):PBTC ratios recorded every 10 s for 20 minutes (equal to approx. 12000 laser pulses) in total. Spectra ranging from blue to red correspond to exposure times of between 10 s and 20 minutes

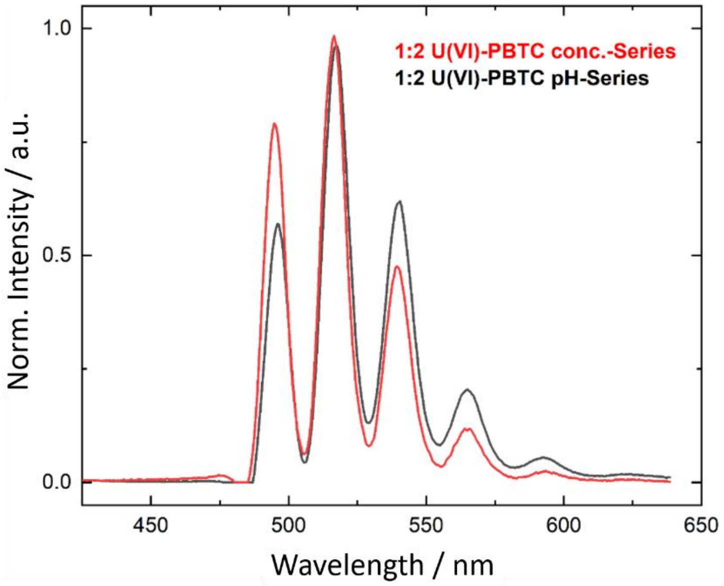


**Figure S7.** Species-associated TRLFS spectra of the presumed U(VI)-PBTC 1:2 complex obtained from PARAFAC analyses extracted from concentration series and pH series, respectively.

# Characterization of the main U(VI)-PBTC complex structure

## ATR FT-IR data to determine the binding motif

**Table S4.** Infrared (IR) absorption bands of PBTC and of some selected compounds for comparison.

| Vibrational mode | PBTC | | | Citric acid [9] | | HEDP[a] [10,11] | |
|---|---|---|---|---|---|---|---|
| | pH 2 | pH 5 | pH 8 | pH 1–2 | pH 8–12 | pH 2 | pH 6–9 |
| $\nu_s$(HOC=O) | 1715 | | | 1722, 1724 | | | |
| $\nu_{as}$($COO^-$) | | 1556 | 1551 | | 1568, 1565 | | |
| ν(C–H) | 1410 | | | | | | |
| $\nu_s$($COO^-$) | | 1399 | | 1399 | 1390, 1389 | | |
| ν(C–OH) | 1270 | | | | 1095, 1100 | | |
| ν(-P=O) | 1206 | 1164 | 1149 | | | 1179 | 1151, 1174–1177 |
| $\nu_s$(P-OH) | 903 | 898 | 872 | | | 908 | 968 |
| $\nu_{as}$(P-OH) | 1073 | 1053 | 1047 | | | 1063 | 1072, 1044–1057 |

$\nu_s$ – symmetric stretching vibration, $\nu_{as}$ – antisymmetric stretching vibration,
[a] HEDP, 1-Hydroxyethylidene-1,1-diphosphonic acid

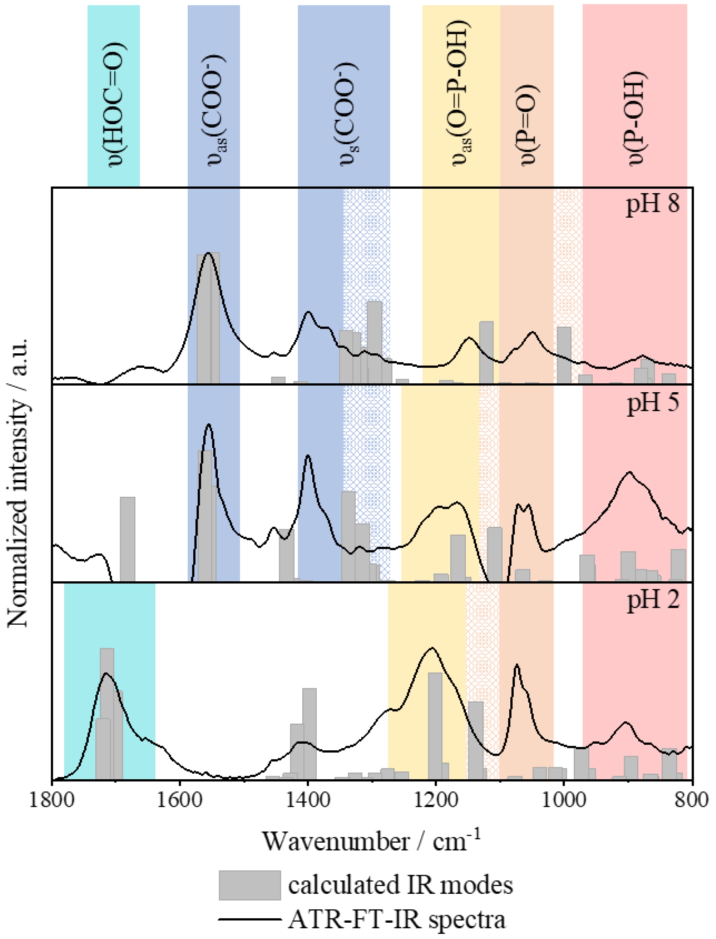


**Figure S8.** Assignment of important ATR FT-IR signals of PBTC at pH 2, 5, and 8, as indicated in Table S4.

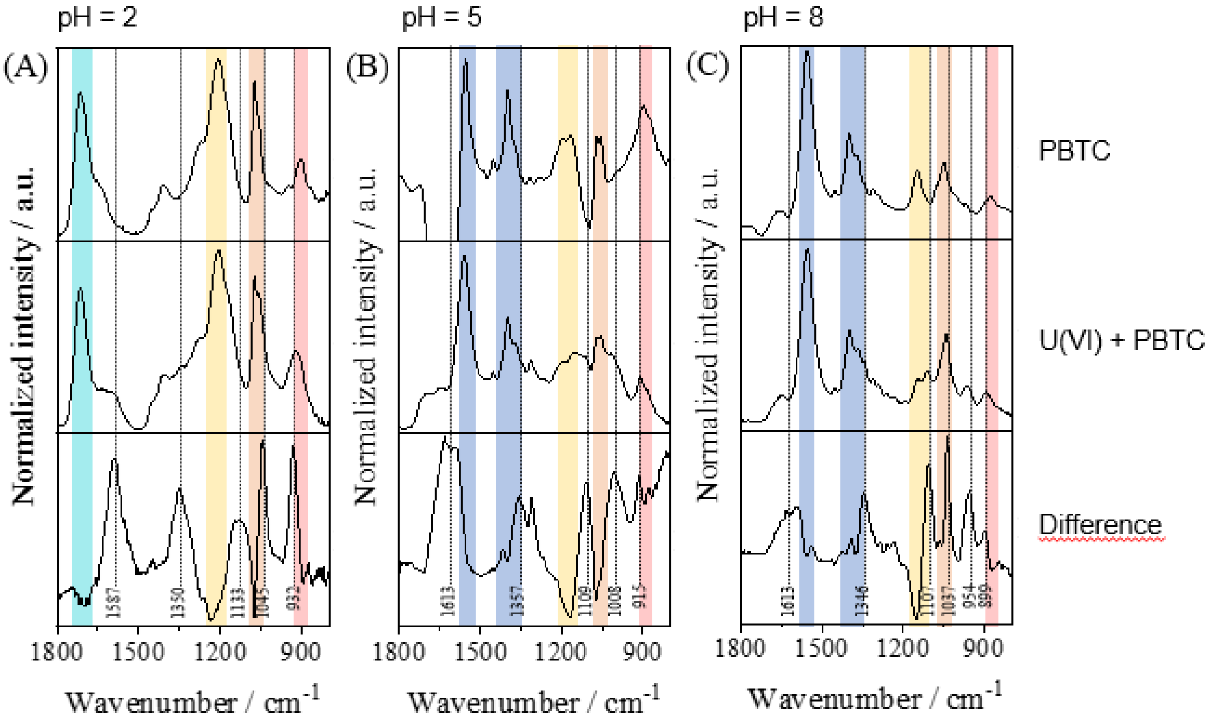


**Figure S9.** ATR FT-IR spectra of 25 mM PBTC only (top), U(VI)+PBTC solution with a ratio of 1:5 (middle), and the corresponding difference spectra of both (bottom) at pH 2 (A), pH 5 (B), and pH 8 (C) measured at room temperature. Difference spectra were calculated from pure PBTC spectra and U(VI) + PBTC spectra showing only the spectral differences occurring upon complexation. Vibrational modes of the pure ligand are marked with colors. Vibrational modes of the complex are marked with the identified wavenumber of the signal.

## Analysis of NMR data to identify structure features

*In general, the following NMR spectroscopic data are compiled in order of increasing pH.*

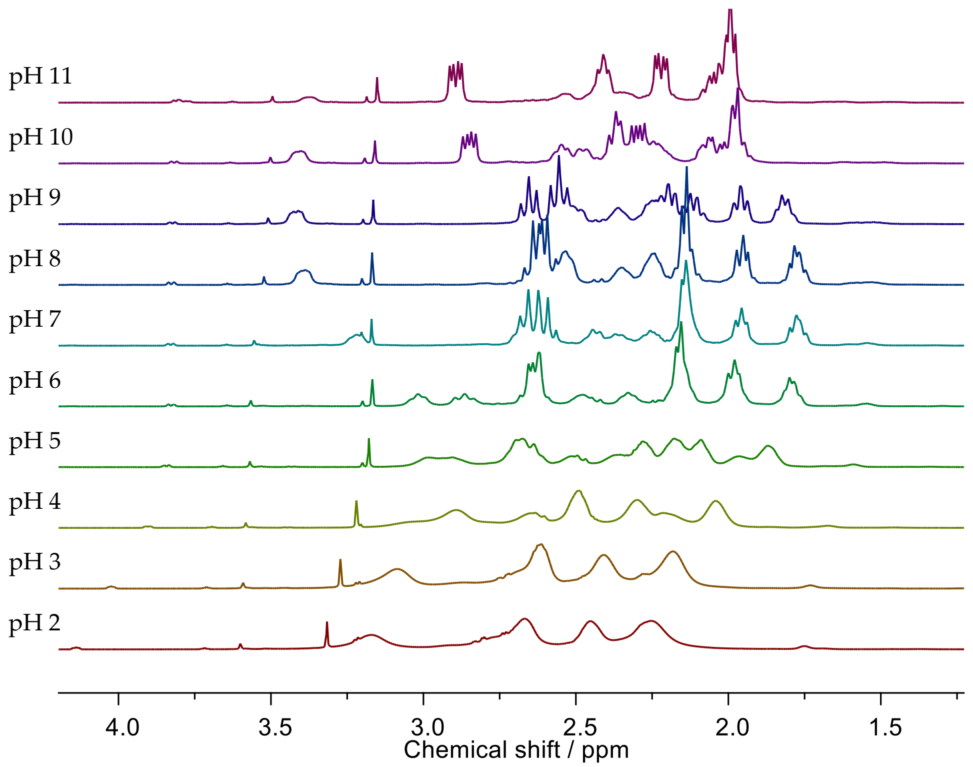


**Figure S10.** $^{1}H$ NMR spectra corresponding to the $^{31}P\{^{1}H\}$ NMR spectra shown in Figure 1C. Spectra were obtained from solutions 40 mM in U(VI) and 290 mM in PBTC, at pH values stated with the spectra.

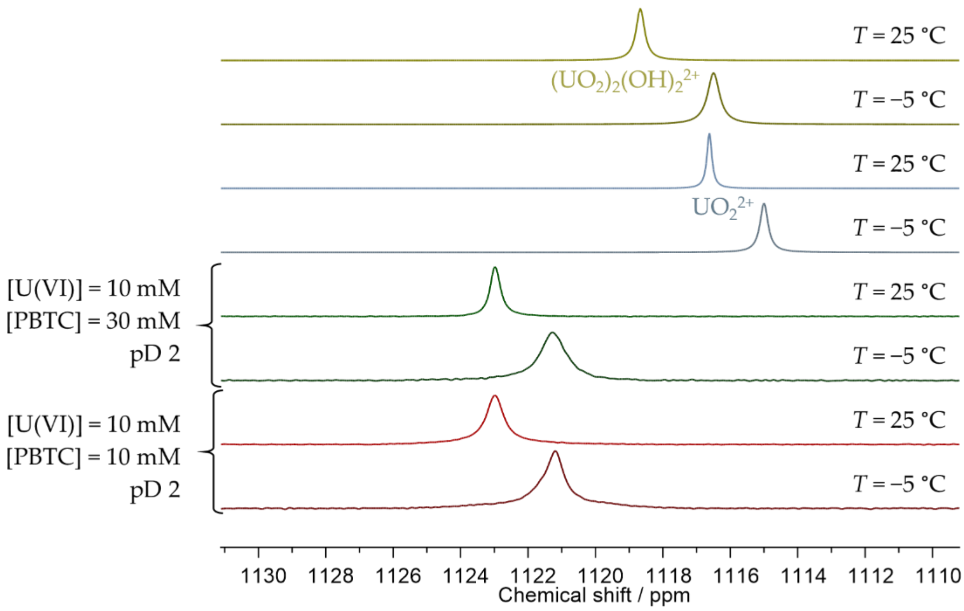


**Figure S11.** $^{17}O$ NMR spectra showing signals associated with uranyl oxygens, each recorded at two temperatures. Compared with spectra obtained for aquatic uranyl (hydrolysis) species [12], the downfield-shifted signals clearly indicate the interaction of U(VI) and PBTC at pD 2.

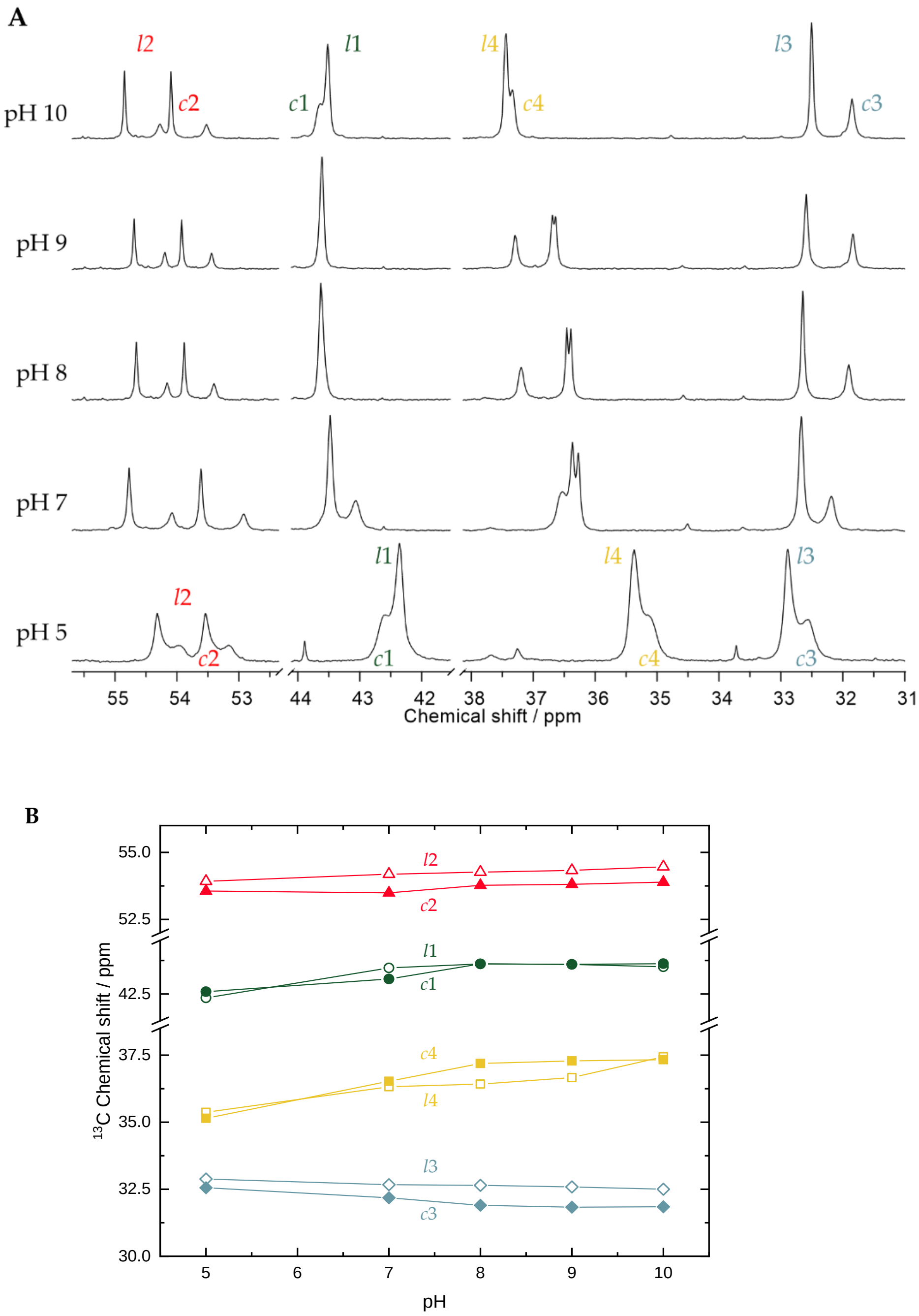


**Figure S12.** (A) Aliphatic region of the $^{13}C\{^{1}H\}$ NMR spectra shown in Figure 3. Spectra were obtained from solutions 40 mM in U(VI) and 290 mM in PBTC, at pH values as stated with the spectra. Signal assignment is according to the labeling in Figure 1A, indicating signals of the free ligand and the U(VI) complex by *l* and *c*, respectively. Note that the pH 7 spectrum was recorded at 100 MHz (9.4 T) while the other spectra were recorded at 150 MHz (14.1 T). (B) Graphic depicting pH-dependent chemical shifts corresponding to the spectra in (A).

Overall, for corresponding pH, differences in the $^{13}C$ NMR chemical shifts of free and U(VI)-bound PBTC's backbone carbons are rather small. As can be seen for the pH 5 spectrum, at low pH values signals broaden significantly and tend to merge (average) owing to notably faster ligand exchange reaction rates (see also Figures 1C and S10).

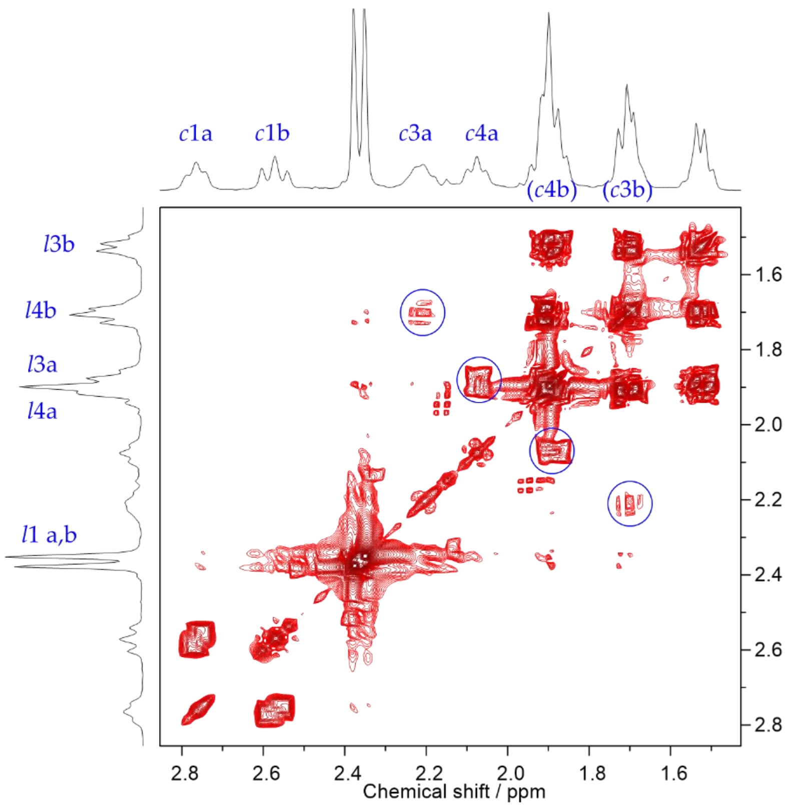


**Figure S13.** $^1H,^1H$-COSY NMR spectrum obtained at $T = 0$ °C from a pH 6 solution 10 mM in U(VI) and 50 mM in PBTC. Signal assignment is according to the labeling in Figure 1A, indicating diastereotopic hydrogens by a and b, and signals of the free ligand and the U(VI) complex by *l* and *c*, respectively. In the one-dimensional $^1H$ spectrum, the signals due to complex's H-3b and H-4b are obscured by the signals of the excess free ligand but can be assigned from the 2D correlation signals (circles).

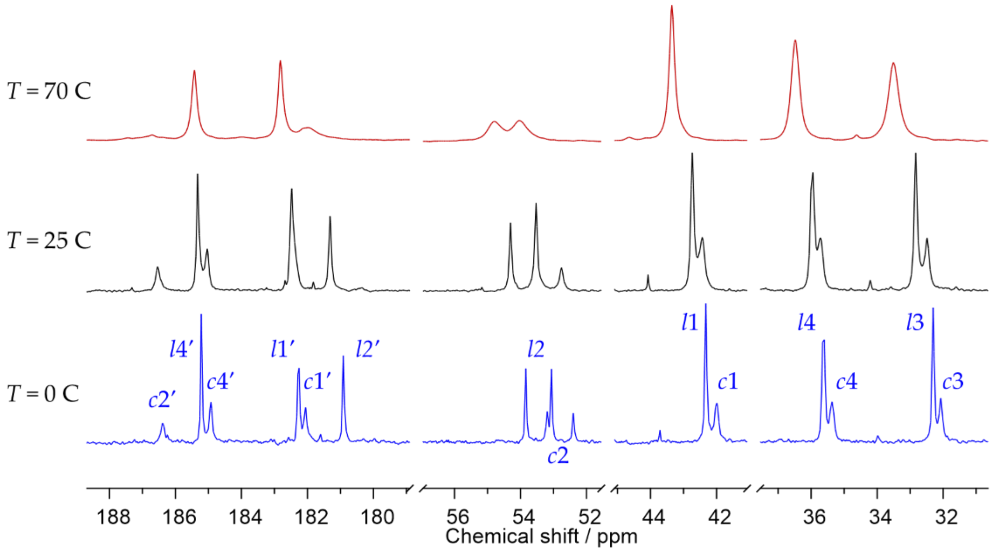


**Figure S14.** $^{13}C\{^1H\}$ VT NMR spectra obtained from a solution 40 mM in U(VI) and 290 mM in PBTC at pH 6. Signal assignment is according to the labeling in manuscript Figure 1A, indicating signals of the free ligand and the U(VI) complex by *l* and *c*, respectively. Owing to the exchange dynamics (see also Figure S21) between free and bound ligand, being very slow at 0 °C and fast at 70 °C, the signals' line width and resolution is very well at low temperature while upon heating averaging causes broad signals. All signals exhibit positive temperature coefficients.

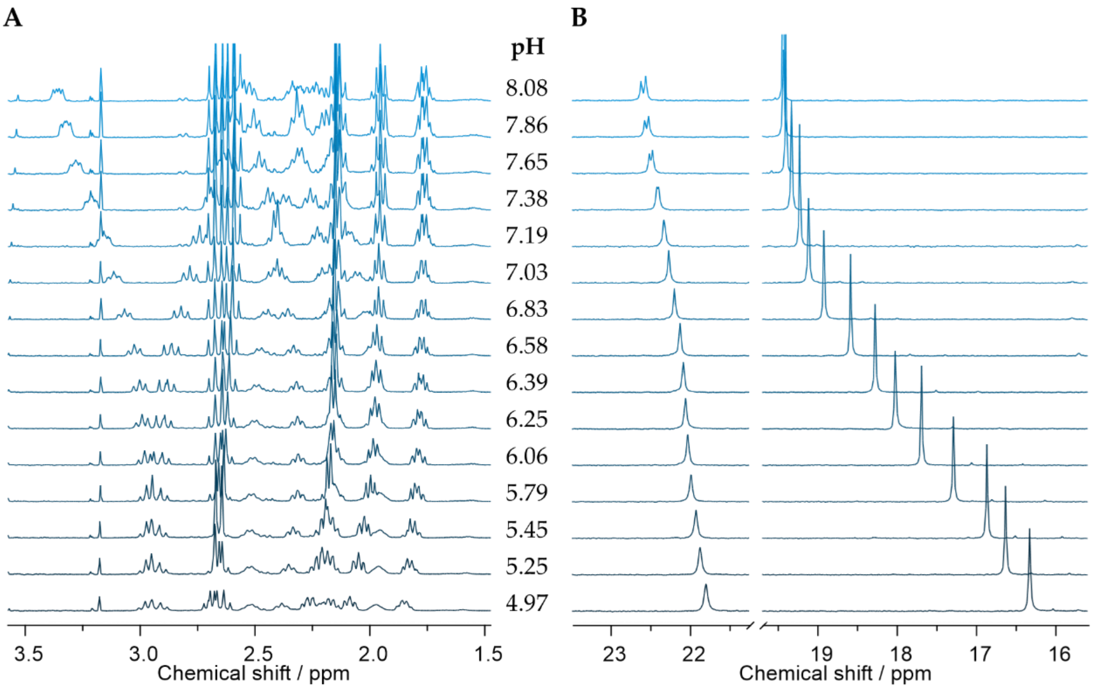


**Figure S15.** $^{1}H$ (A) and $^{31}P\{^{1}H\}$ NMR spectra (B) of solutions 10 mM in U(VI) and 50 mM in PBTC at pH values stated with the spectra.

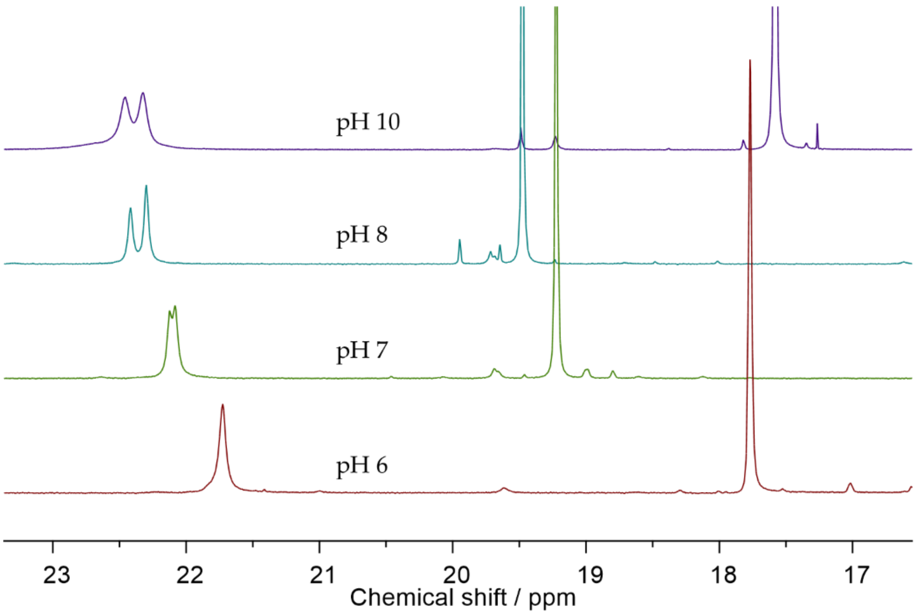


**Figure S16.** $^{31}P\{^{1}H\}$ NMR spectra measured at $T = 0$ °C corresponding to the spectra depicted in Figure 1C. Solutions were 40 mM in U(VI) and 290 mM in PBTC at pH values stated with the spectra. Acquisition at low temperature improves resolution upon slowing down ligand exchange dynamics (cf. Figure S14), thereby revealing the signal of U(VI)-bound PBTC to feature in fact two resonances – as discussed with Figure S19. Note the much larger pH-dependent $^{31}P$ signal displacements of the unbound ligand compared to those of the U(VI)-bound ligand.

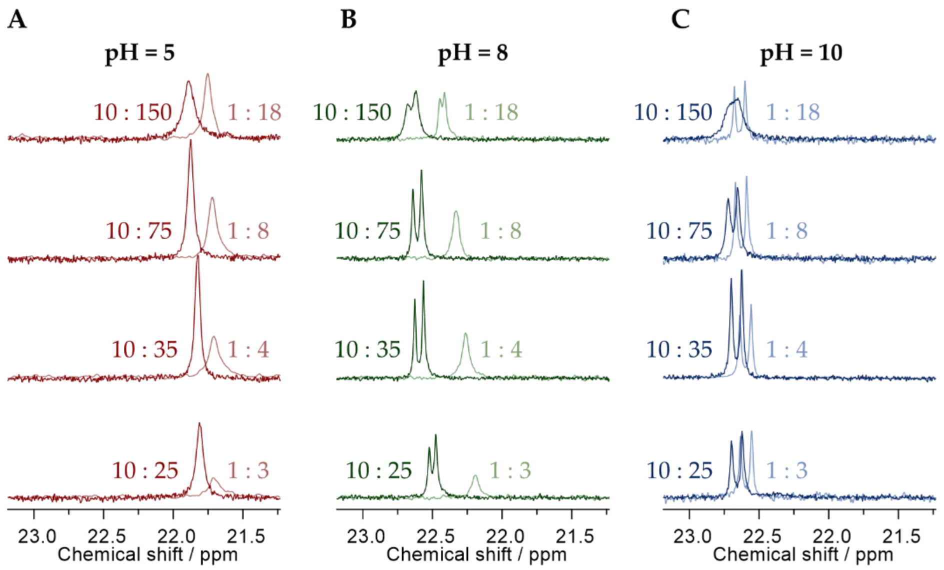


**Figure S17.** $^{31}P\{^{1}H\}$ NMR spectra of solutions obtained at pH 5 (A), pH 8 (B), and pH 10 (C), containing either 1 mM (pale colored spectra) or 10 mM U(VI) (dark colored spectra) at varying U(VI):PBTC ratios (numerals denote concentrations in mM).

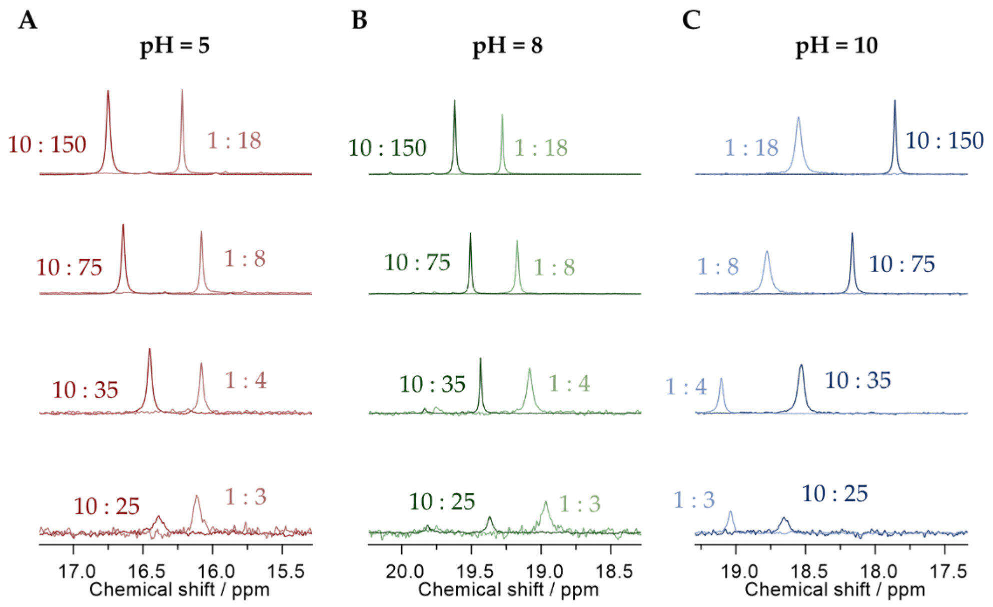


**Figure S18.** $^{31}P\{^{1}H\}$ NMR spectra of solutions obtained at pH 5 (A), pH 8 (B), and pH 10 (C) containing either 1 mM (pale colored spectra) or 10 mM U(VI) (dark colored spectra) at varying U(VI):PBTC ratios (numerals denote concentrations in mM) and at three different pH values as stated with the spectra. The spectra complement Figure S17, but here displaying the spectral region corresponding to signals of *unbound* PBTC. For better visualization, chemical shift regions differ (owing to pH) but using the same horizontal scaling; the vertical scaling is arbitrary.

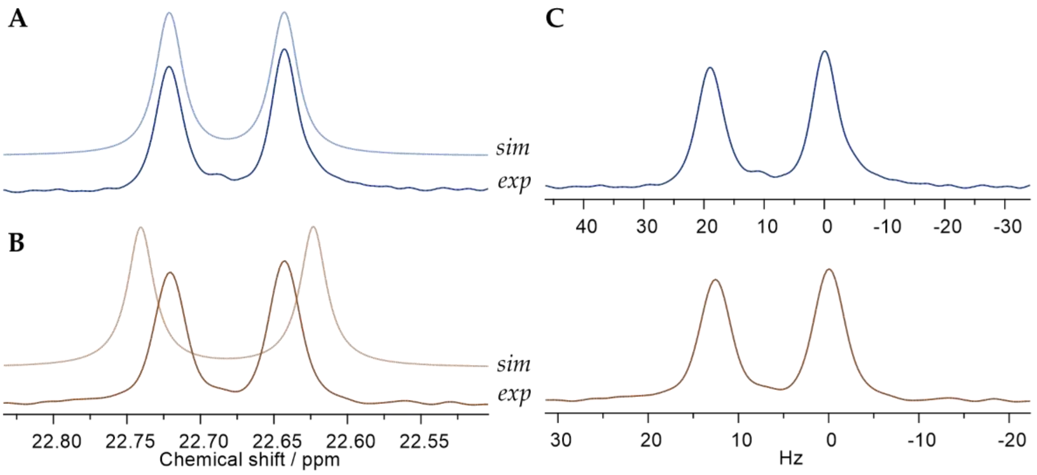


**Figure S19.** $^{31}P\{^1H\}$ NMR spectra corresponding to Figure S23D, acquired (*exp*) at $^{31}P$ resonance frequencies / field strengths of 242.884 MHz at 14.1 T (A) and 161.923 MHz at 9.4 T (B), expressed as chemical shift in ppm relative to $H_3PO_4$ and expressed in frequency units (C). Of course, regardless of the given field strength, chemical shifts are 22.721 and 22.643 ppm. At 14.1 T, the two lines are separated by 19.0 Hz, while at 9.4 T they are by 12.7 Hz. A line separation of 19.0 Hz at the respective field strength is shown by the simulated spectra (*sim*). Apparently, the two lines are no signal splitting (doublet) as it may be caused by any spin coupling, but in fact are two distinct resonance lines due to presence of diastereomers.

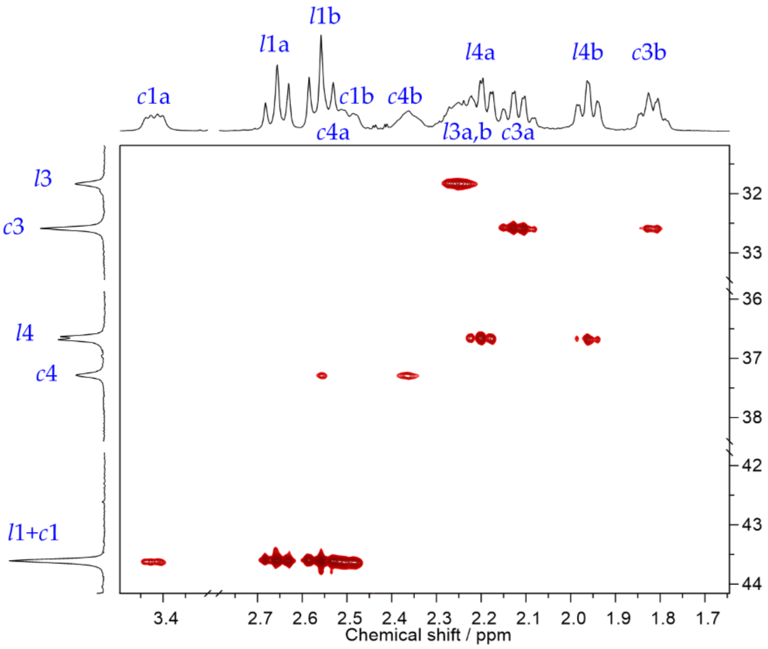


**Figure S20.** $^1H,^{13}C$-HSQC spectrum obtained from a pH 9 solution 40 mM in U(VI) and 290 mM in PBTC. Signal assignment is according to the labeling in Figure 1A, indicating diastereotopic hydrogens by a and b, and signals of the free ligand and the U(VI) complex by *l* and *c*, respectively.

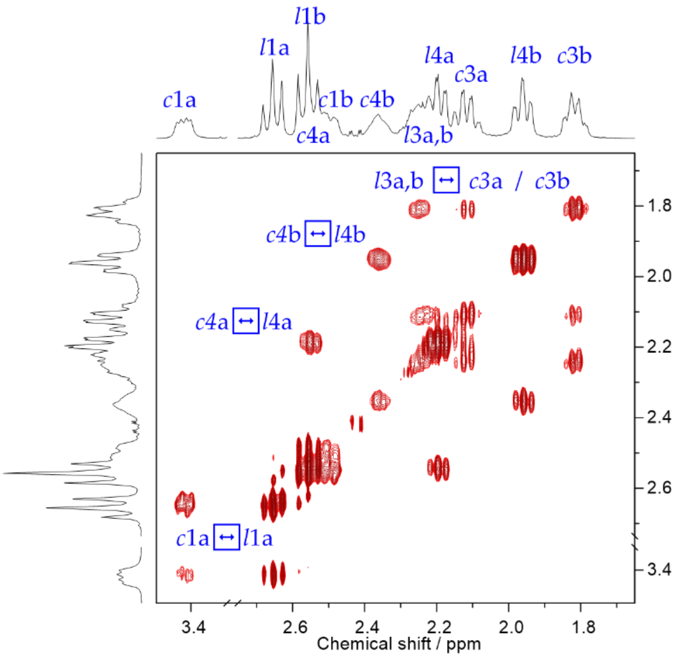


**Figure S21.** Phase-sensitive $^{1}$H,$^{1}$H-NOESY spectrum ($t_{mix}$ = 500 ms) obtained from a pH 9 solution 40 mM in U(VI) and 290 mM in PBTC. Off-diagonal signals being in phase with the diagonal indicates correlation signals due to chemical exchange (EXSY). This comprises both intramolecular and intermolecular processes such as exchanging sites unimolecularly within the same molecule (e.g., *c*3a vs. *c*3b, corresponding to conformational change) or bimolecularly between molecules upon displacing a bound ligand by a free ligand (e.g., *c*1a vs. *l*1a), respectively. Taking into account the broadening of some lines as well as the observation of distinct sets of signals (instead of signal averages) — for both free and bound ligand as well as for each individual diastereotopic hydrogen within a methylene group — the exchange processes are rather slow on the NMR time scale ($^{1}$H, 600 MHz, 25 °C).

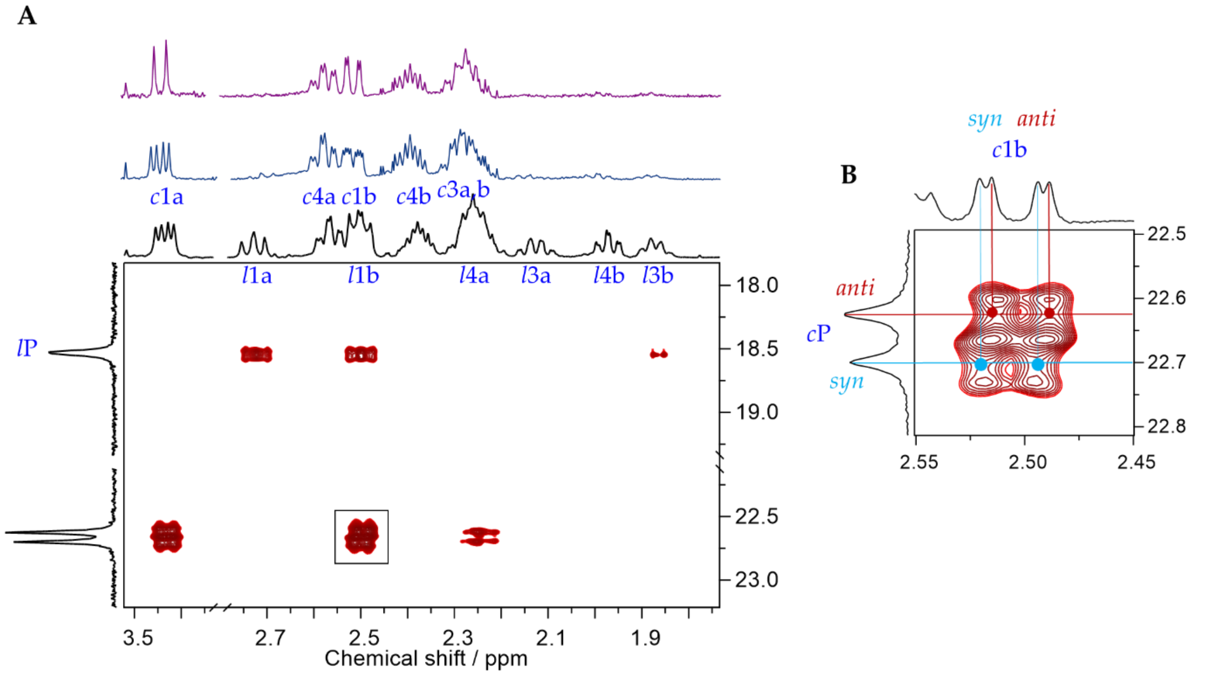


**Figure S22.** $^{1}$H,$^{31}$P-HMBC spectrum obtained from a pH 9 solution 10 mM in U(VI) and 30 mM in PBTC depicting $^{3}J_{P,H}$ between PBTC's phosphorus and H-1a, H-1b, and H-3b in both the free (excess) ligand (*l*) and the (1:2) complex (*c*), along with signal assignment corresponding to the labeling in Figure 1A. The above-aligned one-dimensional $^{1}$H NMR spectra correspond to a pH 10 sample initially 18 mM in U(VI) and 27 mM (cf. Figures S23, S24, and S25), demonstrating the invariant chemical shifts associated with the exclusive 1:2 complex. The indicated region (black rectangle) is magnified in (B), with the correlation signals indicating two just distinguishable *c*1b $^{1}$H resonances, correlating to the two distinct $^{31}$P resonances that hint to diastereomers (i.e., *syn* and *anti*) occurring for the 1:2 complex.

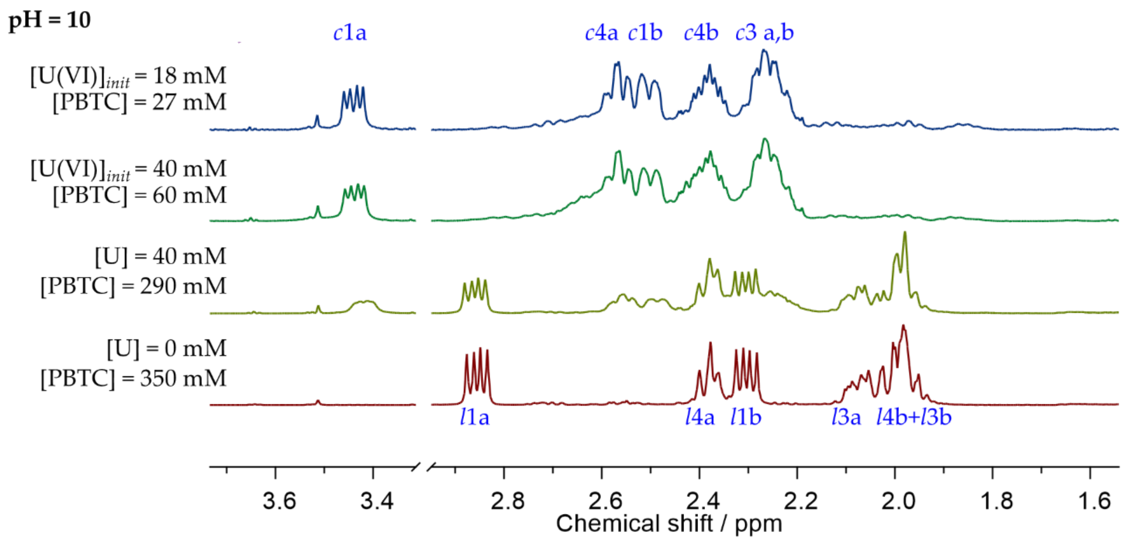


**Figure S23.** $^{1}H$ NMR spectra obtained from pH 10 solutions. The bottom spectrum refers to a blank solution, while the other spectra correspond to U(VI)-containing samples. Samples with U(VI):PBTC ratio initially (*init*) given as 1.5 showed uranyl hydroxide precipitation increasing the ligand excess resulting in formation of (only) the 1:2 complex (cf. Figure S24). Signal assignment is according to the labeling in Figure 1A, indicating diastereotopic hydrogens by a and b, and signals of the free ligand and the U(VI) complex by *l* and *c*, respectively.

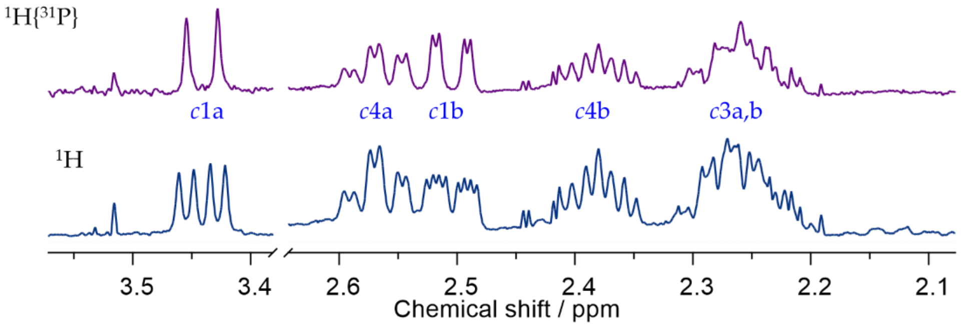


**Figure S24.** $^{31}P$-coupled (bottom) and $^{31}P$-decoupled (top) $^{1}H$ NMR spectra of the pH 10 solution initially (i.e., prior to uranyl hydroxide precipitation) 18 mM in U(VI) and 27 mM in PBTC (cf. Figure S25). $^{31}P$ decoupling causes the splitting arising from heteronuclear $^{1}H$-$^{31}P$ scalar spin-spin coupling to collapse thus simplifying the splitting pattern.

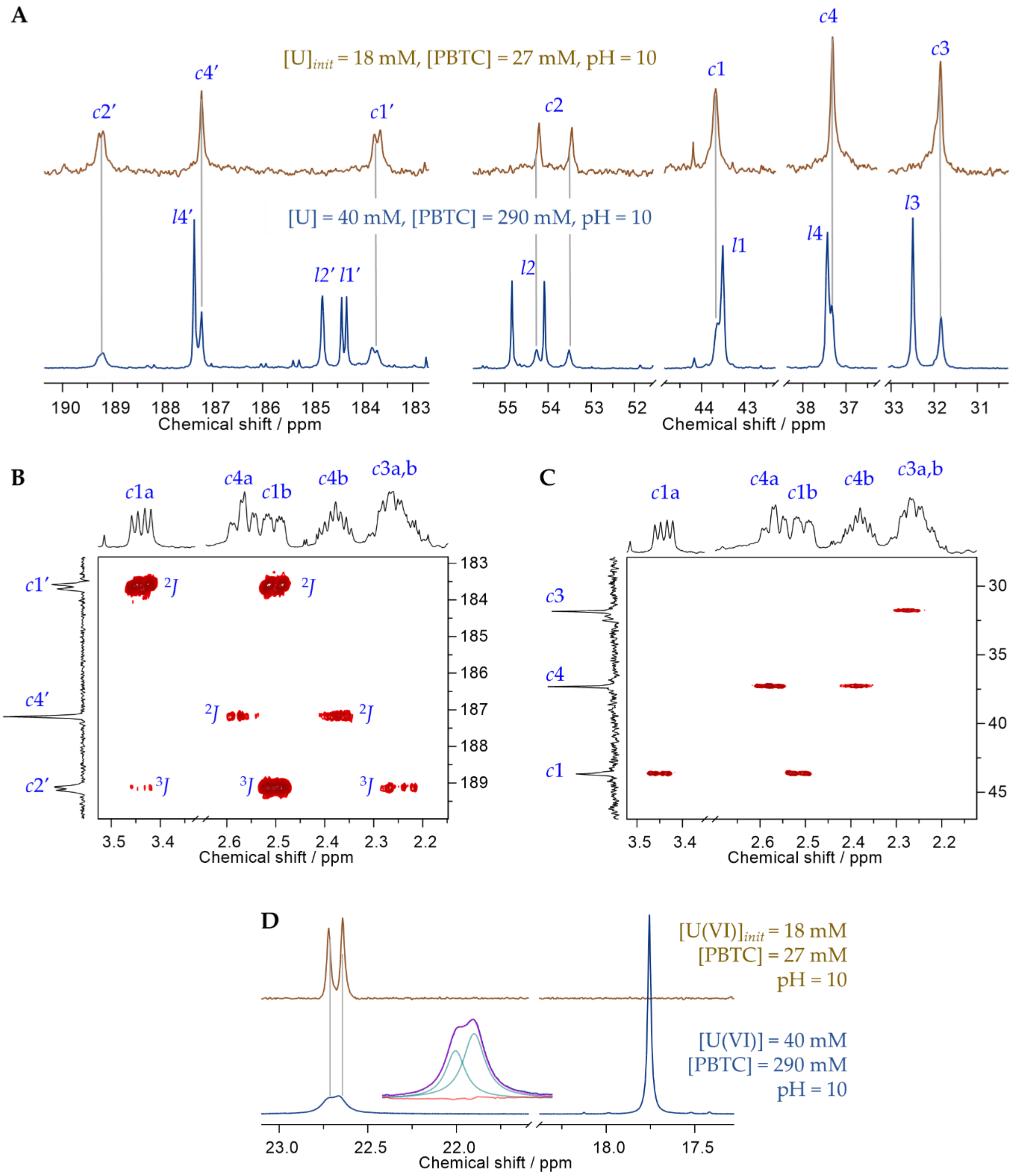


**Figure S25.** $^{13}C\{^1H\}$ (A) and $^{31}P\{^1H\}$ NMR spectra (D) obtained from a pH 10 solution 40 mM in U(VI) and 290 mM in PBTC (blue, bottom spectrum) and a pH 10 solution initially 18 mM in U(VI) and 27 mM in PBTC (brown, top spectrum). In the latter sample, uranyl hydroxide precipitation concomitantly increases the ligand excess resulting in formation of (only) the same 1:2 complex, as evidenced by identical resonances (cf. corresponding $^1H$ NMR spectra in Figure S23). Signal assignment is based on the $^1H,^{13}C$-HMBC (B) and $^1H,^{13}C$-HSQC spectra (C), according to the labeling in Figure 1A, indicating signals of the free ligand and the U(VI) complex by *l* and *c*, respectively.

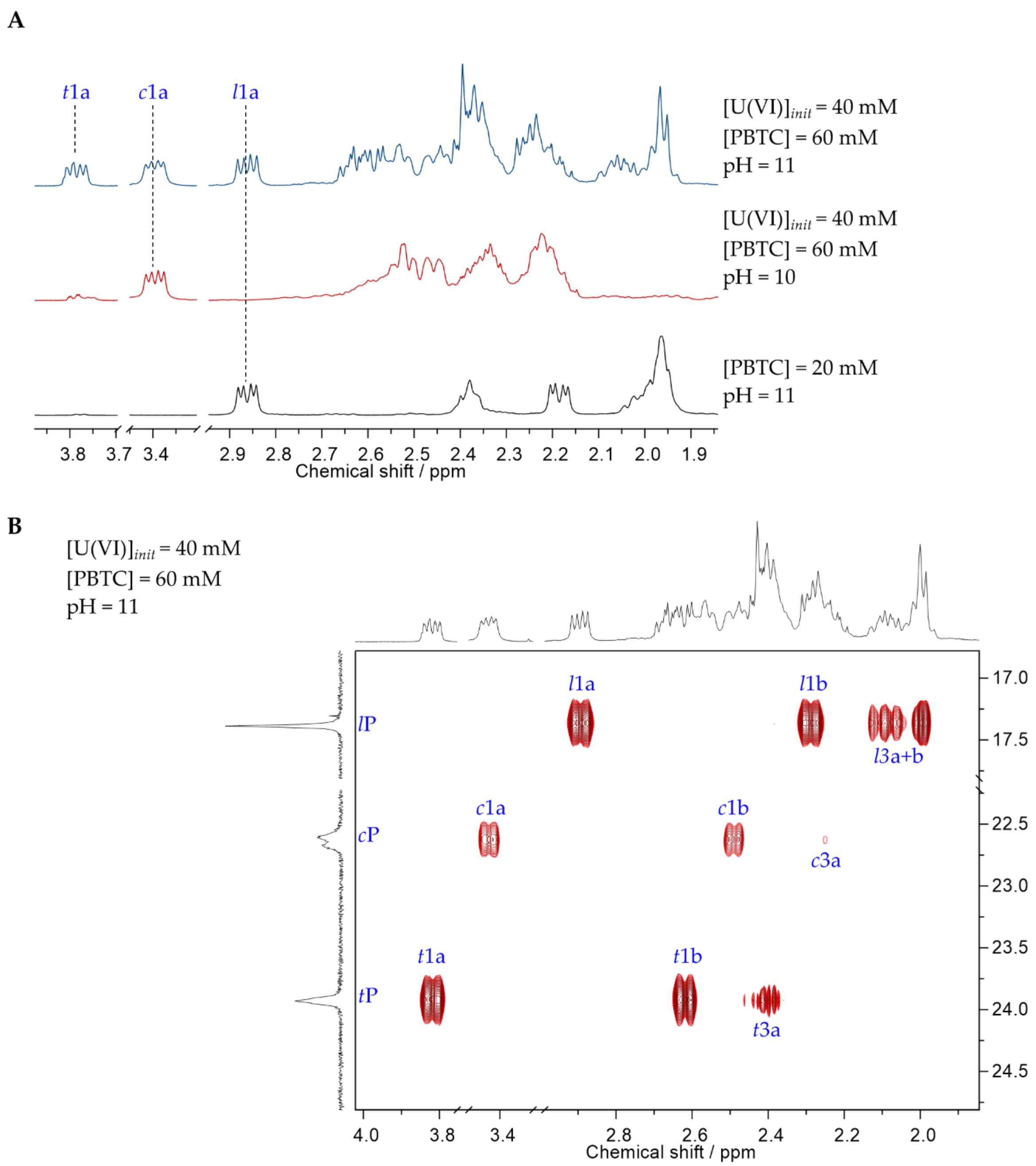


**Figure S26.** (A) $^{1}H$ NMR spectra highlighting the unique signal associated with the ternary U(VI)-PBTC-OH species (*t*1a), obtained from a pH 11 solution initially 40 mM in U(VI) and 60 mM in PBTC (blue spectrum, top), along with spectra displaying almost exclusively signals of the 1:2 complex (red, middle) and free PBTC at corresponding pH (black, bottom). Uranyl removal by hydroxide precipitation concomitantly increases the ligand excess resulting in a final equilibrium with 21 mM U(VI) and 54 mM PBTC, and the observation of free ligand (*l*), the 1:2 complex (*c*), as well as a new set of signals assigned to a ternary complex species (*t*). (B) $^{1}H$,$^{31}P$-HMBC spectrum depicting corresponding $^{31}P$ and characteristic $^{1}H$ NMR signals (1a) due to the individual species (see also Figure 7).

**A**

**B**

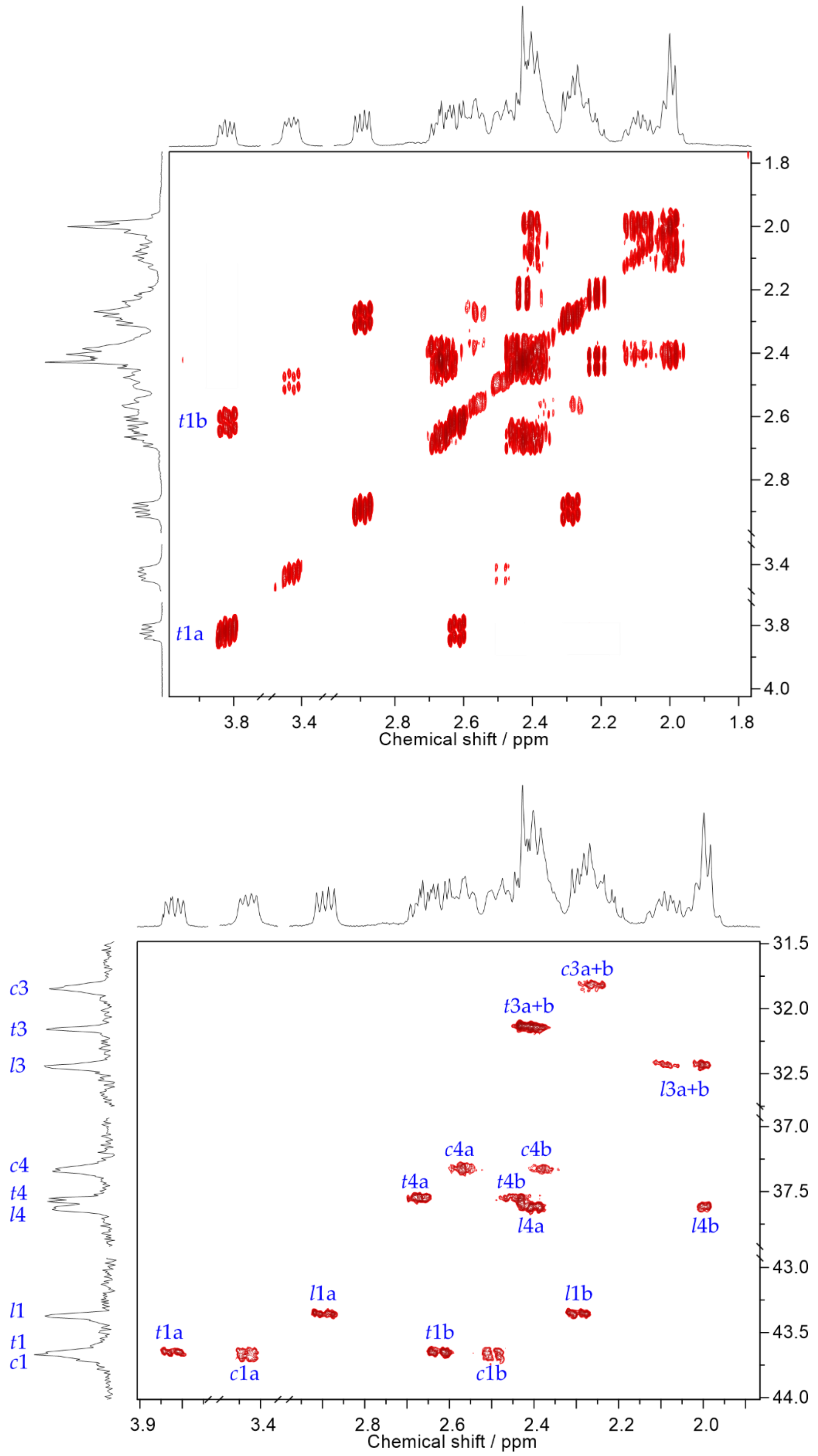


**Figure S27.** $^{1}H,^{1}H$-COSY (A) and $^{1}H,^{13}C$-HSQC NMR spectrum (B) obtained from a pH 11 solution initially 40 mM in U(VI) and 60 mM in PBTC (cf. Figure S26). Uranyl removal by hydroxide precipitation concomitantly increases the ligand excess resulting in a final equilibrium with 21 mM U(VI) and 54 mM PBTC, and the observation of free ligand (*l*), the 1:2 complex (*c*), as well as a new set of signals assigned to a ternary complex species (*t*).

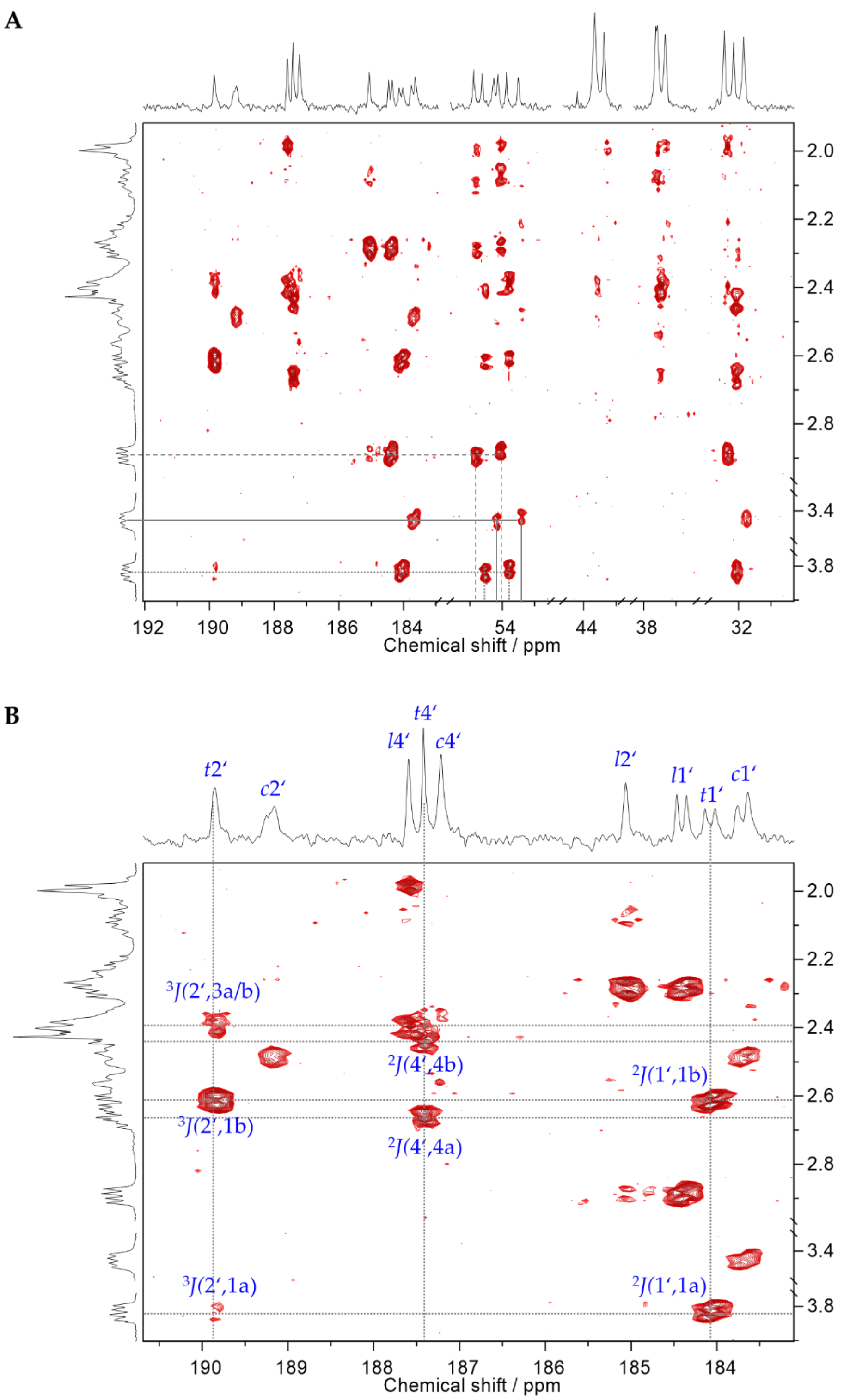


**Figure S28.** Transposed $^{1}H$,$^{13}C$-HMBC NMR spectrum (A) and magnification of the carboxyl carbon region (B) obtained from a pH 11 solution initially 40 mM in U(VI) and 60 mM in PBTC (cf. Figure S26). Uranyl removal by hydroxide precipitation concomitantly increases the ligand excess resulting in a final equilibrium with 21 mM U(VI) and 54 mM PBTC, and the observation of free ligand (*l*), the 1:2 complex (*c*), as well as a new set of signals assigned to a ternary complex species (*t*); see also Figure S29A.

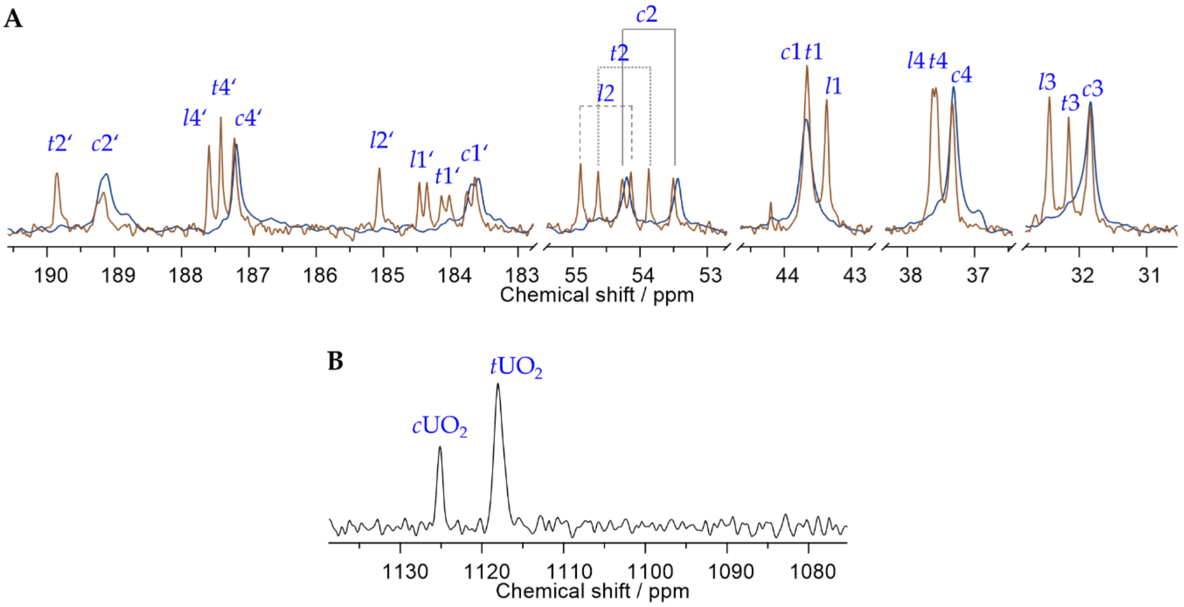


**Figure S29.** (A) Superimposed $^{13}$C{$^{1}$H} NMR spectra obtained from pH 10 (blue spectrum) and pH 11 (brown spectrum) solutions initially 40 mM in U(VI) and 60 mM in PBTC. The former depicts almost exclusively the 1:2 complex (*c*) (cf. Figures S22–S26), while in the latter partial uranyl removal by hydroxide precipitation resulted in a final equilibrium with 21 mM U(VI) and 54 mM PBTC. Thus, the pH 11 spectrum comprises signals of free ligand (*l*), the 1:2 complex (*c*), as well as a new set of signals assigned to a ternary complex species (*t*). The signal assignment is based on the correlation spectra shown in Figures S26–S28. (B) $^{17}$O NMR spectrum acquired at 25 °C, obtained from a pH 11 solution 66 mM in U(VI) (natural $^{17}$O abundance) and 172 mM in PBTC revealing two signals associated with the 1:2 U(VI)-PBTC complex (*c*) and the ternary U(VI)-PBTC-OH species (*t*). These signals are identical to the ones observed in Figure 7 (e.g., the pH 10.9 spectrum), apart from the slight downfield-shift owing to the higher temperature (25 vs. −5 °C, respectively).

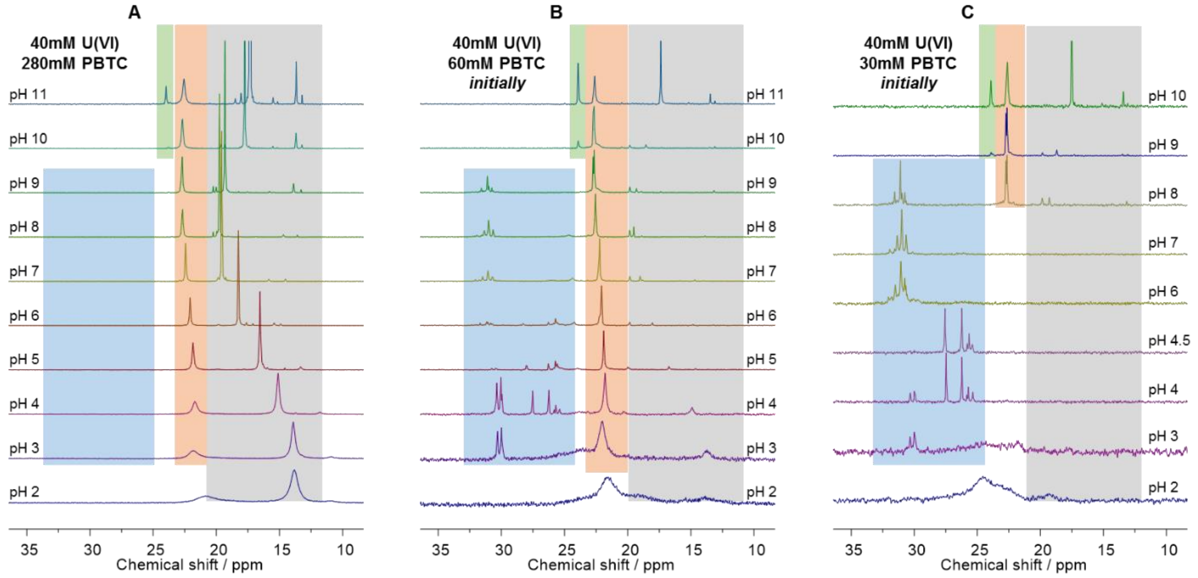


**Figure S30.** Juxtaposition of pH-dependent $^{31}P$ NMR spectra of aqueous solutions of three U(VI):PBTC ratios initially given in solution: 1:7 (A), 1:1.5 (B), and 1:0.75 (C), where the regions highlighted in grey, orange, and green refer to free (excess) PBTC along with impurities, the main 1:2 species in various protonation states of the non-coordinating functional groups, and the ternary 1:2 species, respectively. Note the pH values in C deviating from A and B.

The signals observed in the chemical shift region highlighted in blue are tentatively assigned to polynuclear species, suspected to be dinuclear ($\delta_P \sim 27$ ppm) and trinuclear ($\delta_P \sim 30$ ppm). Figure 30A is identical to Figure 1D in the manuscript, lacking polynuclear species, while B and C display presence and even predominance of polynuclear species, respectively. For the B and especially the C series precipitation was visible in increasing amounts for increasing pH, hence the note for *initial* composition. With reference to SI Figures S23–S26, uranyl(VI) removal from the solutions containing PBTC in concentrations too low to sufficiently stabilize the 1:2 complex, uranyl hydroxide precipitation concomitantly alters the *resulting* U:PBTC ratio in solution with the equilibrium now shifted towards ligand excess. The pH 10 spectrum in Figure S30C clearly demonstrate this effect: although U(VI) was initially in slight excess, upon uranyl hydroxide precipitation the equilibrium is shifted towards stable composition comprising the main 1:2 species, the ternary 1:2 species, and even a significant amount of free PBTC.

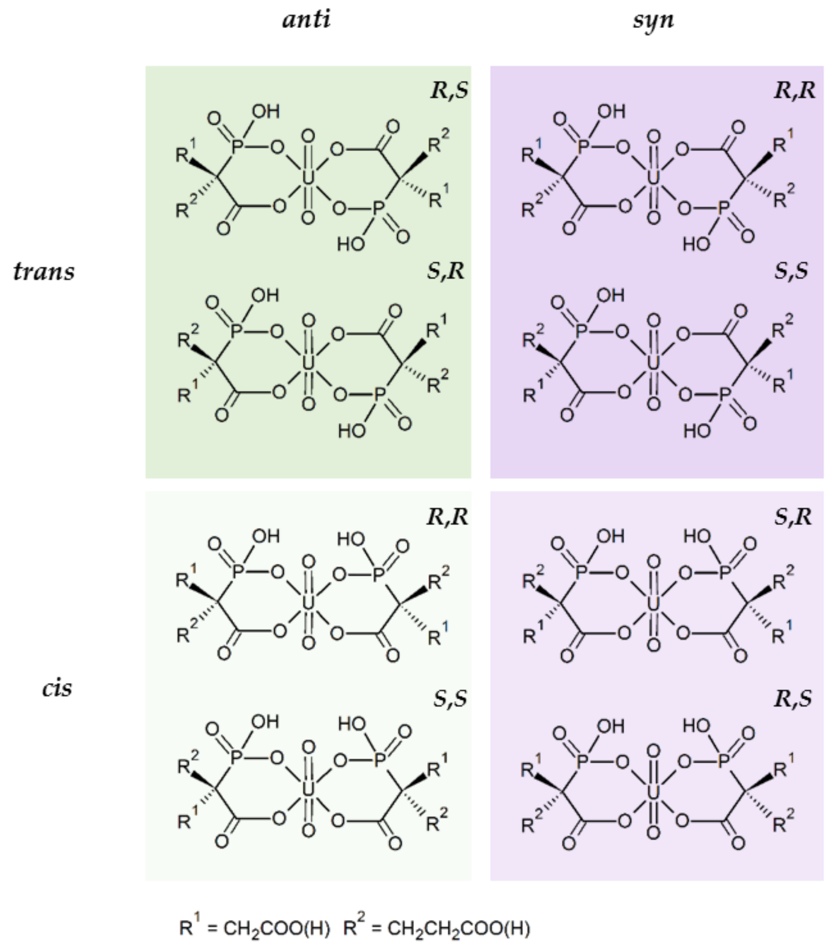


**Figure S31.** Generic structures of the 8 possible ligand arrangements occurring for racemic PBTC in 1:2 U(VI)–PBTC complexes. The terms *syn* and *anti* denote the relative position of the residues $R^1$ and $R^2$, i.e., either on the same side or on opposite sides of the molecular plane, respectively. The terms *cis* and *trans* indicate the arrangement of the two phosphonate groups within one given species. The descriptors *R* and *S* refer to the configuration of the chiral carbon (C2; priority: P > C2' > $R^1$ > $R^2$). These 8 ligand arrangements correspond to 6 different isomers as *R*,*S* = *S*,*R* by rotation.

**Table S5.** Relative Gibbs free energies of various isomers of $UO_2(HPBTC)_2^{6-}$ in kJ/mol.

| HPBTC enantiomers | Ligand arrangement | $G_{rel}$ |
|---|---|---|
| *R*,*R* | *anti* / *cis* | 0.3 |
| | *syn* / *trans* | 0.9 |
| *S*,*S* | *anti* / *cis* | 0.5 |
| | *syn* / *trans* | 0.0 |
| *R*,*S* = *R*,*S* | *anti* / *trans* | 0.0 |
| | *syn* / *cis* | 0.9 |

**DFT – Lowering of p$K_a$ in the complexes (**metal-ion-promoted ligand deprotonation)

To estimate which U(VI)-PBTC complexes prevail at varying pH conditions, we calculated the deprotonation energy of some complexes involving $H_4PBTC$ and $H_3PBTC$ ligands (Table S7). To estimate corresponding p$K_a$ values properly, we relate these results to the known p$K_a$ values of the free PBTC ligand [13]. Our calculated deprotonation energies confirm the order of deprotonation of the functional groups of PBTC as determined previously by experiment and calculations [13]. As shown in Table S6, calculated energies of deprotonation grossly overestimate the measured p$K_a$ values. Thus, by comparison with the experimental values we estimate a correction factor of 5.3 for the second and the higher deprotonation energies of PBTC.

**Table S6.** Estimated ratio of calculated to measured p$K_a$ values.

| Measured p$K_a$ [13] | Calculated Δ$G$ | Calculated p$K_a$ | R[a] |
|---|---|---|---|
| 0.90 | 62.7 | 11.0 | 12.2 |
| 3.92 | 109.4 | 19.2 | 4.9 |
| 4.76 | 144.4 | 25.4 | 5.3 |
| 6.13 | 207.6 | 36.4 | 5.9 |
| 9.79 | 280.4 | 49.2 | 5.0 |
| | | Average[b] | 5.3 |

[a] R = p$K_a^{calculated}$/p$K_a^{measured}$. [b] Average of the four lower and more similar R values.

**Table S7**. Estimated lowering of p$K_a$ values of PBTC ligands in U(VI) complexes. Energies in kJ/mol.

| Species | Δ$G$[a] | ΔΔ$G$[b] | Δp$K_a$/R[c] | p$K_a$[d] |
|---|---|---|---|---|
| $UO_2(H_4PBTC)^+$ | 32 | −77 | −2.6 | 1.3 |
| $UO_2(H_4PBTC)_2^0$ | 41 | −69 | −2.3 | 1.6 |
| $UO_2(H_4PBTC)(H_3PBTC)^-$ | 36 | −74 | −2.4 | 1.5 |
| $UO_2(H_3PBTC)_2^{2-}$ | 108 | −36 | −1.2 | 3.6 |
| $UO_2(H_3PBTC)(H_2PBTC)^{3-}$ | 82 | −62 | −2.1 | 2.7 |

[a] Deprotonation energy according to eq. S2. [b] Difference of Δ$G$ compared to free ligand (Table S6). [c] Calculated lowering of p$K_a$ due to complexation, scaled by the average R value from Table S6. [d] Estimated p$K_a$ of the complexes.

Deprotonation energies of some U(VI)-PBTC complexes containing $H_4PBTC$ and $H_3PBTC$, which are expected at lower pH conditions, have been calculated according to Equation S2

$$UO_2(H_{y1}PBTC)(H_{y2}PBTC)(H_2O)_m{}^q \rightarrow UO_2(H_{(y1-1)}PBTC)(H_{y2}PBTC)(H_2O)_n{}^{q-1} + (m-n-1)\,H_2O + H_3O^+ \quad \text{(Eq. S2)}$$

where y1 = y2 + 1, taking into account for each species the most stable CN. The calculated deprotonation energies Δ$G$ (Table S7) of the least deprotonated ligand of each complex are 40–80 kJ/mol lower than in the free ligand, where they amount to 109 kJ/mol for $H_4PBTC$ and to 144 kJ/mol for $H_3PBTC$ (Table S6). These lower deprotonation energies lead to a calculated lowering of the p$K_a$ for $H_4PBTC$ of 2.3–2.6 logarithmic units and for $H_3BPTC$ to 1.2 and 2.1 logarithmic units by using the scaling factor R = 5.3 (Table S6) to correct for the deviations between calculations and experiment. These changes of p$K_a$ values translate to estimated p$K_a$ values for the U(VI)-PBTC complexes at $I$ = 0.5 M of 1.3–1.6 for the second and of 2.7 and 3.6 for the third deprotonation of a ligand. These results suggest that for pH < 2, 1:1 and 1:2 U(VI)-PBTC complexes with $H_4PBTC$ ligands are the dominant species, but not anymore for the experimental conditions of pH ≥ 2. Thus, the dominant species spectroscopically detected for pH = 2 (Figure S5, right panel) may reliably be interpreted as $UO_2(H_3PBTC)_2^{2-}$.

## U(VI)-PBTC complex formation constants – UV-Vis spectroscopy

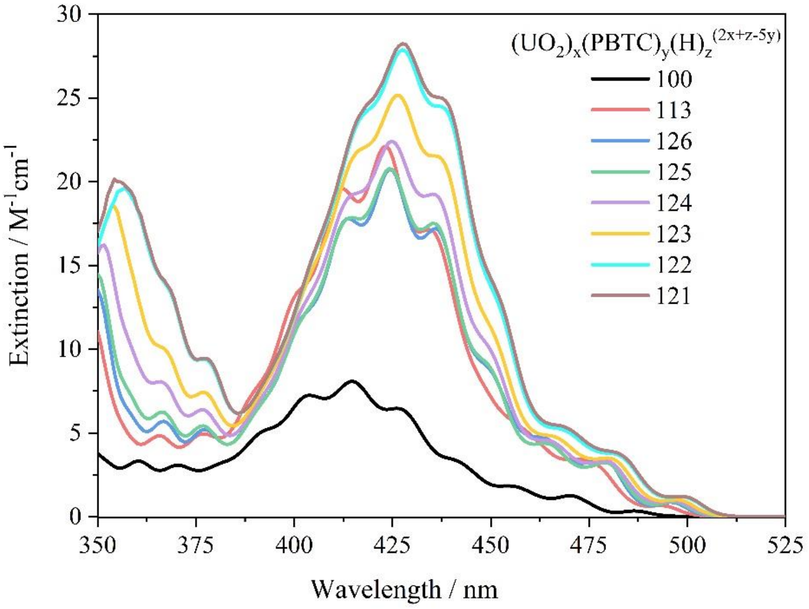


**Figure S32.** Extracted single-component spectra of the individual $(UO_2)_x(PBTC)_y(H)_z$ complex species labeled as xyz.

**Table S8.** Wavelength and corresponding maximum extinction determined from the deconvoluted single-component spectra of the U(VI)-PBTC complex species shown in Figure S32 (0.5 m NaCl, $T$ = (25 ± 1) °C, error 2σ) and, for comparison, of the absorption spectra of free U(VI) at pH 2.

| Species | xyz as in $(UO_2)_x(PBTC)_y(H)_z$ | $\lambda(\varepsilon_{max}) \pm 0.2$ [nm] | $\varepsilon_{max} \pm 0.3$ [$M^{-1}$ $cm^{-1}$] |
|---|---|---|---|
| $UO_2^{2+}$ (aq) | 100 | 414.8 | 8.2 |
| | | 415 | 9.0 [14] |
| | | 415 | 8.4 [15] |
| $UO_2(H_3PBTC)^0$ | 113 | 423.1 | 24.2 |
| $UO_2(H_3PBTC)_2^{2-}$ | 126 | 424.3 | 20.7 |
| $UO_2(H_3PBTC)(H_2PBTC)^{3-}$ | 125 | 424.3 | 20.8 |
| $UO_2(H_2PBTC)_2^{4-}$ | 124 | 424.9 | 22.4 |
| $UO_2(H_2PBTC)(HPBTC)^{5-}$ | 123 | 426.5 | 25.2 |
| $UO_2(HPBTC)_2^{6-}$ | 122 | 427.6 | 27.9 |
| $UO_2(HPBTC)(PBTC)^{7-}$ | 121 | 427.6 | 28.2 |

## References


1. Drobot, B.; Bauer, A.; Steudtner, R.; Tsushima, S.; Bok, F.; Patzschke, M.; Raff, J.; Brendler, V. Speciation studies of metals in trace concentrations: the mononuclear uranyl(VI) hydroxo complexes. *Analytical Chemistry* **2016**, *88*, 3548-3555, doi:10.1021/acs.analchem.5b03958.
2. Demnitz, M.; Hilpmann, S.; Lösch, H.; Bok, F.; Steudtner, R.; Patzschke, M.; Stumpf, T.; Huittinen, N. Temperature-dependent luminescence spectroscopic investigations of uranyl(VI) complexation with the halides $F^-$ and $Cl^-$. *Dalton Transactions* **2020**, *49*, 7109-7122, doi:10.1039/D0DT00646G.
3. Ikeda, A.; Hennig, C.; Rossberg, A.; Tsushima, S.; Scheinost, A.C.; Bernhard, G. Structural determination of individual chemical species in a mixed system by iterative transformation factor analysis-based X-ray absorption spectroscopy combined with UV− Visible absorption and quantum chemical calculation. *Analytical Chemistry* **2008**, *80*, 1102-1110, doi:10.1021/ac7021579.
4. Brinkmann, H.; Patzschke, M.; Kaden, P.; Raiwa, M.; Rossberg, A.; Kloditz, R.; Heim, K.; Moll, H.; Stumpf, T. Complex formation between $UO_2^{2+}$ and α-isosaccharinic acid: insights on a molecular level. *Dalton Transactions* **2019**, *48*, 13440-13457, doi:10.1039/C9DT01080G.
5. Hennig, C.; Servaes, K.; Nockemann, P.; Van Hecke, K.; Van Meervelt, L.; Wouters, J.; Fluyt, L.; Görller-Walrand, C.; Van Deun, R. Species distribution and coordination of uranyl chloro complexes in acetonitrile. *Inorganic Chemistry* **2008**, *47*, 2987-2993, doi:10.1021/ic7014435.
6. Kaiser, H.F. The varimax criterion for analytic rotation in factor analysis. *Psychometrika* **1958**, *23*, 187-200, doi:10.1007/BF02289233
7. Lucks, C.; Rossberg, A.; Tsushima, S.; Foerstendorf, H.; Scheinost, A.C.; Bernhard, G. Aqueous uranium(VI) complexes with acetic and succinic acid: speciation and structure revisited. *Inorganic Chemistry* **2012**, *51*, 12288-12300, doi:10.1021/ic301565p.
8. Malinowski, E.R.; Howery, D.G. *Factor analysis in chemistry*, 3rd ed.; Wiley-Interscience: New York, 2002.
9. Pasilis, S.P.; Pemberton, J.E. Speciation and coordination chemistry of uranyl (VI)− citrate complexes in aqueous solution. *Inorganic Chemistry* **2003**, *42*, 6793-6800, doi:10.1021/ic0341800.
10. Heller, A.; Senwitz, C.; Foerstendorf, H.; Tsushima, S.; Holtmann, L.; Drobot, B.; Kretzschmar, J. Europium(III) meets etidronic acid (HEDP): a coordination study combining spectroscopic, spectrometric, and quantum chemical methods. *Molecules* **2023**, *28*, 4469, doi:10.3390/molecules28114469.
11. Zenobi, M.C.; Luengo, C.V.; Avena, M.J.; Rueda, E.H. An ATR-FTIR study of different phosphonic acids in aqueous solution. *Spectrochim Acta A Mol Biomol Spectrosc* **2008**, *70*, 270-276, doi:10.1016/j.saa.2007.07.043.
12. Jung, W.-S.; Harada, M.; Tomiyasu, H.; Fukutomi, H. Oxygen-17 NMR study of the uranyl ion. V. Kinetics and mechanisms of formation and decomposition reactions of Di-. MU.-hydroxo-bis-[uranyl(VI)] ion in aqueous nitrate solutions. *Bulletin of the Chemical Society of Japan* **1988**, *61*, 3895-3900, doi:10.1246/bcsj.61.3895.
13. Kretzschmar, J.; Wollenberg, A.; Tsushima, S.; Schmeide, K.; Acker, M. 2-Phosphonobutane-1,2,4,-Tricarboxylic Acid (PBTC): pH-Dependent Behavior Studied by Means of Multinuclear NMR Spectroscopy. *Molecules* **2022**, *27*, 4067, doi:10.3390/molecules27134067.
14. Altmaier, M.; Yalçıntaş, E.; Gaona, X.; Neck, V.; Müller, R.; Schlieker, M.; Fanghänel, T. Solubility of U(VI) in chloride solutions. I. The stable oxides/hydroxides in NaCl systems, solubility products, hydrolysis constants and SIT coefficients. *The Journal of Chemical Thermodynamics* **2017**, *114*, 2-13, doi:10.1016/j.jct.2017.05.039.
15. Migdisov, A.A.; Boukhalfa, H.; Timofeev, A.; Runde, W.; Roback, R.; Williams-Jones, A.E. A spectroscopic study of uranyl speciation in chloride-bearing solutions at temperatures up to 250°C. *Geochimica et Cosmochimica Acta* **2018**, *222*, 130-145, doi:10.1016/j.gca.2017.10.016.
